# Synthesis of unsymmetrical disulfanes bearing 1,2,4-triazine scaffold and their in vitro screening towards anti-breast cancer activity

**DOI:** 10.1007/s00706-018-2206-y

**Published:** 2018-06-27

**Authors:** Danuta Branowska, Justyna Ławecka, Mariusz Sobiczewski, Zbigniew Karczmarzyk, Waldemar Wysocki, Ewa Wolińska, Ewa Olender, Barbara Mirosław, Alicja Perzyna, Anna Bielawska, Krzysztof Bielawski

**Affiliations:** 1 Faculty of Science, Siedlce University, 3 Maja 54, 08-110 Siedlce, Poland; 2 Department of Crystallography, Faculty of Chemistry, Maria Curie-Skłodowska University, Pl. Marii Curie-Sklodowskiej 3, 20-031 Lublin, Poland; 3 Department of Medicinal Chemistry and Drug Technology, Medical University of Bialystok, J. Kilinskiego 1, 15-089 Bialystok, Poland

**Keywords:** Anticancer activity, Disulfanes, X-ray structure determination, Conformational analysis, Molecular docking

## Abstract

**Abstract:**

A new series of 1,2,4-triazine unsymmetrical disulfanes were prepared and evaluated as anticancer activity compounds against MCF-7 human breast cancer cells with some of them acting as low micromolar inhibitors. Evaluation of the cytotoxicity using an MTT assay, the inhibition of [^3^H]-thymidine incorporation into DNA demonstrated that these products exhibit cytotoxic effects on breast cancer cells in vitro. The most effective compounds with 59 and 60 µM compared to chlorambucil with 47 µM were disulfanes bearing methyl and methoxy substituent in an aromatic ring. Furthermore, all new 14 compounds were obtained with 22–74% yield via mild and efficient synthesis of the sulfur–sulfur bond formation from thiols and symmetrical disulfanes using 2,3-dichloro-5,6-dicyanobenzoquinone (DDQ). The molecular structure of the newly obtained compounds was confirmed by X-ray analysis. The conformational preferences of disulfide system were characterized using theoretical calculations at DFT level and statistical distributions of C–S–S–C torsion angle values based on the Cambridge Structural Database (CSD). The DFT calculations and CSD searching show two preferential conformations for C–S–S–C torsion angle close to ± 90° and relatively large freedom of rotation on S–S bond in physiological conditions. The molecular docking studies were performed using the human estrogen receptor alpha (ER*α*) as molecular target to find possible binding orientation and intermolecular interactions of investigated disulfanes within the active site of ER*α*. The S…H–S and S…H–C hydrogen bonds between sulfur atoms of bisulfide bridge and S–H and C–H groups of Cys530 and Ala350 as protein residues play crucial role in interaction with estrogen receptor for the most anticancer active disulfane.

**Graphical abstract:**

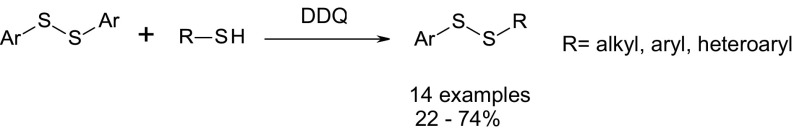

**Electronic supplementary material:**

The online version of this article (10.1007/s00706-018-2206-y) contains supplementary material, which is available to authorized users.

## Introduction

Disulfanes as symmetrical R–S–S–R or as unsymmetrical R–S–S–R′ structure containing S–S bond are found in some natural products like peptides and bioactive molecules [1–5]. Their structure is present in synthetic compounds called fine chemicals [6–11]. They find application as drugs in many civilization diseases as active target molecules such as anticancer [12, 13] and anti-Parkinson [14] diseases are used. Their versatile applications have steered the development of several new methods for the preparation of organic disulfanes. A number of synthetic strategies have been discovered and reported on the sulfur–sulfur bond formation [15–20]. Although there are many different approaches for the preparation of symmetrical disulfanes, most of them are not applicable to the synthesis of unsymmetrical disulfanes, due to the rapid thiol–disulfane exchange reaction or/and the formation of symmetrical side products that are not easily separated from the unsymmetrical disulfanes. The most common route to obtain symmetrical and unsymmetrical disulfanes is oxidation of thiols with halogens [21] and H_2_O_2_ [22, 23]. The second one concerns thiol–disulfane exchange and is important sulfur-based reactions in biology [11]. From the biological view point, since thiol–unsymmetrical disulfane exchange involves negatively charged species, the reaction rate can be influenced by electrostatic factors, such as negative charges adjacent to the reaction center. The situation is more complicated if the symmetrical disulfane is used [24]. The driving force of the reaction is relatively low activation energy required for thiols to break disulfide bonds, which can be accelerated using the additives: bases or oxidants [25]. The disulfane literature is very rich, but there are no reports of a molecular scaffold containing 1,2,4-triazine core. On the other hand, 1,2,4-triazine and its derivatives are an important class of nitrogen aromatic heterocyclic compounds. Some of them are reported to have promising biological activity and used as a drug in medicinal chemistry [26].

Recently, we have reported a series of sulfur 1,2,4-triazine derivatives and evaluated their as anticancer activity compounds against two human breast cancer cell line (MCF-7 and MDA-MB-231) [27]. The most effective compound was 5,5′,6,6′-tetraphenylbis(1,2,4-triazin-3-yl)disulfane acting as low micromolar inhibitors with IC_50_ = 25 µM. These results encouraged us to design and synthesize a larger diversity of disulfanes bearing 1,2,4-triazine scaffold and search for optimized method synthesis. These new compounds were used for in vitro screening towards MCF-7 breast cancer cells. The structural investigations using X-ray analysis were carried out to confirm the synthesis pathway and assumed molecular structures of synthesized disulfanes. The theoretical calculations at ab initio DFT level and Cambridge Structural Database (CSD) searching were performed to find conformational preferences of disulfide system in analyzed compounds, which can be responsible for their biological activity. The possible mode of binding through the identification of the orientation and intermolecular interactions of investigated disulfanes within the active site of the human estrogen receptor alpha (ER*α*) as potential molecular target was characterized by molecular docking studies.

## Results and discussion

### Synthesis of disulfanes 4a–4n

Synthesis of published symmetrical 5,5′,6,6′-tetraphenylbis(1,2,4-triazin-3-yl)disulfane (**2**) was carried out with NBS and 5,6-diphenyl-1,2,4-triazine-3-thiol (**1**) in the standard procedure (Scheme 1) [21]. In the next step, sulfenyl bromide as not isolated intermediate **1a** was reacted with thiol **1** afforded disulfane **2**.

**Figure:**
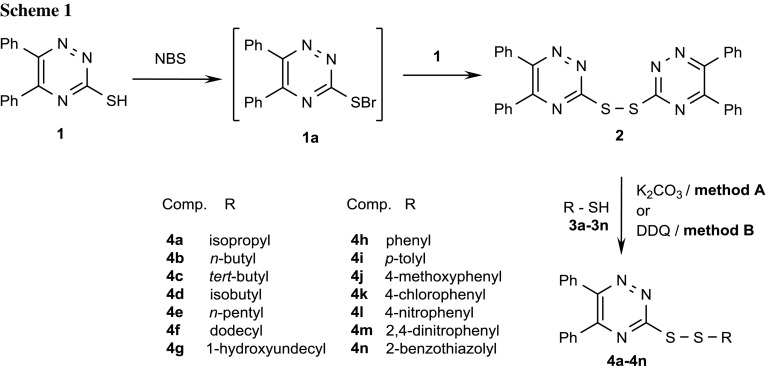

Unfortunately, in our case, this procedure does not worked well for other thiols. With homodisulfane **2** in hand, we decide to explore the exchange method between thiol and disulfane which was mentioned above. The activation of the thiol was performed using at the beginning a method generating thiolate anion via base such as K_2_CO_3_ which reacts quickly with electrophilic part of symmetrical disulfane **2** to produce the corresponding functionalized unsymmetrical disulfide **4** (Table 1). We have found that the yields of expected products **4** (entries 1, 9–11) by this procedure were not satisfying ranging from 19 to 43%. As we can see, yields of obtained products were dependent on the character of applied thiols **3**. The yield was the best for heterodimer **4a** with isopropyl substituent.

**Table 1:** Yields and conditions of the synthesized unsymmetrical disulfanes **4a**–**4n**

Entry	Thiol **3**	Product	Yield/%	
	R	4^a^	K_2_CO_3_ DDQ	
1	-Isopropyl	**4a**	43	40
2	-*n*-Butyl	**4b**	Nd	64
3	-*tert*-Butyl	**4c**	Nd	60
4	-Isobutyl	**4d**	Nd	48
5	-*n*-Pentyl	**4e**	Nd	37
6	-Dodecyl	**4f**	Nd	67
7	-1-Hydroxyundecyl	**4g**	Nd	23
8	-Phenyl	**4h**	Nd	31
9	-*p*-Tolyl	**4i**	24	60
10	-4-Methoxyphenyl	**4j**	19	38
11	-4-Chlorophenyl	**4k**	26	44
12	-4-Nitrophenyl	**4l**	Nd	32
13	-2,4-Dinitrophenyl	**4m**	Nd	22
14	-2-Benzothiazolyl	**4n**	Nd	26
15	-4-Chlorophenyl	**4k**	Nd	74

^a^For compounds **4a**–**4k** substrate is 5,5′,6,6′-tetraphenylbis(1,2,4-triazin-3-yl)disulfane (**2**), for compound **4l** substrate is 4,4′-dinitrophenyl disulfane, for compound **4m** substrate is 2,2′,4,4′-tetranitrophenyl disulfane, for compound **4n** substrate is 2,2′-dithiobis(benzothiazole) as intermediate disulfane

*Nd* not detected

Another reagent applied to the synthesis of exchange reaction thiol–disulfane is popular oxidant DDQ [25]. This method was performed to prepare both symmetrical and unsymmetrical products [28]. We switched our attention to the sulfur–sulfur bond formation from thiols **3** a large variety of substrates bearing diverse functional groups including aliphatic, aromatic, and heteroaromatic groups on 1,2,4-triazine ring.

The reaction of **2** and the commercially available DDQ (0.5 equiv) was first performed in dichloromethane. The reaction was carried out at room temperature for 20 min to furnish dimerized compound **4a** with 40% yield (Table 1, entry 1). Optimization of this reaction conditions for the synthesis of disulfanes was performed using thiol bearing variety of substituents: an electron-donating group (EDG), an electron-withdrawing group (EWG), and without any substituent (Table 1), and with heteroaromatic ring. The results of that studies are summarized in Table 1 and so aromatic ring without any substituent and bearing strong EWG (-NO_2_) functionality present in the starting thiols **3** (Table 1, entries 8, 12, 13) were converted to **4h**, **4l**, and **4m** in moderate yields 31, 32, and 22%, respectively. Aromatic ring with less electron-withdrawing halide (entry 11) gave desired product **4k** in higher yields 44%. Disulfanes containing electron-donating group (–OMe, –Me) (entries 9, 10) could also be dimerized in good yields 60 and 31%. A notable is mentioned that the excess of thiols (2 eq) generally delivered products with less yields with regards to the fact obtained symmetrical disulfanyl products.

Continuing our study, we intended to increase yields of these products using the by-product of 2-mercaptobenzothiazole (BTSH) [2,2′-dithiobis(benzothiazole)] as intermediate disulfide [29], which the high reactivity as good leaving group is documented in the synthesis of unsymmetrical disulfanes [30, 31]. Unfortunately, in this case (Table 1, entry 14), product **4n** was obtained only in 31% yield. We have also observed that the hydroxyl substituted thiol is well tolerated for the formation of S–S bond (Table 1, entry 7) with the yield 23%. Furthermore, our data show that the kind of the substituents on the aromatic rings does not play a vital role in the yield of the final product.

To generalize, when an excess of symmetrical disulfane is used, then the unsymmetrical disulfane may be the major product of the exchange reaction. The success of their method is based on the possibility of separation of symmetrical and unsymmetrical products by column chromatography. The time required to perform this reaction is variable and can vary from few to 24 h. Although this method is not very efficient (yield is usually 22–74%), a wide range of functional groups is tolerated under exchange reaction conditions.

The mechanism of this reaction which has proceeded with DDQ oxidant of all 14 new compounds is not very often discussed. A plausible mechanism synthesis of disulfanes is depicted in Scheme 2. We postulate that the starting thiol is converted to the alkylthio radical (thyil RS) which then reacts with symmetrical disulfane to produce unsymmetrical product **4**. To confirm this radical suppose, we have performed the experiment to use only 0.1 mol% DDQ. In this case, the yield of 1,2,4-triazine unsymmetrical disulfane raised to 74% (Table 1, entry 15). Moreover, the suggested mechanism explains using the catalytic amount of DDQ, which would be sufficient to one single electron transfer (SET).

**Figure:**
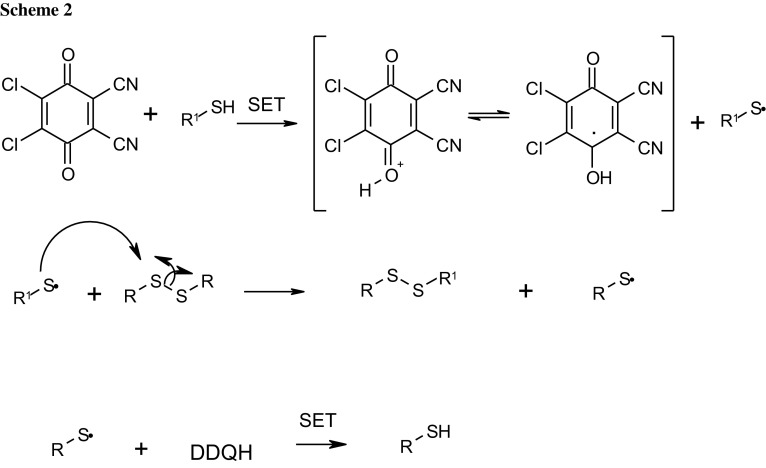

### X-ray analysis

**Fig. 1:**
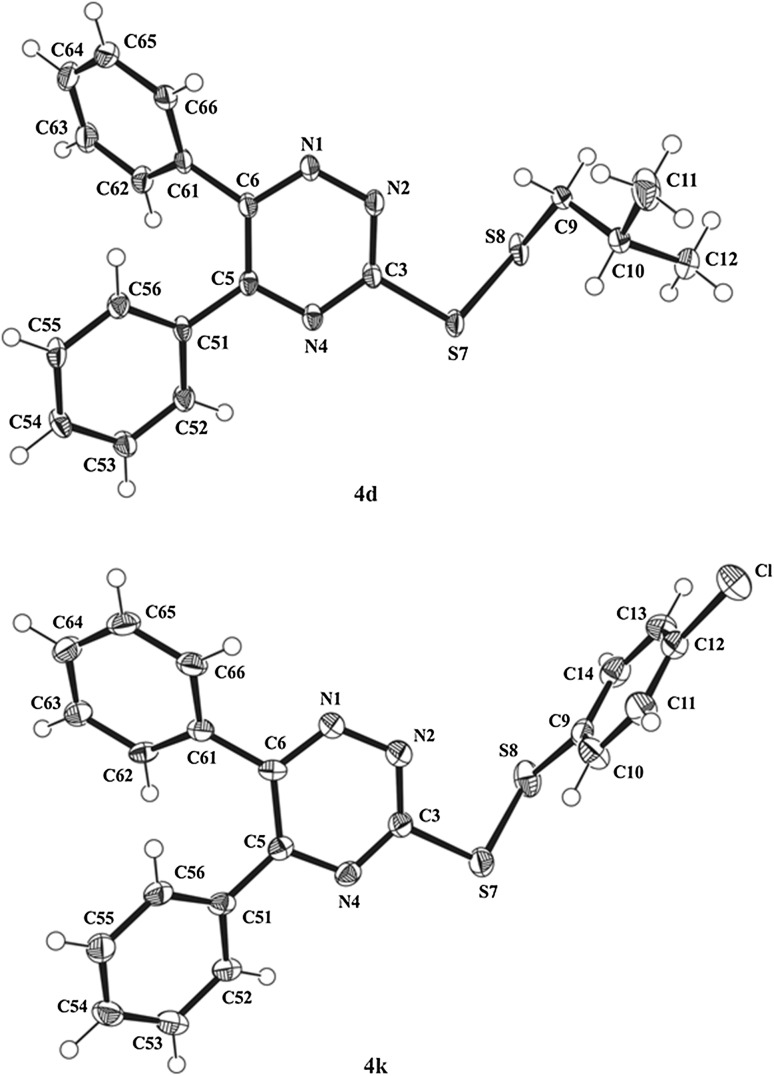
View of the X-ray molecular structures of **4d** and **4k** with the atom-numbering scheme and displacement ellipsoids for non-H atoms drawing at the 50% probability level

The synthesis pathway and assumed molecular structures of the synthesized disulfanes **4a**–**4n** were unambiguously confirmed by X-ray analysis for **4d** with aliphatic and **4k** having aromatic substituent of disulfide system, taken as the model compounds with significantly different anticancer activity. The view of the molecules **4d** and **4k** in the conformation observed in the crystal is shown in Fig. 1.

**Fig. 2:**
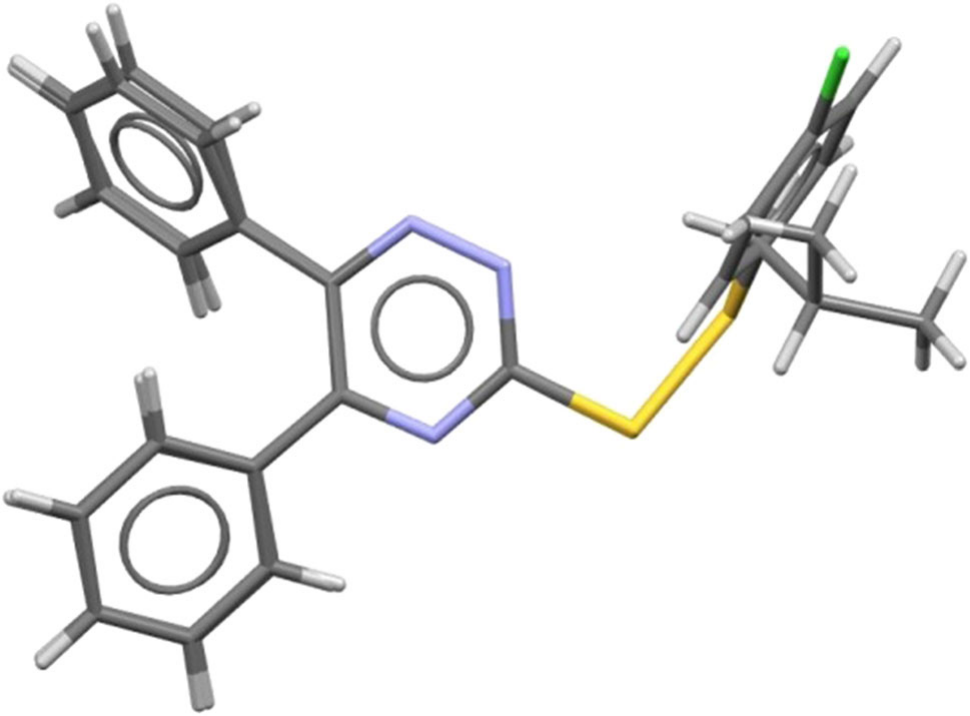
Overlay of X-ray molecules **4d** and **4k** by least-squares fitting of the atoms of 1,2,4-triazine systems (RMS = 0.0198 Å)

The bond distances and angles in investigated molecules are in normal ranges, e.g., the S–S bond lengths of 2.0372(9) Å in **4d** and 2.0271(14) Å in **4k** are comparable with the mean value of 2.031(15) Å observed in similar substructures [32]. The overlay of both molecules by fitting of 1,2,4-triazine systems (Fig. 2) shows that they have very similar conformation.

The torsion angles N4–C5–C51–C52 of − 29.6(3)° in **4d** and − 28.1(4)° in **4k** and N1–C6–C61–C62 of 129.8(2)° in **4d** and 137.1(3)° in **4k** show that the phenyl substituents in 5 and 6 positions of 1,2,4-triazine ring adopt with respect to this ring the *cis* and *gauche* conformation, respectively. In the disulfide system of both molecules, the C–S bonds are nearly perpendicular to each other with the torsion angle C3–S7–S8–C9 of 85.05(12)° for **4d** and 85.77(15)° for **4k**.

**Fig. 3:**
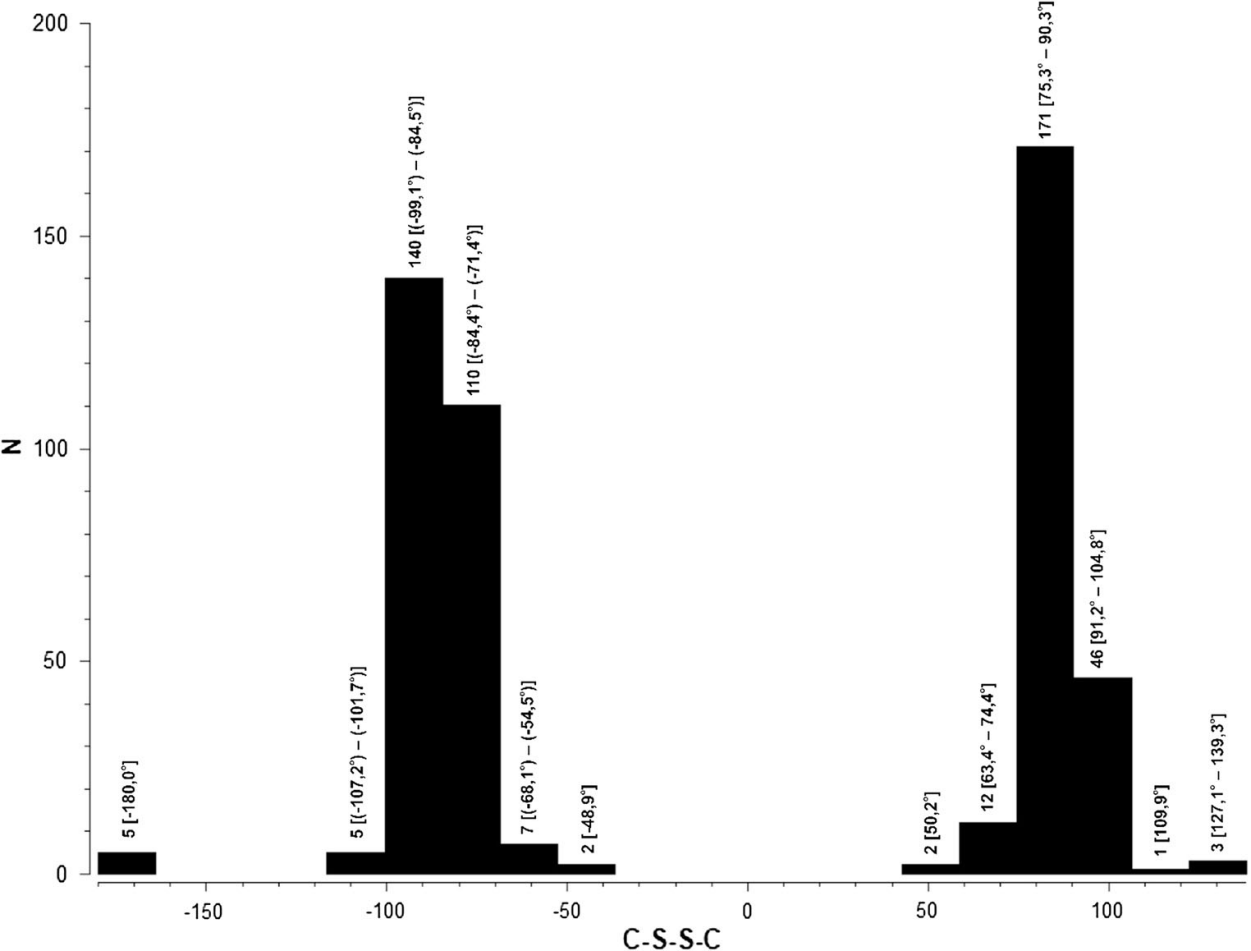
Histogram of torsion angle C–S–S–C in disulfide system

A search of the Cambridge Structural Database (CSD; version 5.38, November 2016 [33, 34]) for the presence of the disulfide system in organic molecules revealed 231 crystal structures and 431 molecules with this system showing only 11 asymmetrical molecules as heterodimers and one structure with 1,2,4-triazine ring bis(5,6-diphenyl-1,2,4-triazine-3-yl)disulfide [35]. The histogram of the torsion angle C–S–S–C observed in the searched molecules is presented in Fig. 3. It is worth noting that, in most cases, the torsion angle at the S–S bond has values close to ± 90°. These values resulting from the mutual perpendicular orientation of the 3*p* orbitals with the lone pairs on the two bonded S atoms are characteristics for the disulfide system [36].

**Fig. 4:**
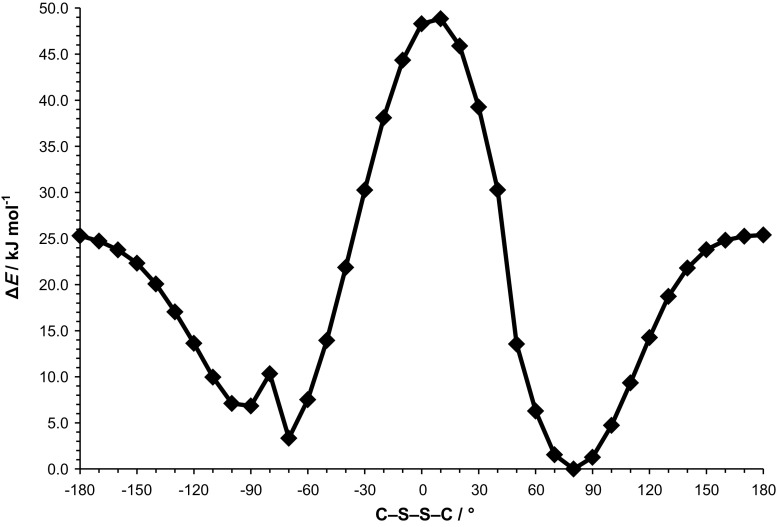
Energy effect upon S–S (C–S–S–C) rotation calculated for **4d** using DFT/B3LYP/6-311++G(*d*,*p*) method

The energy effect of the free rotation at S–S bond for **4d** was calculated using DFT/B3LYP/6-311++G(*d*,*p*) method. The energies of conformations were minimized and all geometrical parameters optimized for each rotation with a 10° increment from − 180° to 180° of C–S–S–C torsion angle (Fig. 4).

The conformations of molecules **4d** and **4k** observed in the crystal are in good agreement with calculated conformations with minima of energy for C–S–S–C of − 70° and + 80°. These two minima are separated by the energy barriers estimated to about 48.8 and 25.4 kJ/mol passing through the maxima of energy at + 10° and ± 180°, respectively, and this does not prevent conformational change between these two minima in a biological target for the molecules of analyzed disulfides. A slight increase in the energy value of C–S–S–C observed at − 90° to − 70° is related to dependence of the rotation energy on the conformation of the isopropyl substituent with respect to the disulfide system. The change of the C–S–S–C torsion angle from − 80° to − 70° causes change of the conformation of the isopropyl substituent described by the torsion angle S–S–C–H from − 54.3° to + 58.6°. This effect causes a slight increase in the energy of the molecule for C–S–S–C of − 80°. Similar results were obtained from theoretical calculations performed for **4k** (Fig. S1, Supplementary Material).

In the crystals, the intermolecular hydrogen bonds are not observed due to the lack of the classical proton-donor groups in the molecules of **4d** and **4k**. The molecular packing is governed by van der Waals forces and weak C–H…π interactions only.

### Cytotoxic activity of **4a**–**4n**

The viability of MCF-7 breast cancer cells was measured by the method of Carmichael using 3-(4,5-dimethylthiazol-2-yl)-2,5-diphenyltetrazolium [37] (Table 2). In terms of reduction in cell viability, the compounds rank in MCF-7 cells in the order chlorambucil > **4j** > **4i** > **4h** > **4n** > **4k** > **4l** > **4a** > **4g** > **4b**–**4f**. Among these derivatives, compound **4j** proved to be only slightly less potent than chlorambucil, with IC_50_ values of 102 ± 2 compared to 97 ± 2 µM for chlorambucil.

**Table 2:** Cytotoxicity of chlorambucil and new compounds **4a**–**4n** against MCF-7 breast cancer cells and normal human skin fibroblasts

Compound	IC_50_/µM^a^	
	MCF-7	Fibroblasts
**4b**	> 200	> 200
**4c**	> 200	> 200
**4d**	> 200	> 200
**4e**	> 200	> 200
**4f**	> 200	> 200
**4g**	197 ± 1	> 200
**4a**	190 ± 2	> 200
**4l**	140 ± 1	> 200
**4k**	121 ± 2	> 200
**4n**	119 ± 2	> 200
**4h**	115 ± 2	> 200
**4i**	108 ± 1	> 200
**4j**	102 ± 2	> 200
Chlorambucil	97 ± 2	150 ± 1

^a^Data presented the mean ± SD of each compound from four independent experiments

We studied the effect of compounds **4a**–**4n** and chlorambucil on DNA synthesis in MCF-7 breast cancer cells (Table 3). All the tested compounds showed concentration-dependent activity, yet with different potency. The concentrations of **4b**–**4f** needed to inhibit [^3^H]thymidine incorporation into DNA by 50% (IC_50_) in MCF-7 cells were above 200 µM suggesting low cytotoxic potency compared to chlorambucil (IC_50_ = 47 ± 2 µM). The concentrations of **4g**–**4k** and **4n** needed to 50% reduction in [^3^H]thymidine incorporation into DNA in breast cancer MCF-7 (IC_50_) obtained in the range 59 ± 2 to 188 ± 2 µM. Among the derivatives, compound **4j** proved to be slightly less potent than chlorambucil, with IC_50_ value of 59 ± 2 µM.

**Table 3:** Antiproliferative effects of chlorambucil and new compounds **4a**–**4n** in MCF-7 breast cancer cells as measured by inhibition of [^3^H]-thymidine incorporation into DNA

Compound	IC_50_/µM^a^	
	MCF-7	Fibroblasts
**4b**	> 200	> 200
**4c**	> 200	> 200
**4d**	> 200	> 200
**4e**	> 200	> 200
**4f**	> 200	> 200
**4g**	188 ± 1	> 200
**4a**	188 ± 2	> 200
**4l**	142 ± 1	> 200
**4k**	83 ± 1	> 200
**4n**	75 ± 2	> 200
**4h**	68 ± 2	> 200
**4i**	60 ± 2	> 200
**4j**	59 ± 2	> 200
Chlorambucil	47 ± 2	120 ± 1

^a^Data presented the mean ± SD of each compound from four independent experiments

### Molecular docking study

The molecular docking studies were performed for investigated disulfanes using the human estrogen receptor alpha (ER*α*) as their molecular target. Breast cancer is the leading cause of cancer death in women worldwide and about 70% of breast cancers are estrogen receptor ER*α*-positive [38]. ER*α* is a major estrogen receptor (ER) subtype in the mammary epithelium, and plays a critical role in mammary gland biology as well as breast cancer progression. The epithelial breast cancer-derived MCF-7 cell line is one of the most frequently used model systems. Clinically, it has been well documented that ER*α* has great potential to promote breast cancer cell motility and invasion [39] and is the most effective predicator of hormone therapy responsiveness.

The molecular docking study showed that all investigated ligands **4a**–**4n** bind to the active site in the predefined binding pocket of the human estrogen receptor ER*α* with the scoring functions ChemPLP presented in Table 4.

**Table 4:** Results of the scoring functions

Compound	ChemPLP
**4a**	67.07
**4b**	73.26
**4c**	69.13
**4d**	72.79
**4e**	78.04
**4f**	99.20
**4g**	102.03
**4h**	80.96
**4i**	82.53
**4j** (most active)	79.90
**4k**	82.86
**4l**	85.91
**4m**	86.59
**4n**	91.27
Chlorambucil^a^	62.34
Reference ligand IOG	126.58

^a^CSD refcode CLAMBU [40]

**Fig. 5:**
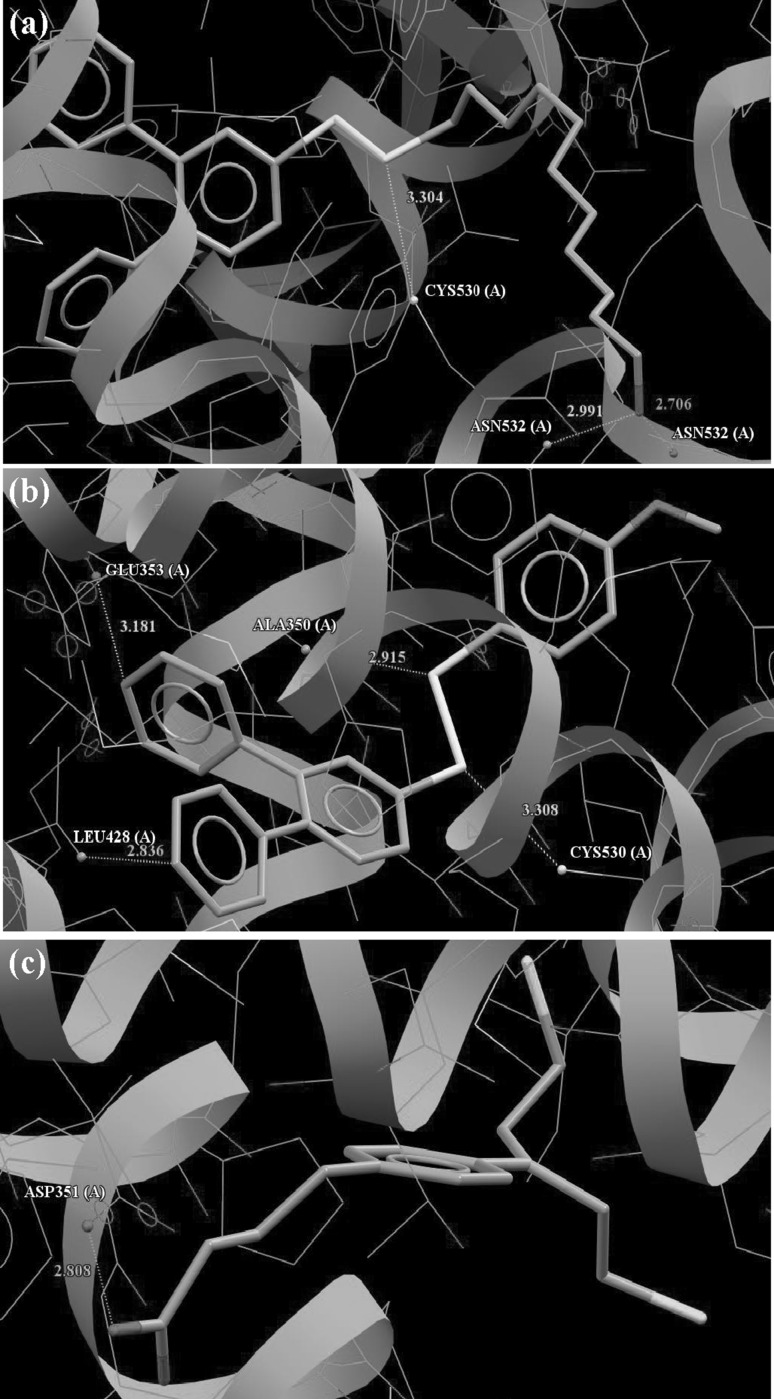
ChemPLP docked poses of **4g** (**a**), **4j** (**b**), and chlorambucil (**c**)

The reference ligand IOG forming complex with ER*α* receptor in the crystalline state gives the best fit for binding pocket linking with the protein mainly through Arg394 (N–H…O) and Glu353 (bifurcated O…O–H) similar to the interactions observed in the crystal. It can be seen that the more pharmacological active compounds **4h**–**4k** with aromatic substituents of disulfide part of molecule have relatively larger values of the fitness function in comparison with practically inactive compounds **4b**–**4e** with alkyl substituents. The exceptions to this rule are the little active compounds **4f** and **4g** with long dodecyl and 1-hydroxyundecyl chain, respectively, and two largest values of scoring ChemPLP function in the investigated series. The best ranked compound **4g** formed hydrogen bonds with Cyst530 (S…H–S) and Asn532 (N–H…O and O…H–O) (Fig. 5a).

The most active in the anticancer tests compound **4j** interacts with the binding domain of ER*α* by Cys530 and Ala350 forming S…H–S and S…H–C hydrogen bonds, respectively, with sulfur atoms of the bisulfide bridge as the proton acceptors. In addition, two weak interactions are observed with Glu353 (O…H–C) and Leu428 (C…H–C), where methine groups belong to phenyl substituent of 1,2,4-triazine ring. The mentioned interactions of **4j** with binding pocket of ER*α* (Fig. 5b) probably play crucial role in joining to estrogen receptor, because this set of the interactions is not observed for the other investigated disulfides. Analysis of the interactions of all ligands from disulfide series shows that the Cys530, Ala350, Thr347, and Asp351 are the most active amino acids in binding domain of ER*α* in the formation of hydrogen bonds. The value of scoring function obtained for chlorambucil (Table 4), which was used as reference compound in pharmacological tests, shows that its molecule is less well fitted to the active side of ER*α* than analyzed disulfides **4a**–**4n** linking with the protein via O–H…O (Asp351) hydrogen bond (Fig. 5c).

This may indicate the different mechanisms of anticancer activity of chlorambucil and investigated disulfides. Chlorambucil, as the commonly used chemotherapeutic drug, interacts with the human glutathione transferases (GSTs) and the human GSTs are the natural targets to form active complexes with chlorambucil [41, 42].

## Conclusions

Our initial attempt was focused to synthesize 1,2,4-triazine scaffolds for SAR studies. In conclusion, we have obtained 14 new disulfanes bearing 1,2,4-triazine ring. We have developed a mild and efficient ‘one-pot’ method for the synthesis of unsymmetrical disulfanes directly from corresponding thiols and symmetrical disulfanes in the presence of DDQ. A broad range of thiols were reacted under optimized reaction conditions. The reaction was tolerant of various substituents including hydroxyl, halides, and methoxy and methyl groups. Aromatic thiols from **3h** to **3n** underwent disulfide formation to furnish desired products with 22–74% yields. Examples of aliphatic disulfanes **4a**–**4g** were synthesized in 36–64% yields. It is noteworthy to mention that the aliphatic thiols underwent reaction in the presence of unprotected hydroxyl group. With the results in mind, we concluded that the reaction was more successful with aliphatic than aromatic substituent. Evaluation of the cytotoxicity using an MTT assay and the inhibition of [^3^H]-thymidine incorporation into DNA demonstrated that these products exhibit cytotoxic effects on breast cancer cells in vitro. The most effective compounds were disulfanes bearing 4-methoxyphenyl (**4j**) and 4-tolylphenyl (**4i**) substituent in disulfide system with micromolar inhibition. These compounds have anticancer activity slightly less than chlorambucil with 59 and 60 µM used as reference compound in biological tests. The X-ray investigations performed for **4d** and **4k** as the model compounds confirmed the synthesis pathway and assumed molecular structures for investigated disulfanes. Moreover, X-ray analysis showed that, in **4d** and **4k,** both C–S bonds in disulfide system are nearly perpendicular to each other giving two possible energetically low conformations for this system. Theoretical calculations at DFT level showed that these two minima of energy are separated by the energy barrier, which value does not prevent molecules against free rotation on S–S bond in physiological conditions. The molecular docking studies performed for investigated disulfanes using the human estrogen receptor alpha (ER*α*) as their molecular target indicated that the sulfur atoms of the bisulfide bridge play important role in interactions with active site of ER*α* with Cys530, Ala350, Thr347, and Asp351 are the most active amino acid residues.

## Experimental

Melting points were determined on Boethius melting point apparatus. The ^1^H NMR spectra were recorded on a Varian Gemini 400 MHz spectrometer with TMS as an internal standard in deuterated solvents. The chemical shifts are given in *δ* (ppm). Mass spectra were measured on AMD 604 spectrometer. Compound **1** was prepared according to the literature procedure [8].

### General procedure for the synthesis of **4a** and **4i**–**4k** using K_2_CO_3_ (method A)

To a mixture of 0.57 mmol of K_2_CO_3_ in 6 cm^3^ of acetone, 0.38 mmol of appropriate thiol was added. The mixture was stirred at room temperature for 5 min. After that, 0.19 mmol 5,5′-6,6′-tetraphenylbis(1,2,4-triazin-3-yl)disulfide (**2**) was added. The mixture was stirred for 2 h (monitoring by TLC). The precipitate was concentrated in vacuum and the crude product was purified by column chromatography on silica gel, using CH_2_Cl_2_ as eluent, to give pure compounds **4a** and **4i**–**4k**.

### General procedure for the synthesis of **4a**–**4k** using DDQ (method B)

To a mixture of 0.25 mmol of 5,5′-6,6′-tetraphenylbis(1,2,4-triazin-3-yl)disulfide (**2**) in 7 cm^3^ of dichloromethane, 0.12 mmol of DDQ and 0.5 mmol of appropriate thiol were added. The mixture was stirred for 1 h (monitoring by TLC). The precipitate was concentrated in vacuum and the crude product was purified by column chromatography on silica gel, using CH_2_Cl_2_ as eluent, to give pure compounds **4a**–**4k**.

### General procedure for the synthesis of **4l**–**4n** using DDQ (method B)

To a mixture of 0.25 mmol of appropriate disulfide in 7 cm^3^ of acetone, 0.12 mmol of DDQ and 0.5 mmol of 5,6-diphenyl-1,2,4-triazine-3-thiol were added. The mixture was stirred for 1 h (monitoring by TLC). The precipitate was concentrated in vacuum and the crude product was purified by column chromatography on silica gel, using CH_2_Cl_2_ as eluent, to give pure compounds **4l**–**4n**.

#### 5,6-Diphenyl-1,2,4-triazine-3-yl isopropyl disulfide (**4a**, C_18_H_17_N_3_S_2_)

Yield 0.034 g (43%); m.p.: 79 °C; ^1^H NMR (CDCl_3_, 400 MHz): *δ* = 1.41 (d, *J* = 6.8 Hz, 6H, 2xCH_3_), 3.40 (sep, *J* = 6.72 Hz, 1H, CH), 7.26–7.61 (m, 10H, 2xAr) ppm; ^13^C NMR (CDCl_3_, 100 MHz): *δ* = 22.3, 41.2, 128.5, 128.6, 129.4, 129.6, 129.7, 129.9, 131.1, 134.9, 135.0, 155.7, 154.89 ppm; IR (KBr): $\bar{\nu }$ = 414 (S–S), 586, 1329, 1363, 1442, 1485, 1599 (C=C, C=N), 3084 (C-H), 3105 (C–C) cm^−1^; HRMS (ESI): *m/z* calcd for C_18_H_18_N_3_S_2_ ([M + H]^+^) 340.09367, found 340.09329.

#### 5,6-Diphenyl-1,2,4-triazine-3-yl *n*-butyl disulfide (**4b**, C_19_H_19_N_3_S_2_)

Yield 0.056 g (64%); m.p.: 38 °C; ^1^H NMR (CDCl_3_, 400 MHz): *δ* = 0.90 (t, *J* = 7.6 Hz, 3H, CH_3_), 1.46 (sek, *J* = 7.6 Hz, 2H, CH_2_), 1.69–1.79 (m, 2H, CH_2_), 3.01 (t, *J* = 7.6 Hz, 2H, CH_2_), 7.28–7.43 (m, 6H, Ar), 7.55 (dd, *J* = 7.2 Hz, 4H, Ar) ppm; ^13^C NMR (CDCl_3_, 100 MHz): *δ* = 13.6, 21.6, 30.8, 38.4, 128.5, 128.6, 129.4, 129.6, 129.9, 131.1, 135.0, 135.5, 154.9, 155.9, 170.5 ppm; IR (KBr): $\bar{\nu }$ = 414 (S–S), 586, 1329, 1363, 1442, 1485, 1599 (C=C, C=N), 3084 (C-H), 3105 (C–C) cm^−1^; HRMS (ESI): *m/z* calcd for C_19_H_20_N_3_S_2_ ([M + H]^+^) 354.10932, found 354.10927.

#### 5,6-Diphenyl-1,2,4-triazine-3-yl isobutyl disulfide (**4c**, C_19_H_20_N_3_S_2_)

Yield 0.053 g (48%); m.p.: 68 °C; ^1^H NMR (CDCl_3_, 400 MHz): *δ* = 1.05 (d, *J* = 6.8 Hz, 6H, 2xCH_3_), 2.04 (sep, *J* = 6.72 Hz, 1H, CH), 2.92 (d, *J* = 6.8 Hz, 2H, CH_2_), 7.28–7.43 (m, 6H, Ar), 7.55 (dd, *J* = 7.2 Hz, 4H, Ar) ppm; ^13^C NMR (CDCl_3_, 100 MHz): *δ* = 21.7, 21.8, 28.1, 47.9, 128.5, 128.6, 129.4, 129.6, 129.9, 131.1, 134.9, 154.9, 155.8, 170.5 ppm; IR (KBr): $\bar{\nu }$ = 419 (S–S), 571, 1329, 1363, 1442, 1485, 1599 (C=C, C=N), 3087 (C–H), 3106 (C–C) cm^−1^; HRMS (ESI): *m/z* calcd for C_19_H_20_N_3_S_2_ ([M + H]^+^) 354.10932, found 354.10934.

#### 5,6-Diphenyl-1,2,4-triazine-3-yl *tert*-butyl disulfide (**4d**, C_19_H_19_N_3_S_2_)

Yield 0.04 g (60%); m.p.: 118 °C; ^1^H NMR (CDCl_3_, 400 MHz): *δ* = 1.44 (s, 9H, 3xCH_3_), 7.34–7.36 (m, 6H, Ar), 7.55 (dd, *J* = 7.2 Hz, 4H, Ar) ppm; ^13^C NMR (CDCl_3_, 100 MHz): *δ* = 29.9, 49.3, 128.5, 128.6, 129.4, 129.6, 129.6, 131.0, 135.0, 135.1, 154.9, 155.6, 170.4 ppm; IR (KBr): $\bar{\nu }$ = 414 (S–S), 586, 1329, 1363, 1442, 1485, 1599 (C=C, C=N), 3084 (C–H), 3105 (C–C) cm^−1^; HRMS (ESI): *m/z* calcd for C_19_H_20_N_3_S_2_ ([M + H]^+^) 354.10932, found 354.10930.

#### 5,6-Diphenyl-1,2,4-triazine-3-yl *n*-pentyl disulfide (**4e**, C_20_H_21_N_3_S_2_)

Yield 0.044 g (37%); m.p.: 44 °C; ^1^H NMR (CDCl_3_, 400 MHz): *δ* = 0.88 (t, *J* = 7.6 Hz, 3H, CH_3_), 1.28–1.42 (m, 4H, 2xCH_2_), 1.77 (qui, *J* = 7.6 Hz, 2H, CH_2_), 3.00 (t, *J* = 7.6 Hz, 2H, CH_2_), 7.28–7.43 (m, 6H, Ar), 7.55 (d, *J* = 7.2 Hz, 4H, Ar) ppm; ^13^C NMR (CDCl_3_, 100 MHz): *δ* = 13.9, 22.3, 28.5, 30.6, 38.6, 128.5, 128.6, 129.4, 129.6, 129.9, 131.2, 135.0, 135.1, 154.9, 155.8, 170.5 ppm; IR (KBr): $\bar{\nu }$ = 414 (S–S), 586, 1329, 1363, 1442, 1485, 1599 (C=C, C=N), 3084 (C–H), 3105 (C–C) cm^−1^; HRMS (ESI): *m/z* calcd for C_20_H_22_N_3_S_2_ ([M + H]^+^) 368.12497, found 368.12495.

#### 5,6-Diphenyl-1,2,4-triazine-3-yl *n*-dodecyl disulfide (**4f**, C_27_H_35_N_3_S_2_)

Yield 0.078 g (67%); yellow oil; ^1^H NMR (CDCl_3_, 400 MHz): *δ* = 0.83–1.37 (m, 25H, (CH_2_)_11_CH_3_), 7.33–7.34 (m, 6H, Ar), 7.50 (d, *J* = 7.2 Hz, 4H, Ar) ppm; ^13^C NMR (CDCl_3_, 100 MHz): *δ* = 8.9, 12.1, 13.9, 14.3, 22.6, 24.9, 26.6, 27.7, 29.2, 29.3, 31.8, 52.8 128.4, 128.6, 129.3, 129.5, 129.8, 131.0, 135.0, 135.1, 154.8, 155.6, 170.4 ppm; IR (KBr): $\bar{\nu }$ = 414 (S–S), 586, 1329, 1363, 1442, 1485, 1599 (C=C, C=N), 3084 (C–H), 3105 (C–C) cm^−1^; HRMS (ESI): *m/z* calcd for C_27_H_36_N_3_S_2_ ([M + H]^+^) 466.23452, found 466.23458.

#### 5,6-Diphenyl-1,2,4-triazine-3-yl 11-hydroxyundecyl disulfide (**4g**, C_26_H_33_N_3_OS_2_)

Yield 0.027 g (23%); yellow oil; ^1^H NMR (CDCl_3_, 400 MHz): *δ* = 1.25–1.61 (m, 18H, (CH_2_)_7_CH_3_), 3.03 (qui, *J* = 7.4 Hz, 2H, CH_2_), 3.63 (t, *J* = 7.4 Hz, 2H, CH_2_), 7.33–7.42 (m, 6H, Ar), 7.55 (d, *J* = 7.2 Hz, 4H, Ar) ppm; ^13^C NMR (CDCl_3_, 100 MHz): *δ* = 25.6, 28.5, 28.8, 29.1, 29.3, 29.4, 29.5, 29.6, 32.8, 38.6, 63.1, 128.5 128.6, 129.4, 129.6, 129.9, 131.1, 135.0, 154.9, 155.8, 170.4 ppm; IR (KBr): $\bar{\nu }$ = 417 (S–S), 586, 1330, 1362, 1485, 1599 (C=C, C=N), 3084 (C–H), 3105, 3300 (OH) cm^−1^; HRMS (ESI): *m/z* calcd for C_26_H_34_N_3_OS_2_ ([M + H]^+^) 468.21378, found 468.21398.

#### 5,6-Diphenyl-1,2,4-triazine-3-yl phenyl disulfide (**4h**, C_21_H_15_N_3_S_2_)

Yield 0.029 g (31%); m.p.: 153 °C; ^1^H NMR (CDCl_3_, 400 MHz): *δ* = 7.26–7.43 (m, 9H, Ar), 7.51–7.54 (m, 4H, Ar), 7.70–7.72 (m, 2H, Ar) ppm; ^13^C NMR (CDCl_3_, 100 MHz): *δ* = 128.2, 128.5, 128.6, 129.1, 129.4, 129.7, 129.4, 129.9, 131.1, 134.8, 134.9, 136.0, 155.2, 156.0, 169.5 ppm; IR (KBr): $\bar{\nu }$ = 422 (S–S), 586, 1367, 1440, 1485, 1597 (C=C, C=N), 3020, 3055, 3103, 3153 (C–C) cm^−1^; HRMS (ESI): *m/z* calcd for C_21_H_16_N_3_S_2_ ([M + H]^+^) 374.07802, found 374.07782.

#### 5,6-Diphenyl-1,2,4-triazine-3-yl *p*-tolyl disulfide (**4i**, C_22_H_17_N_3_S_2_)

Yield 0.058 g (60%); m.p.: 36 °C; ^1^H NMR (CDCl_3_, 400 MHz): *δ* = 2.32 (s, 3H, CH_3_), 7.12 (d, *J *= 8.4 Hz, 2H, Ar), 7.30–7.43 (m, 6H, Ar), 7.51–7.56 (m, 4H, Ar), 7.63 (m, 2H, Ar) ppm; ^13^C NMR (CDCl_3_, 100 MHz): *δ* = 21.2, 128.5, 128.6, 129.4, 129.6, 129.8, 129.9, 131.1, 132.6, 134.8, 134.8, 138.5, 155.1, 155.9, 169.7 ppm; IR (KBr): $\bar{\nu }$ = 415 (S–S), 528, 1367, 1315, 1440, 1485, 1598 (C=C, C=N), 2987, 3020, 3055 (C–H) cm^−1^; HRMS (ESI): *m/z* calcd for C_22_H_18_N_3_S_2_ ([M + H]^+^) 388.09367, found 388.09369.

#### 5,6-Diphenyl-1,2,4-triazine-3-yl 4-methoxyphenyl disulfide (**4j**, C_22_H_18_N_3_OS_2_)

Yield 0.038 g (38%); yellow oil; ^1^H NMR (CDCl_3_, 400 MHz): *δ* = 3.06 (s, 3H, OCH_3_), 7.00 (d, *J* = 9.2 Hz, 2H, Ar), 7.26–7.53 (m, 10H, Ar), 7.63 (d, *J* = 8.8 Hz, 2H, Ar) ppm; ^13^C NMR (CDCl_3_, 100 MHz): *δ* = 55.4, 115.0, 118.4, 128.4, 128.6, 129.3, 129.4, 129.8, 130.8, 135.1, 135.2, 137.2, 153.8, 155.3, 160.8, 171.6 ppm; IR (KBr): $\bar{\nu }$ = 470 (S–S), 530, 1317, 1335, 1367, 1444, 1473, 1579 (C=C, C=N), 3055, 3068, 3099 (C–H) cm^−1^; HRMS (ESI): *m/z* calcd for C_22_H_18_N_3_OS_2_ ([M + H]^+^) 404.11651, found 404.11623.

#### 5,6-Diphenyl-1,2,4-triazine-3-yl 4-chlorophenyl disulfide (**4k**, C_21_H_14_ClN_3_S_2_)

Yield 0.045 g (44%); m.p.: 140 °C; ^1^H NMR (CDCl_3_, 400 MHz): *δ* = 7.26–7.44 (m, 8H, Ar), 7.51–7.54 (m, 4H, Ar), 7.66 (d, *J* = 6.6 Hz, 2H, Ar) ppm; ^13^C NMR (CDCl_3_, 100 MHz): *δ* = 128.5, 128.7, 129.2, 129.4, 129.7, 129.9, 131.2, 131.6, 134.4, 134.5, 134.7, 134.8, 155.3, 156.1, 169.1 ppm; IR (KBr): $\bar{\nu }$ = 422 (S–S), 586, 756, 1367, 1315, 1440, 1485, 1598 (C=C, C=N), 2987, 3020, 3055 (C–H) cm^−1^; HRMS (ESI): *m/z* calcd for C_21_H_15_ClN_3_S_2_ ([M + H]^+^) 408.03904, found 408.03858.

#### 5,6-Diphenyl-1,2,4-triazine-3-yl 4-nitrophenyl disulfide (**4l**, C_21_H_14_N_4_O_2_S_2_)

Yield 0.03 g (32%); m.p.: 145 °C; ^1^H NMR (CDCl_3_, 400 MHz): *δ* = 7.26–7.53 (m, 10H, Ar), 7.77 (d, *J* = 6.8 Hz, 2H, Ar), 8.18 (d, *J *= 6.8 Hz, 2H, Ar) ppm; ^13^C NMR (CDCl_3_, 100 MHz): *δ* = 124.1, 127.6, 128.6, 128.7, 129.4, 129.8, 129.9, 131.4, 134.5, 134.6, 144.9, 146.8, 155.7, 156.3, 167.9 ppm; IR (KBr): $\bar{\nu }$ = 470 (S–S), 528, 1317, 1330, 1367, 1444, 1473, 1579 (C=C, C=N), 3055, 3068, 3099 (C–H) cm^−1^; HRMS (ESI): *m/z* calcd for C_21_H_15_N_4_O_2_S_2_ ([M + H]^+^) 419.06309, found 419.06241.

#### 5,6-Diphenyl-1,2,4-triazine-3-yl 2,4-dinitrophenyl disulfide (**4m**, C_21_H_13_N_5_O_4_S_2_)

Yield 0.025 g (22%); yellow oil; ^1^H NMR (CDCl_3_, 400 MHz): *δ* = 7.29–7.52 (m, 10H, Ar), 8.35 (s, 1H, Ar), 8.40 (d, *J* = 2.4 Hz, 1H, Ar), 9.15 (d, *J* = 2 Hz, 1H, Ar) ppm; ^13^C NMR (CDCl_3_, 100 MHz): *δ* = 121.4, 127.6, 128.7, 129.2, 129.3, 129.8, 130.1, 131.6, 134.2, 134.4, 144.6, 145.3, 145.9, 155.9, 156.4, 166.7 ppm; IR (KBr): $\bar{\nu }$ = 466 (S–S), 582, 1338, 1386, 1444, 1485, 1593, 3061, 3082, 3097 cm^−1^; HRMS (ESI): *m/z* calcd for C_21_H_14_N_5_O_4_S_2_ ([M + H]^+^) 464.04817, found 464.04776.

#### 5,6-Diphenyl-1,2,4-triazine-3-yl benzothiazol-2-yl disulfide (**4n**, C_22_H_14_N_4_S_3_)

Yield 0.028 g (26%); yellow oil; ^1^H NMR (CDCl_3_, 400 MHz): *δ* = 7.28–7.32 (m, 7H, Ar), 7.35–7.39 (m, 4H, Ar), 7.46–7.48 (m, 3H, Ar) ppm; ^13^C NMR (CDCl_3_, 100 MHz): *δ* = 112.2, 121.3, 121.4, 121.6, 122.6, 124.7, 124.9, 125.3, 126.4, 126.6, 127.2, 129.3, 129.5, 129.9, 130.5, 136.1, 140.2 ppm; IR (KBr): $\bar{\nu }$ = 424 (S–S), 596, 1033, 1078, 1427, 1456, 1496, 1597 (C=C, C=N), 3043, 3080 (C–H), 3113 (C–C) cm^−1^; HRMS (ESI): *m/z* calcd for C_22_H_15_N_4_S_3_ ([M + H]^+^) 431.04534, found 431.04345.

### X-ray structure determinations

X-ray data of **4d** and **4k** were collected on SuperNova X-ray diffractometer equipped with Atlas S2 CCD detector using the mirror-monochromatized CuK_*α*_ radiation (*λ* = 1.54184 Å); *ω* scans; crystal sizes 0.30 × 0.30 × 0.20 mm for **4d** and 0.32 × 0.23 × 0.19 mm for **4k**. The analytical numeric absorption correction [43] was applied; *T*_min_/*T*_max_ of 0.473/1.000 for **4d** and 0.931/0.954 for **4k**. The structures were solved by direct methods using SHELXS-2013/1 [44] and refined by full-matrix least squares with SHELXL-2014/7 [44]. The H atoms were positioned geometrically and treated as riding on their parent C atoms with C–H distances of 0.93 Å (aromatic), 0.98 Å (CH), 0.97 Å (CH_2_), and 0.96 Å (CH_3_). All H atoms were refined with isotropic displacement parameters taken as 1.5 times those of the respective parent atoms. The crystal of **4k** used in X-ray diffractometer measurement proved to be a non-merohedral twin, because for the majority of the disagreeable reflections, $F_{\text{o}}^{2}$ is much greater than $F_{\text{c}}^{2}$. Therefore, in the refinement procedure, the 24 reflections with $F_{\text{o}}^{2}$ ≫ $F_{\text{c}}^{2}$ were removed from the intensity data file. Removing these reflections as those affected by the twinning improved *R* value of 0.0645 to 0.0603. All calculations were performed using WINGX version 2014.1 package [45]. CCDC-1584024 for **4d** and CCDC-1584025 for **4k** contain the supplementary crystallographic data for this paper. These data can be obtained free of charge at http://www.ccdc.cam.ac.uk/conts/retrieving.html [or from the Cambridge Crystallographic Data Centre (CCDC), 12 Union Road, Cambridge CB2 1EZ, UK; fax: +44(0) 1223 336 033; email: deposit@ccdc.cam.ac.uk].

Crystal data of **4d**: C_19_H_19_N_3_S_2_, *M*_*r*_ = 353.49 g mol^−1^, monoclinic, space group P2_1_/n, *a* = 12.6017(6) Å, *b* = 10.2269(3) Å, *c* = 15.0464(7) Å, *β* = 112.653(5)°, *V* = 1789.53(14) Å^3^, *Z* = 4, *D*_calc_ = 1.312 g cm^−3^, *F*(000) = 744, *μ*(Cu Kα) = 2.722 mm^−1^, *T* = 120.01 K, 11499 measured reflections (*θ* range 3.91–76.31°), 3684 unique reflections (*R*_int_ = 0.073), final *R* = 0.057, *wR* = 0.152, *S* = 1.110 for 3139 reflections with *I *> 2*σ*(*I*), Δ*ρ*_max_ = + 0.490, and Δ*ρ*_min_ = − 0.548 e Å^−3^.

Crystal data of **4k**: C_21_H_14_ClN_3_S_2_, *M*_*r*_ = 407.92 g mol^−1^, monoclinic, space group C2/c, *a* = 17.3965(19) Å, *b* = 9.2049(6) Å, *c* = 24.2516(3) Å, *β* = 105.435(13)°, *V* = 3743.4(5) Å^3^, *Z* = 8, *D*_calc_ = 1.448 g cm^−3^, *F*(000) = 1680, *μ*(Cu Kα) = 3.974 mm^−1^, *T* = 120.01 K, 7243 measured reflections (*θ* range 3.78–76.86°), 3712 unique reflections (*R*_int_ = 0.055), final *R* = 0.060, *wR* = 0.159, *S* = 1.092 for 2961 reflections with *I *> 2*σ*(*I*), Δ*ρ*_max_ = + 0.833, and Δ*ρ*_min_ = − 0.531 eÅ^−3^.

### Molecular docking

The crystal structure of the human estrogen receptor alpha (ER*α*) in complex with *N*-[(1*R*)-3-(4-hydroxyphenyl)-1-methylpropyl]-2-[2-phenyl-6-(2-piperidin-1-ylethoxy)-1*H*-indol-3-yl]acetamide (IOG) was downloaded from Protein Data Bank (PDB ID: 2IOG [46]; resolution 1.6 Å) and the molecular docking studies were performed using the GOLD Suite v.5.5 [47]. Preparation of hormone (addition of hydrogens, removal of water molecules, and extraction of original ligand from the protein active site,) were done with GOLD as per default settings. Binding site was determined using the previous knowledge of the original ligand interaction site [46]. The reference ligand (IOG) was removed from X-ray structure of its protein–ligand complex (2IOG) and docked back into its binding site. The RMSD values of 0.5645 for ChemPLP confirmed that prediction of the binding mode was successful. In docking simulations, each ligand was kept flexible, but the amino acid residues of the receptor were held rigid. For the simulation runs, default parameter values were taken. The selection of atoms in the active site within 6 Å of original ligands was chosen as default. The ChemPLP was selected as the scoring function to rank the compounds to be investigated. Solutions and protein–ligand interactions were analyzed using Hermes v1.8.2 [47]. The theoretical calculations were performed at the DFT/B3LYP/311++G(*d*,*p*) [48, 49] level using the Gaussian 03 program [50]. The structures were fully optimized without any symmetry constraints and the initial geometries were built from the crystallographic data of **4d** and **4k**.

### Cell culture

Cultured normal human skin fibroblasts and MCF-7 human breast cancer cells were maintained in DMEM supplemented with 10% fetal bovine serum (FBS), 50 U/cm^3^ penicillin, and 50 μg/cm^3^ streptomycin at 37 °C. Cells were cultured in Costar flasks and subconfluent cells were detached with 0.05% trypsin and 0.02% EDTA in calcium-free phosphate-buffered saline, counted in hemocytometers, and plated at 5 × 10^5^ cells per well of 6-well plates (Nunc) in 2 cm^3^ of growth medium (DMEM without phenol red with 10% CPSR1). Cells reached about 80% of confluency at day 3, and in most cases, such cells were used for the assays.

### Cell viability assay

The assay was performed according to the method of Carmichael [36] using 3-(4,5-dimethylthiazole-2-yl)-2,5-diphenyltetrazolium bromide (MTT). Confluent cells cultured for 24 h with various concentrations of studied compounds in 6-well plates were washed three times with PBS and then incubated for 4 h in 1 cm^3^ of MTT solution (0.5 mg/cm^3^ of PBS) at 37 °C in 5% CO_2_ in an incubator. The medium was removed and 1 cm^3^ of 0.1 mol/cm^3^ HCl in absolute isopropanol was added to the cells attached. Absorbance of converted dye in the living cells was measured at a wavelength of 570 nm. Cell viability of breast cancer cells cultured in the presence of ligands was calculated as percentage of controlled cells.

### DNA synthesis assay

MCF-7 cells were seeded in 6-well plates and were incubated with varying concentrations of **1**–**18** or chlorambucil and 0.5 μCi of [^3^H]-thymidine for 24 h at 37 °C [51]. The cells were then harvested by trypsinization, washed with cold phosphate-buffered saline, and centrifuged for 10 min at 1500 g several times (4–5) until the dpm in the washes were similar to the reagent control. Radioactivity was determined by liquid scintillation counting. [^3^H]-thymidine uptake was expressed as dpm/well.

## Electronic supplementary material

Below is the link to the electronic supplementary material.

Supplementary material 1 (PDF 1693 kb)
