# Supplementary material for: Synthesis of unsymmetrical disulfanes bearing 1,2,4-triazine scaffold and their in vitro screening towards anti-breast cancer activity
Source: Monatsh Chem. 2018 Jun 27;149(8):1409–20. doi: 10.1007/s00706-018-2206-y (PMC6060961; doi:10.1007/s00706-018-2206-y)
Supplement: Supplementary file 1 — Supplementary material 1 (PDF 1693 kb) [file 706_2018_2206_MOESM1_ESM.pdf]

Supporting materials

Synthesis of unsymmetrical disulfanes bearing 1,2,4-triazine scaffold and their in vitro screening towards anti-breast cancer activity

Danuta Branowska[1] ● Justyna Ławecka[1] ● Mariusz Sobiczewski[1] ● Zbigniew Karczmarzyk[1] ● Waldemar Wysocki[1] ● Ewa Wolińska[1] ● Ewa Olender[1] ● Barbara Mirosław[2] ● Alicja Perzyna[1] ● Anna Bielawska[3] ● Krzysztof Bielawski[3]

✉ Waldemar Wysocki
wwysocki@uph.edu.pl

1 Faculty of Science, Siedlce University, 3 Maja 54, 08-110 Siedlce, Poland

2 Department of Crystallography, Faculty of Chemistry, Maria Curie-Skłodowska University, Pl. Marii Curie-Sklodowskiej 3, 20-031 Lublin, Poland

3 Department of Medicinal Chemistry and Drug Technology, Medical University of Bialystok, J. Kilinskiego 1, 15-089 Bialystok, Poland

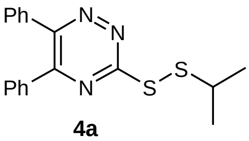

4a

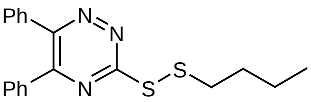

4b

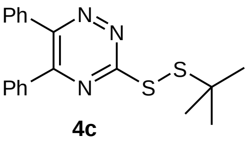

4c

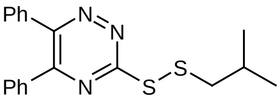

4d

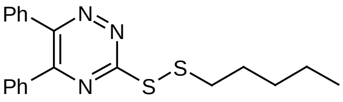

4e

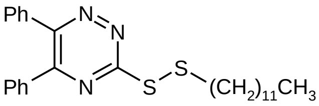

4f

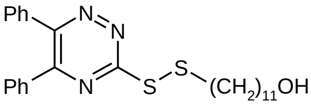

4g

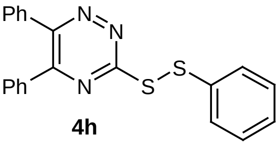

4h

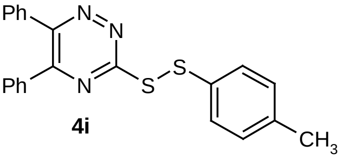

4i

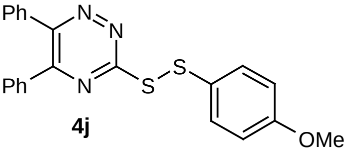

4j

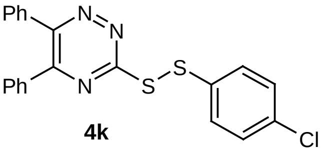

4k

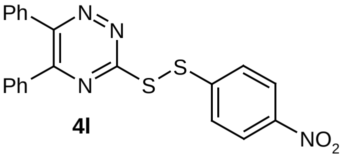

4l

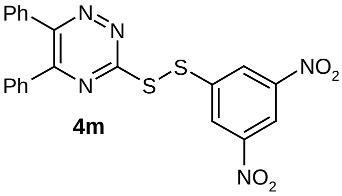

4m

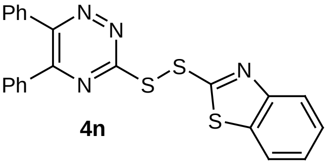

4n

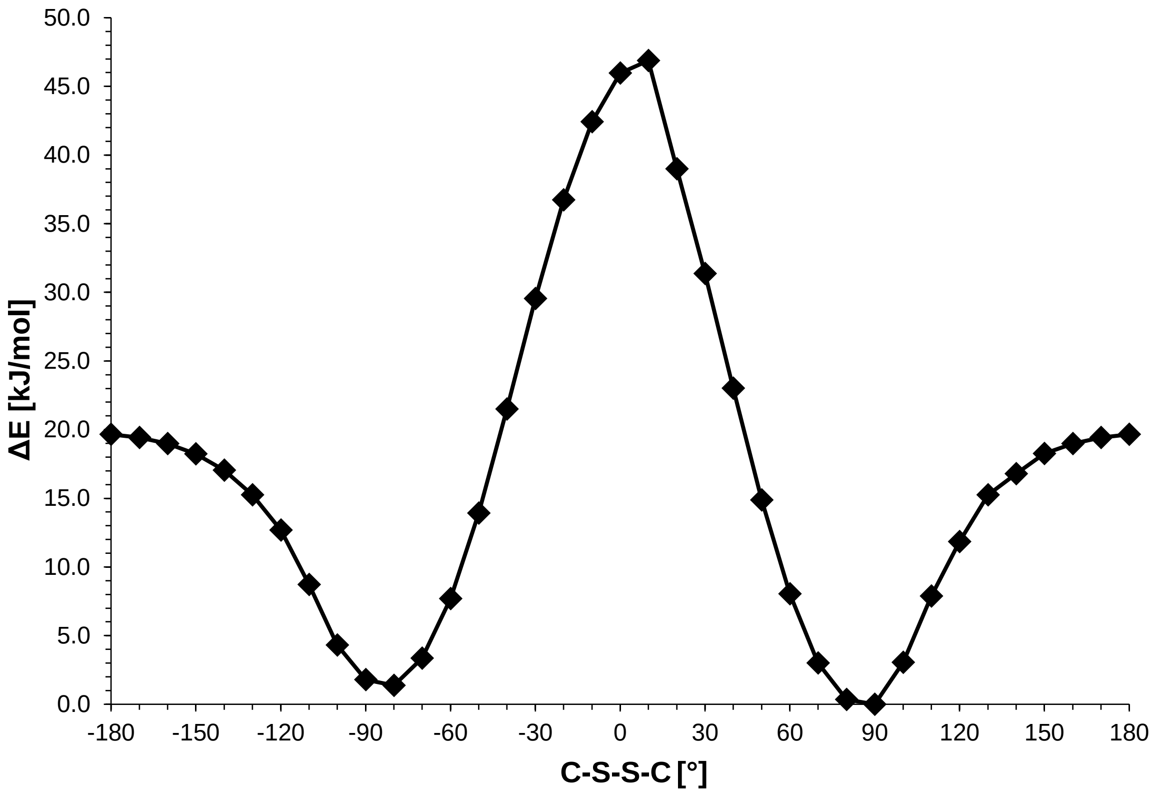

Fig. S1 The energy effect upon S–S (C–S–S–C) rotation calculated for **4k** using DFT/B3LYP/6-311++G(d,p) method

Monatshefte für Chemie - Chemical Monthly
Checklist for compound characterization

- For each compound synthesized or isolated in the manuscript, complete one line below: enter the compound number, state whether the compound is new or known in literature, confirm that you have given it a systematic name, and mark with "x" all columns corresponding to data included in the manuscript.
- For new compounds ^{1}H and ^{13}C NMR data as well as elemental analyses or HRMS data is required; where appropriate, also include a corrected melting point and an R_f value.
- For known compounds either (a) a melting point together with a literature m.p. and the respective reference, or (b) a reference to the respective nmr data have to be given.
- Either complete the checklist electronically, or print the empty list, complete by hand, and scan it to a pdf file. Upload the checklist together with the manuscript as "Electronic Supplementary Material".

Compound				New compounds								Known compounds		
Compound number	Systematic name given	New	Known	^{1}H NMR	^{13}C NMR	IR	HRMS	UV-Vis	Melting point	R_f value	Elemental analysis	Melting point	Reference to melting point in literature	Reference to NMR data in literature
4a	x	x		x	x	x	x		x					
4b	x	x		x	x	x	x		x					
4c	x	x		x	x	x	x		x					
4d	x	x		x	x	x	x		x					
4e	x	x		x	x	x	x		x					
4f	x	x		x	x	x	x							
4g	x	x		x	x	x	x							
4h	x	x		x	x	x	x		x					
4i	x	x		x	x	x	x		x					
4j	x	x		x	x	x	x							
4k	x	x		x	x	x	x		x					
4l	x	x		x	x	x	x		x					
4m	x	x		x	x	x	x							
4h	x	x		x	x	x	x							

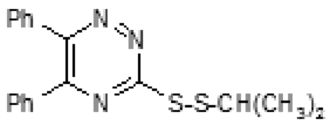

4a

0sk53
1H NMR

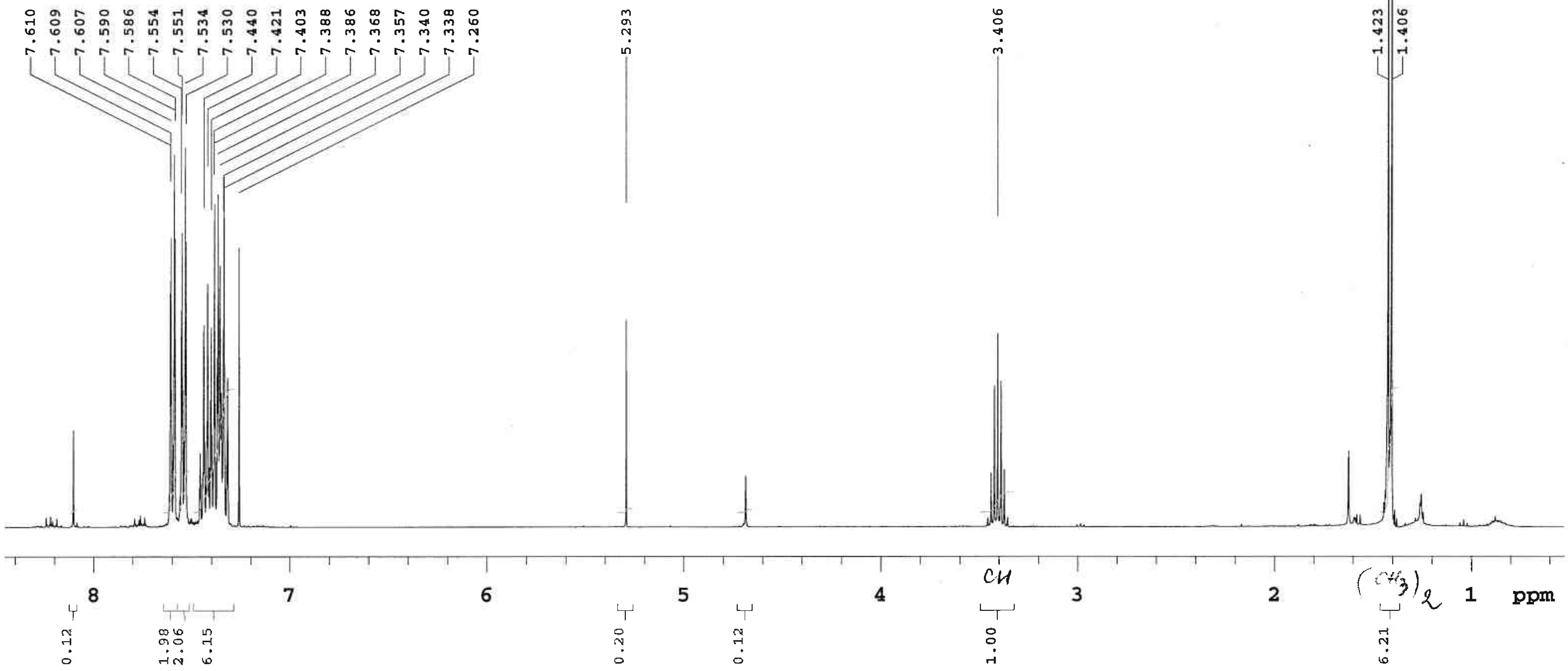

PULSE SEQUENCE
Relax. delay 0.500 sec
Pulse 48.6 degrees
Acq. time 4.797 sec
Width 6793.5 Hz
40 repetitions

OBSERVE H1, 399.6136843

DATA PROCESSING
FT size 131072
Total time 3 minutes

Osk53
in CDCl3

Sample Name:
Osk53
Data Collected on:
400MR-vnmrs400
Archive directory: [illegible] (Z2014

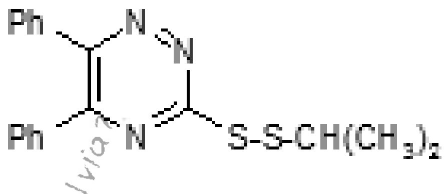

4a

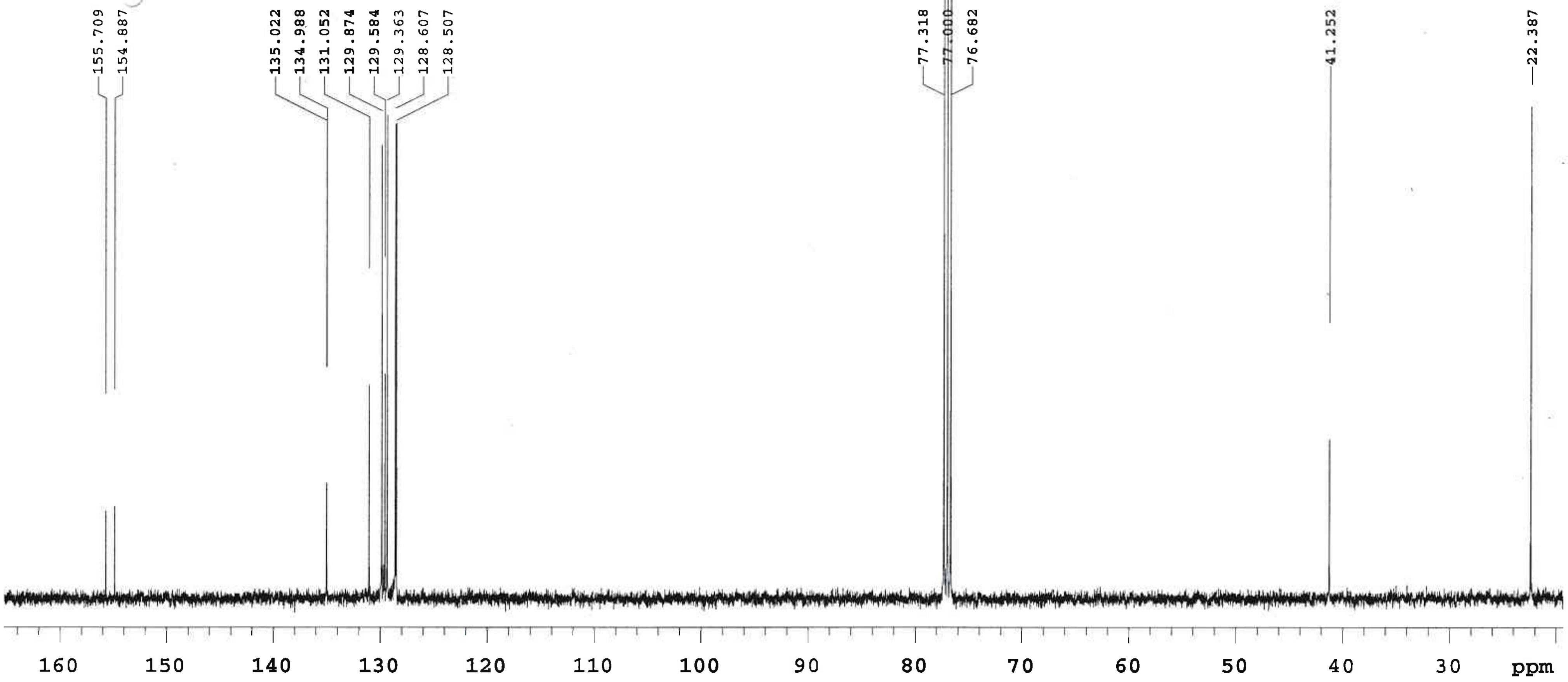

PULSE SEQUENCE
Relax. delay 1.500 sec
Pulse 38.5 degrees
Acq. time 2.000 sec
Width 25510.2 Hz
704 repetitions

OBSERVE C13, 100.4829446
DECOUPLE H1, 399.6156840
Power 36 dB
continuously on
WALTZ-16 modulated

DATA PROCESSING
Line broadening 1.0 Hz
FT size 131072
Total time 41 minutes

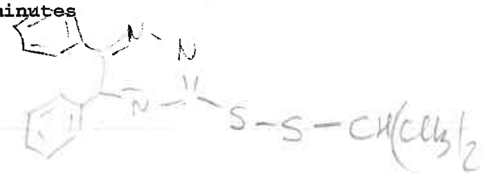

Osk53
in CDCl3

Sample Name:
Osk53
Data Collected on:
400MR-vnmrs400
Archive directory: [illegible]

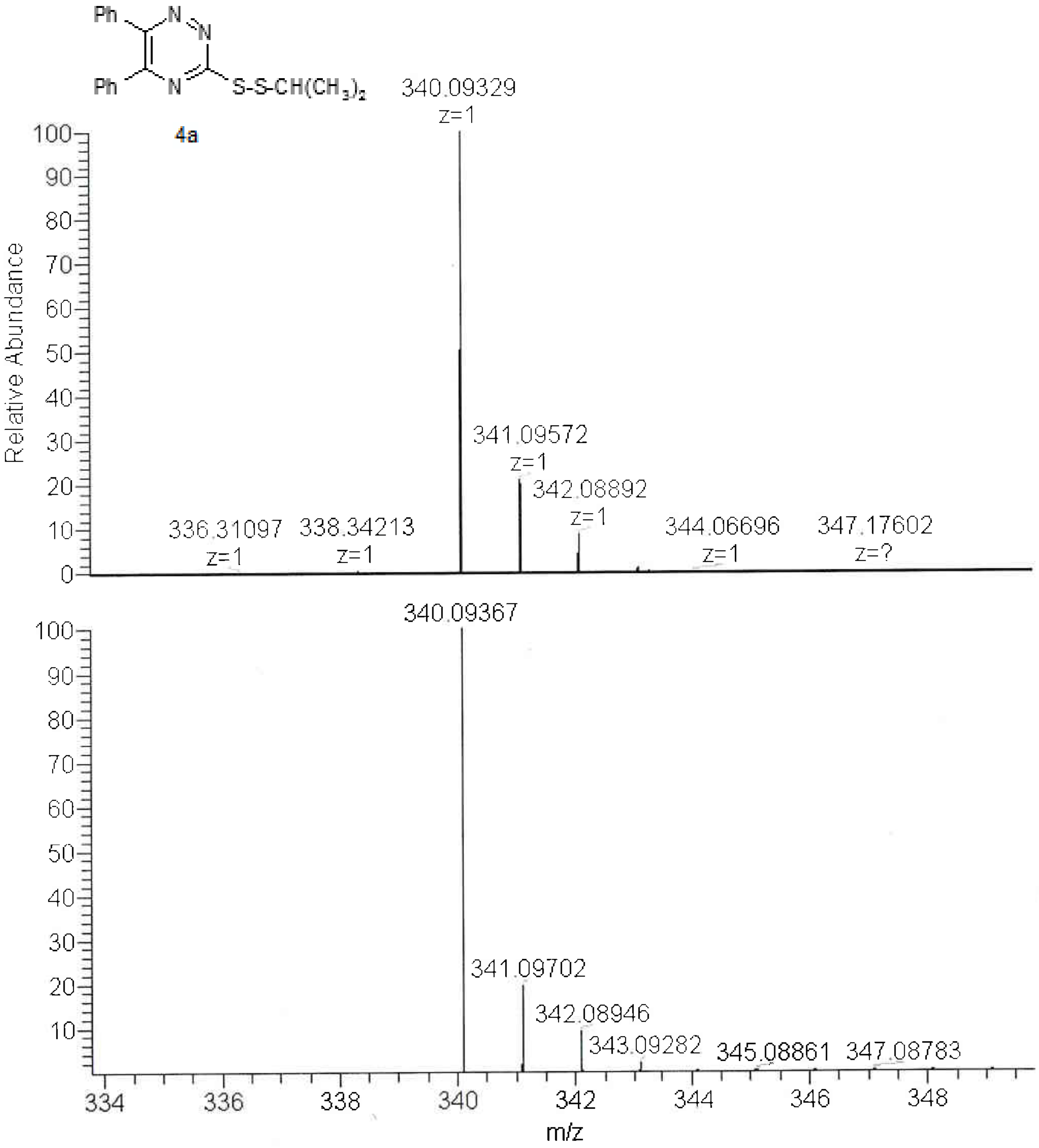
Ph
Ph
N
N
N
S-S-CH(CH3)2
4a
340.09329
z=1
341.09572
z=1
342.08892
z=1
336.31097
z=1
338.34213
z=1
344.06696
z=1
347.17602
z=?
Relative Abundance
NL:
4.23E6
140311_OSK53#45-65 RT: 0.67-0.96
AV: 21 T: FTMS + p ESI Full ms [150.00-2000.00]
340.09367
341.09702
342.08946
343.09282
345.08861
347.08783
NL:
7.33E5
C18 H18 N3 S2:
C18 H18 N3 S2
pa Chrg 1
m/z

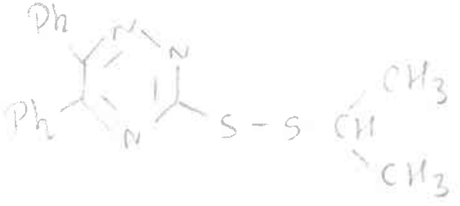

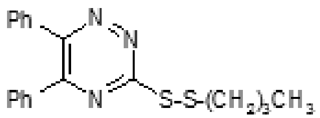

4b

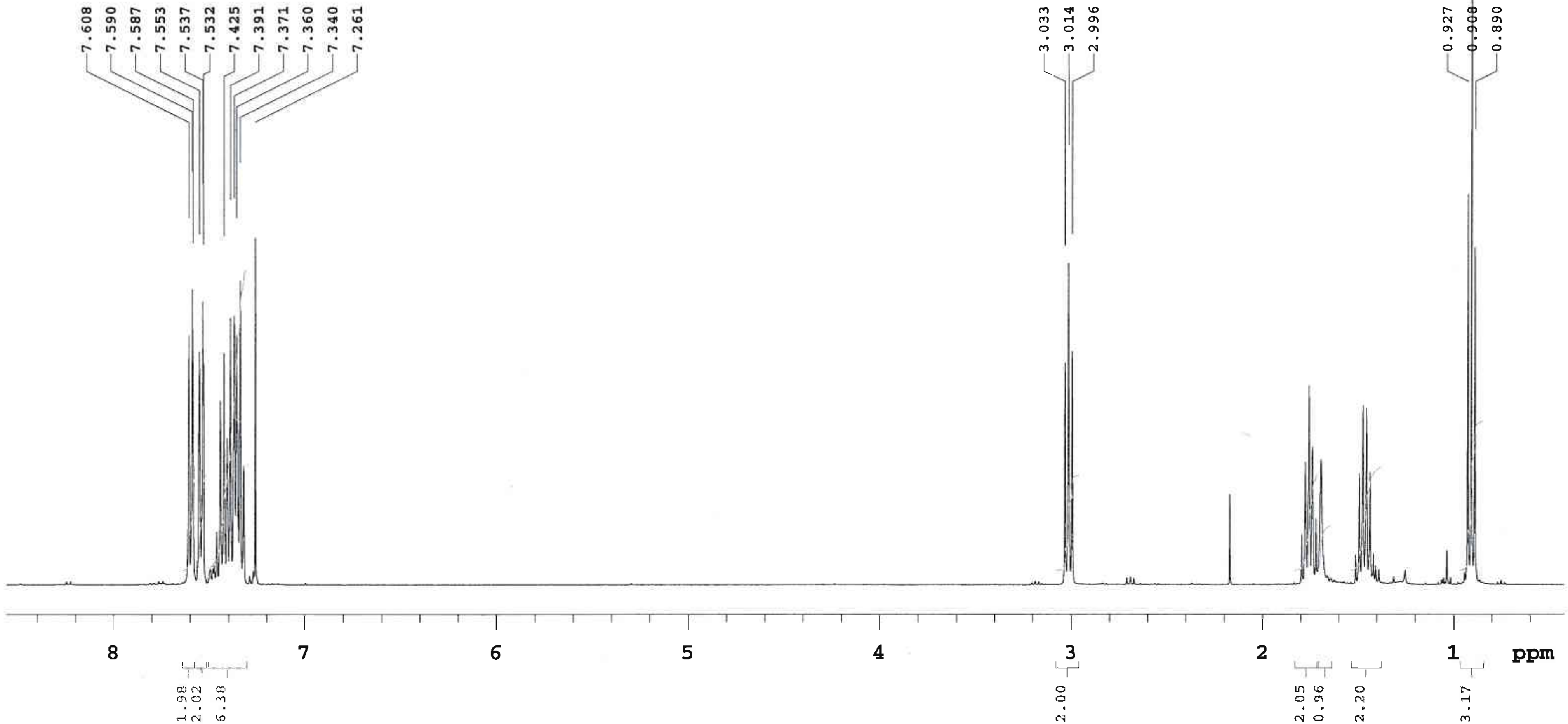

PULSE SEQUENCE
Relax. delay 0.500 sec
Pulse 48.6 degrees
Acq. time 4.797 sec
Width 6793.5 Hz
48 repetitions

OBSERVE H1, 399.6136843

DATA PROCESSING
FT size 65536
Total time 4 minutes

21BA8
in CDCl3

Sample Name:
21BA8
Data Collected on:
400MR-vnmrs400
Archive directory: [illegible]

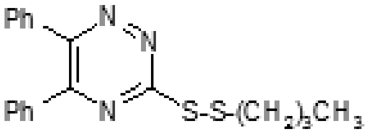

4b

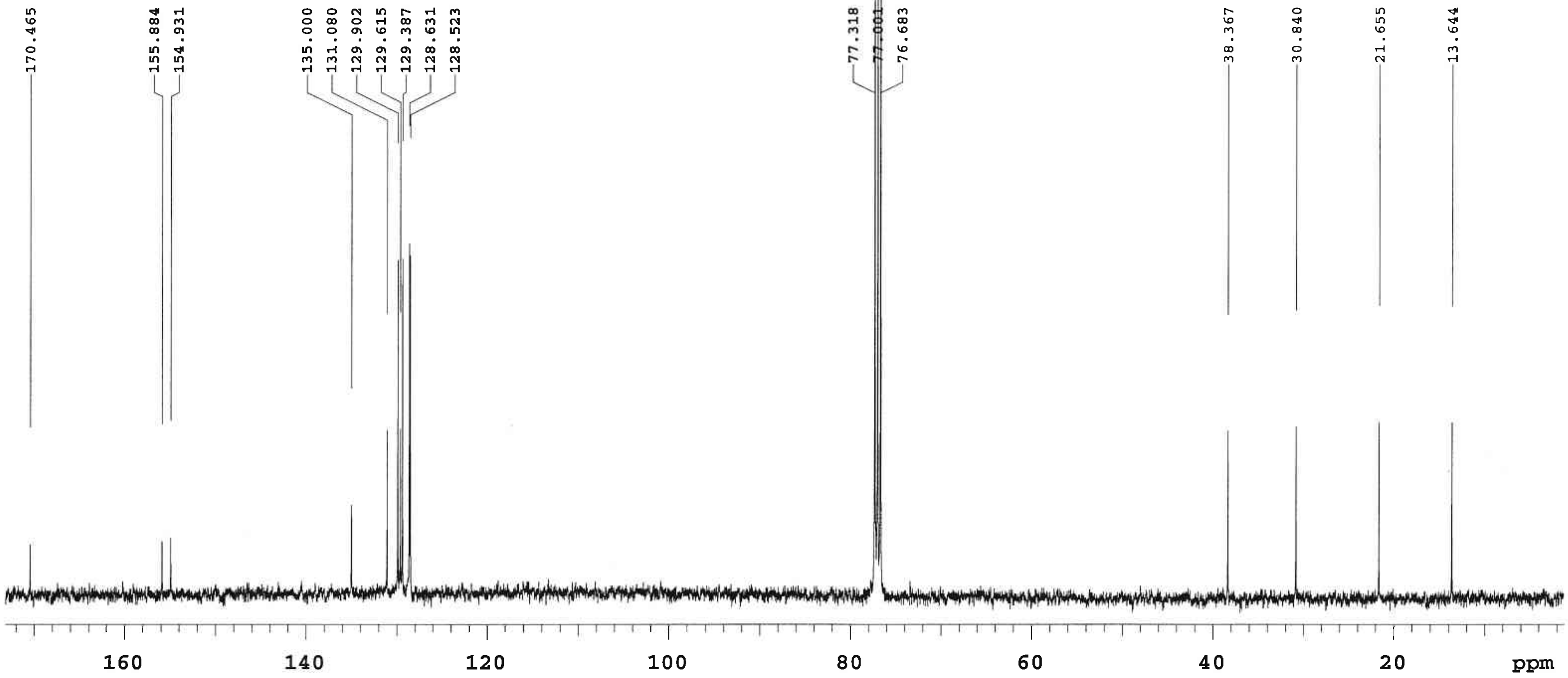

PULSE SEQUENCE
Relax. delay 1.500 sec
Pulse 38.5 degrees
Acq. time 2.000 sec
Width 25510.2 Hz
2336 repetitions

OBSERVE C13, 100.4829425
DECOUPLE H1, 399.6156840
Power 36 dB
continuously on
WALTZ-16 modulated

DATA PROCESSING
Line broadening 2.0 Hz
FT size 131072
Total time 2.3 hours

ZIBA8 w CDCl3

Sample Name:
ZIBA8
Data Collected on:
400MR-vnmrs400
Archive directory:
[illegible]

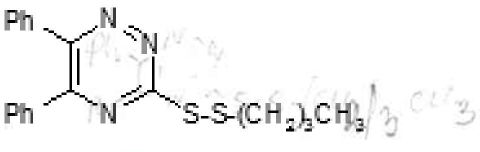
Ph
N
N
Ph
N
S-S-(CH$_2$)$_3$CH$_3$

4b

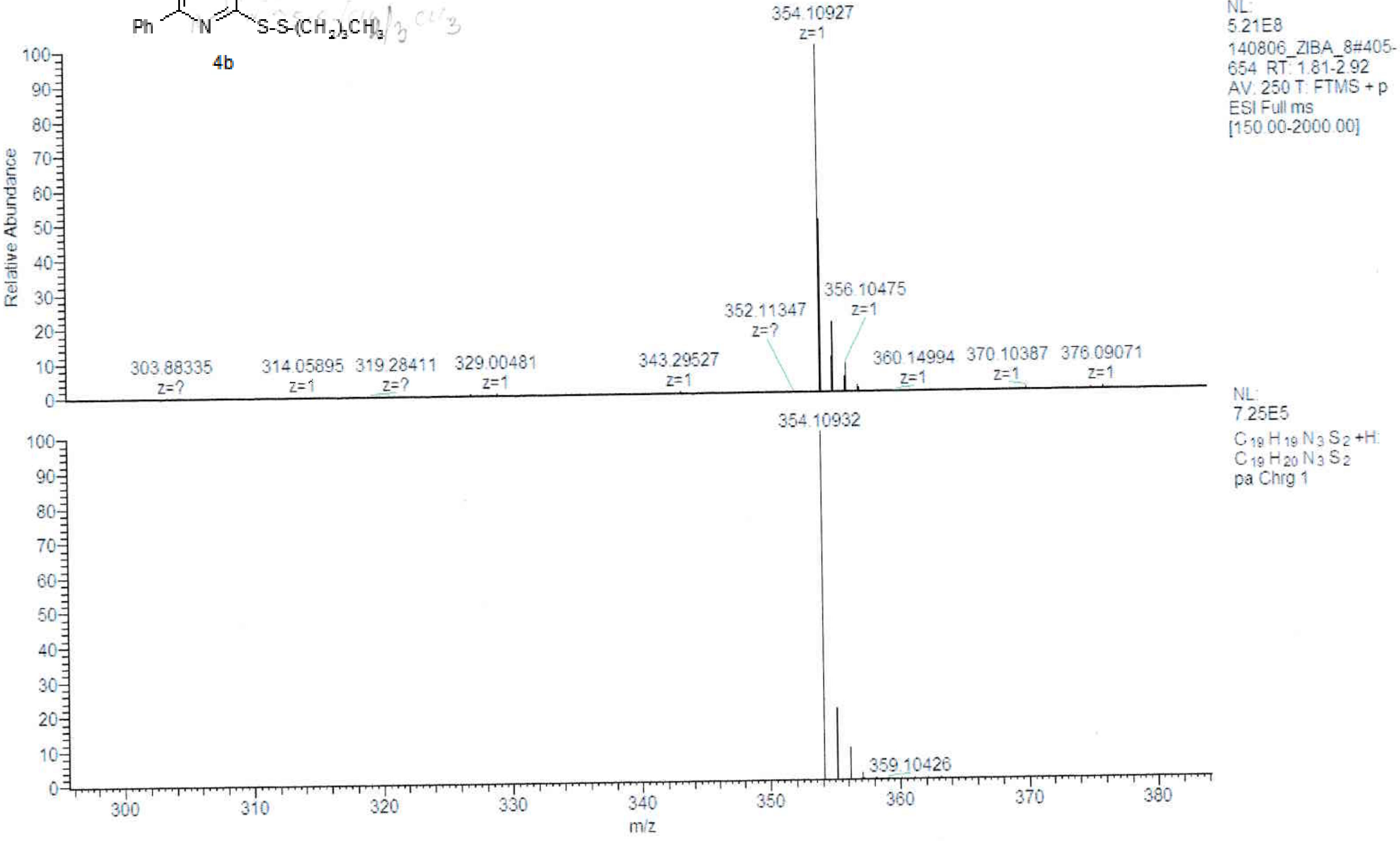
NL:
5.21E8
140806_ZIBA_8#405-654 RT: 1.81-2.92
AV: 250 T: FTMS + p
ESI Full ms
[150.00-2000.00]
354.10927
z=1
356.10475
z=1
352.11347
z=?
303.88335
z=?
314.05895
z=1
319.28411
z=?
329.00481
z=1
343.29527
z=1
360.14994
z=1
370.10387
z=1
376.09071
z=1
Relative Abundance
NL:
7.25E5
C 19 H 19 N 3 S 2 +H:
C 19 H 20 N 3 S 2
pa Chrg 1
354.10932
359.10426
m/z

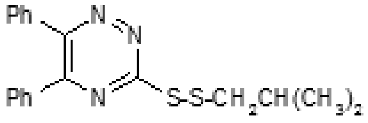

4c

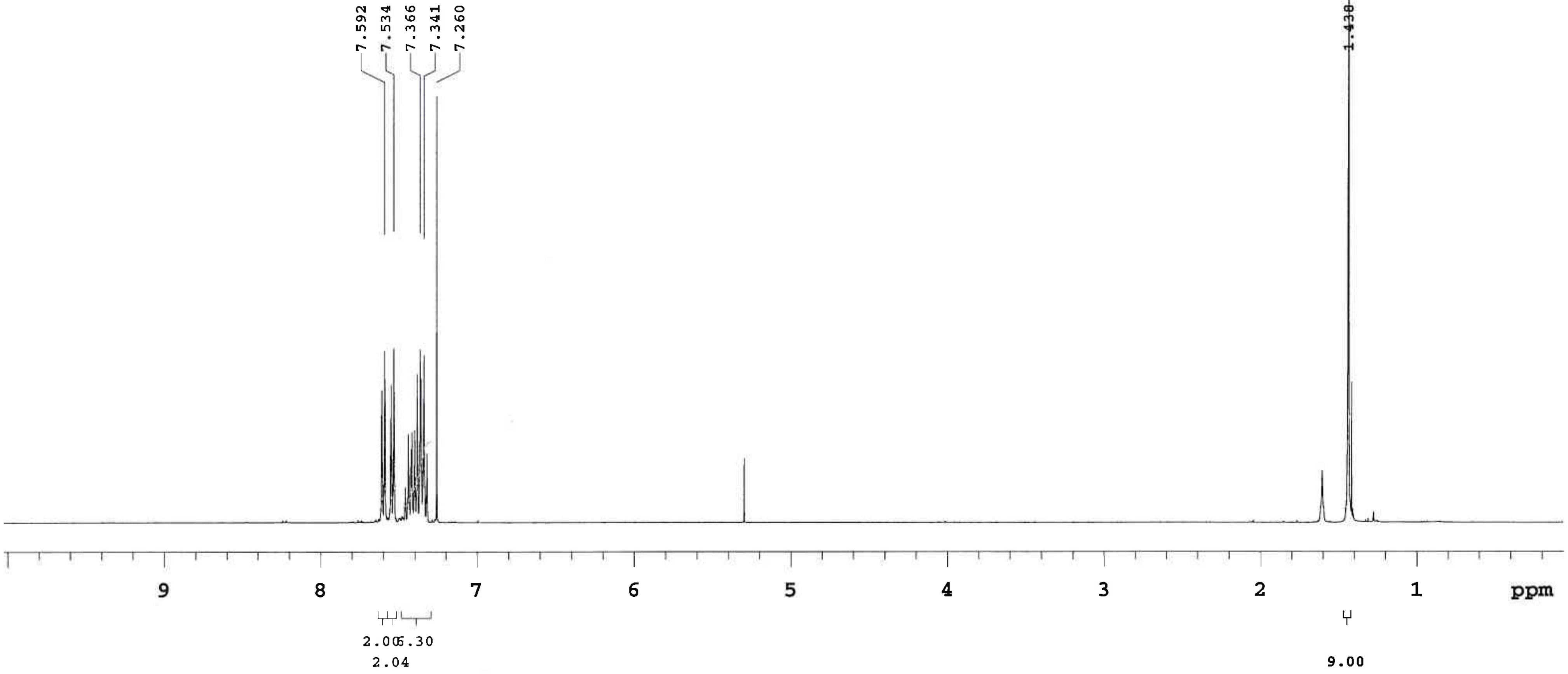

PULSE SEQUENCE
Relax. delay 0.500 sec
Pulse 48.6 degrees
Acq. time 4.797 sec
Width 6793.5 Hz
40 repetitions

OBSERVE H1, 399.6136844

DATA PROCESSING
FT size 131072
Total time 3 minutes

MS9
in CDCl3

Sample Name:
MS9
Data Collected on:
400MR-vnmrs400
Archive directory: [illegible]

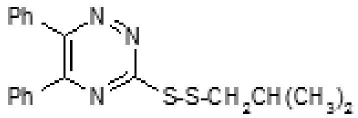

4c

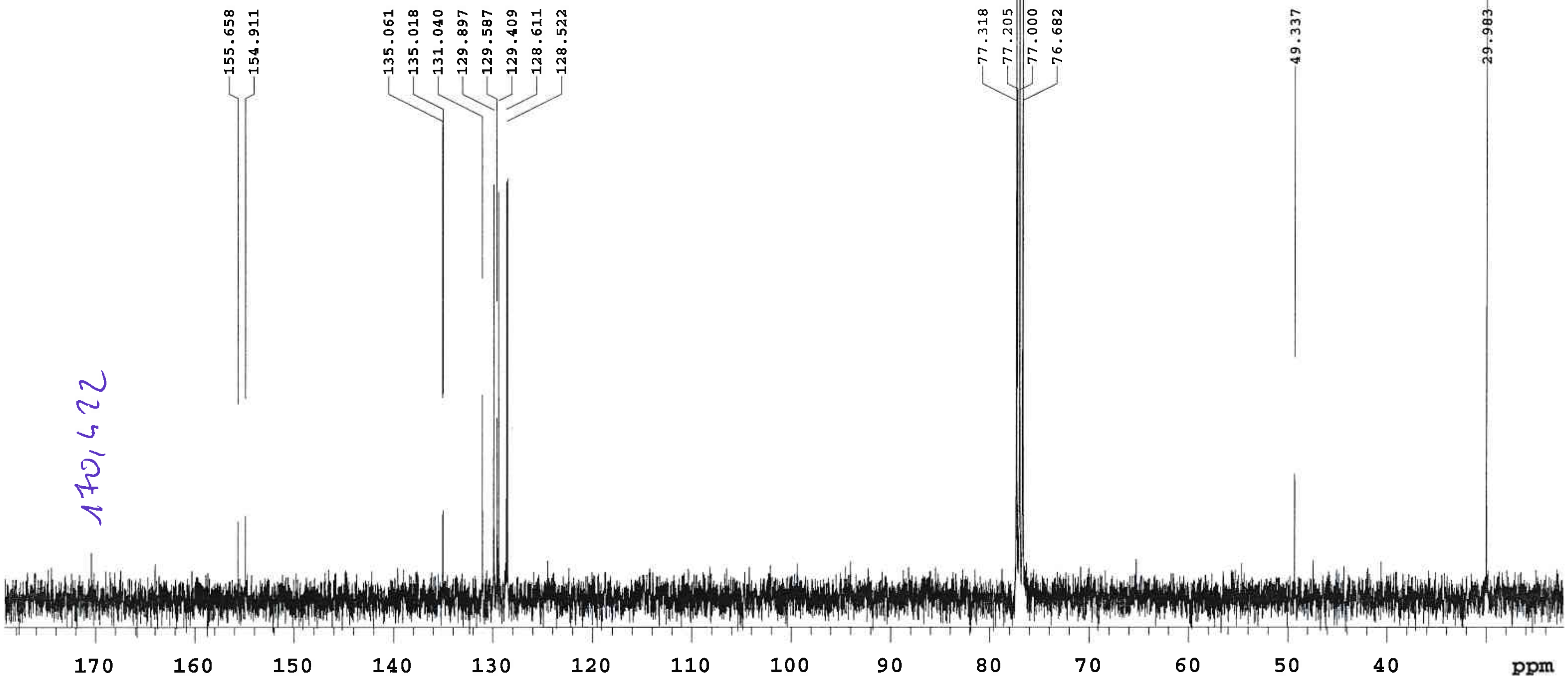

PULSE SEQUENCE
Relax. delay 1.500 sec
Pulse 38.5 degrees
Acq. time 2.000 sec
Width 25510.2 Hz
928 repetitions

OBSERVE C13, 100.4829418
DECOUPLE H1, 399.6156840
Power 36 dB
continuously on
WALTZ-16 modulated

DATA PROCESSING
Line broadening 1.0 Hz
FT size 131072
Total time 54 minutes

MS9
w CDCl3

Sample Name:
MS9
Data Collected on:
400MR-vnmrs400
Archive directory: [illegible]

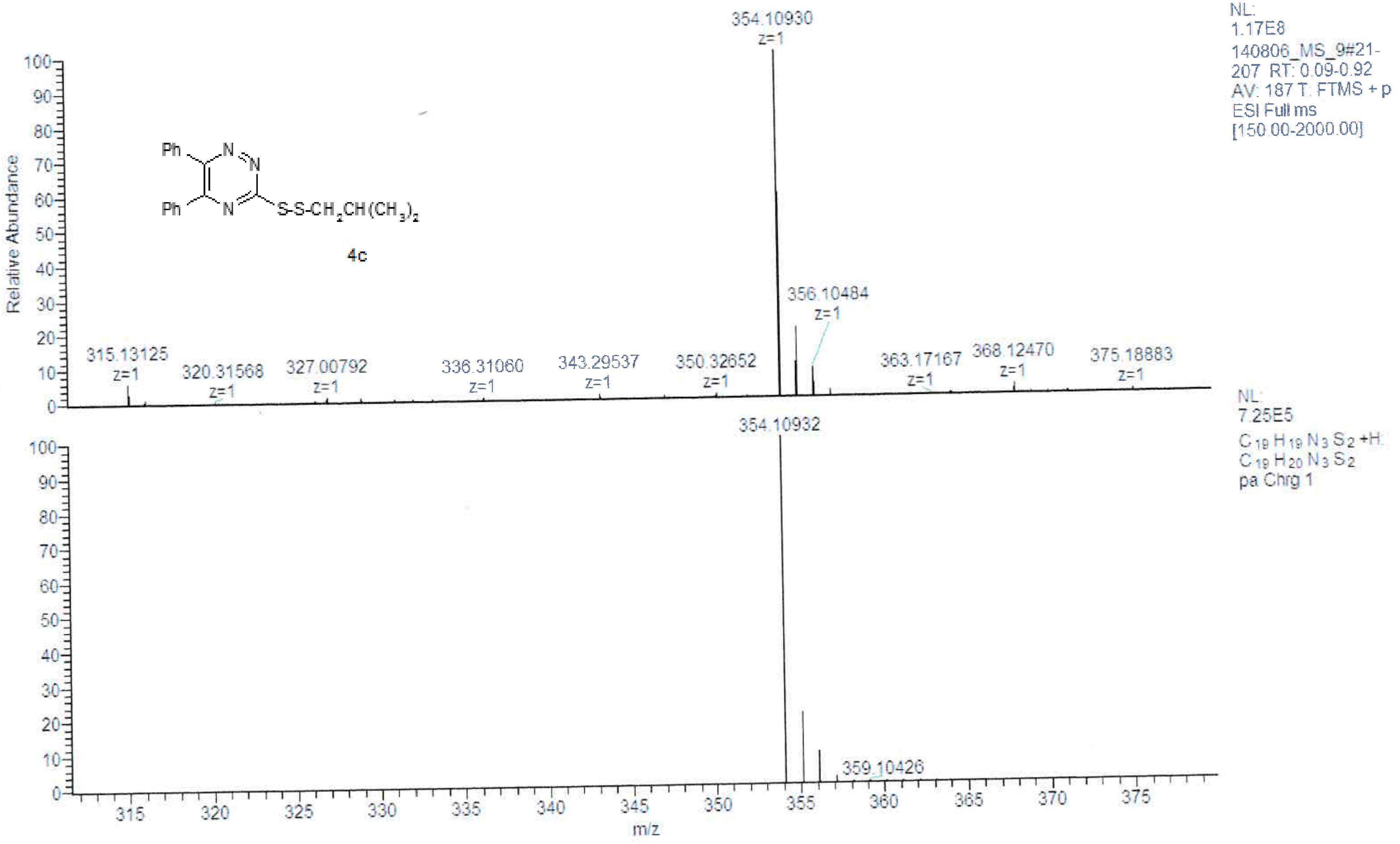
354.10930
z=1
NL:
1.17E8
140806_MS_9#21-207 RT: 0.09-0.92
AV: 187 T: FTMS + p
ESI Full ms
[150.00-2000.00]
Relative Abundance
Ph
Ph
N
N
N
S-S-CH2CH(CH3)2
4c
356.10484
z=1
315.13125
z=1
320.31568
z=1
327.00792
z=1
336.31060
z=1
343.29537
z=1
350.32652
z=1
363.17167
z=1
368.12470
z=1
375.18883
z=1
NL:
7.25E5
C19 H19 N3 S2 +H:
C19 H20 N3 S2
pa Chrg 1
354.10932
359.10426
m/z

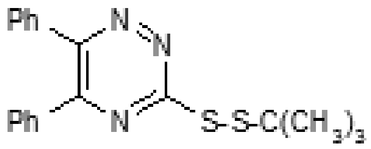

4d

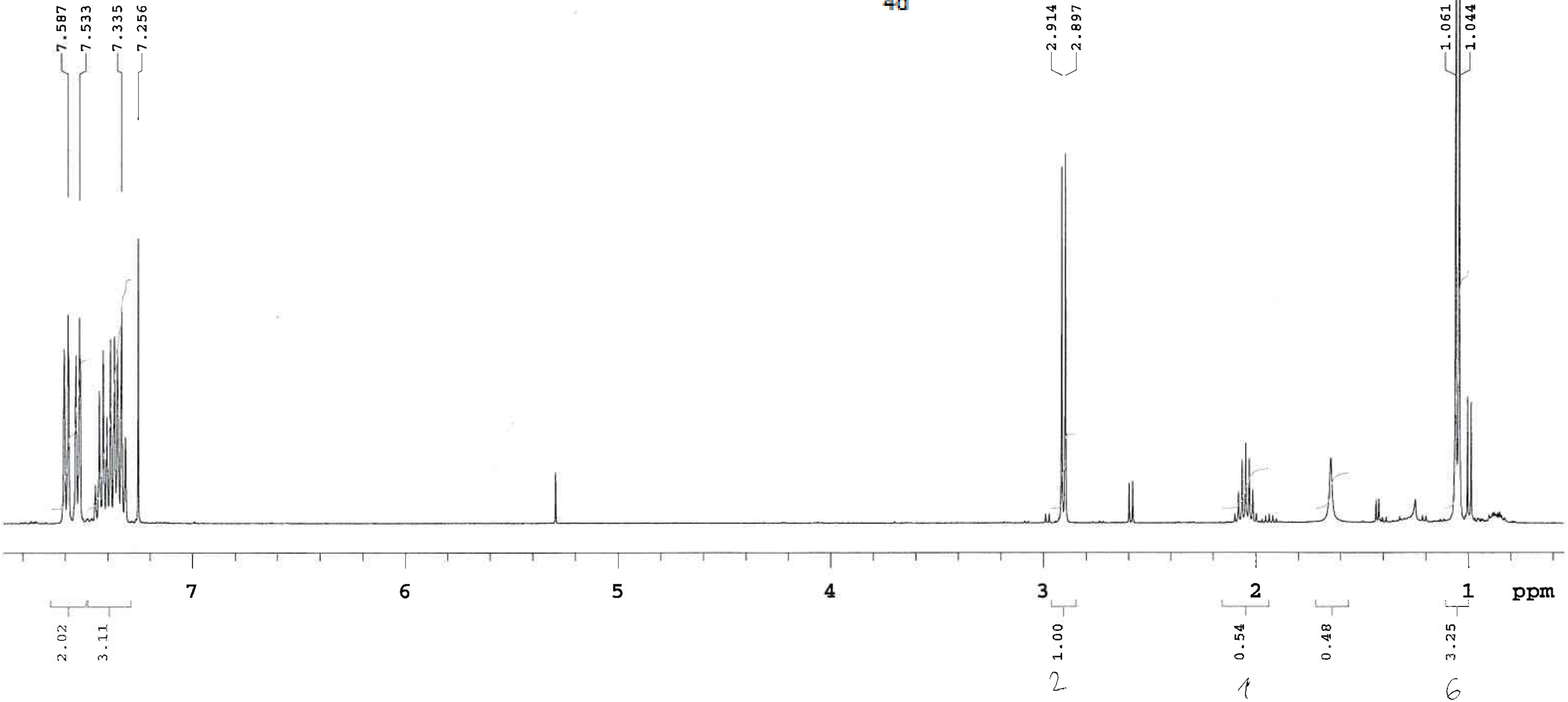

PULSE SEQUENCE
Relax. delay 0.500 sec
Pulse 48.6 degrees
Acq. time 4.797 sec
Width 6793.5 Hz
24 repetitions

OBSERVE H1, 399.6136859

DATA PROCESSING
FT size 131072
Total time 2 minutes

MS3

Sample Name:
MS3
Data Collected on:
400MR-vnmrs400
Archive directory:
[illegible]

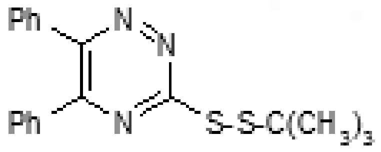

Ph
N
N
Ph
N
S-S-C(CH3)3

4d

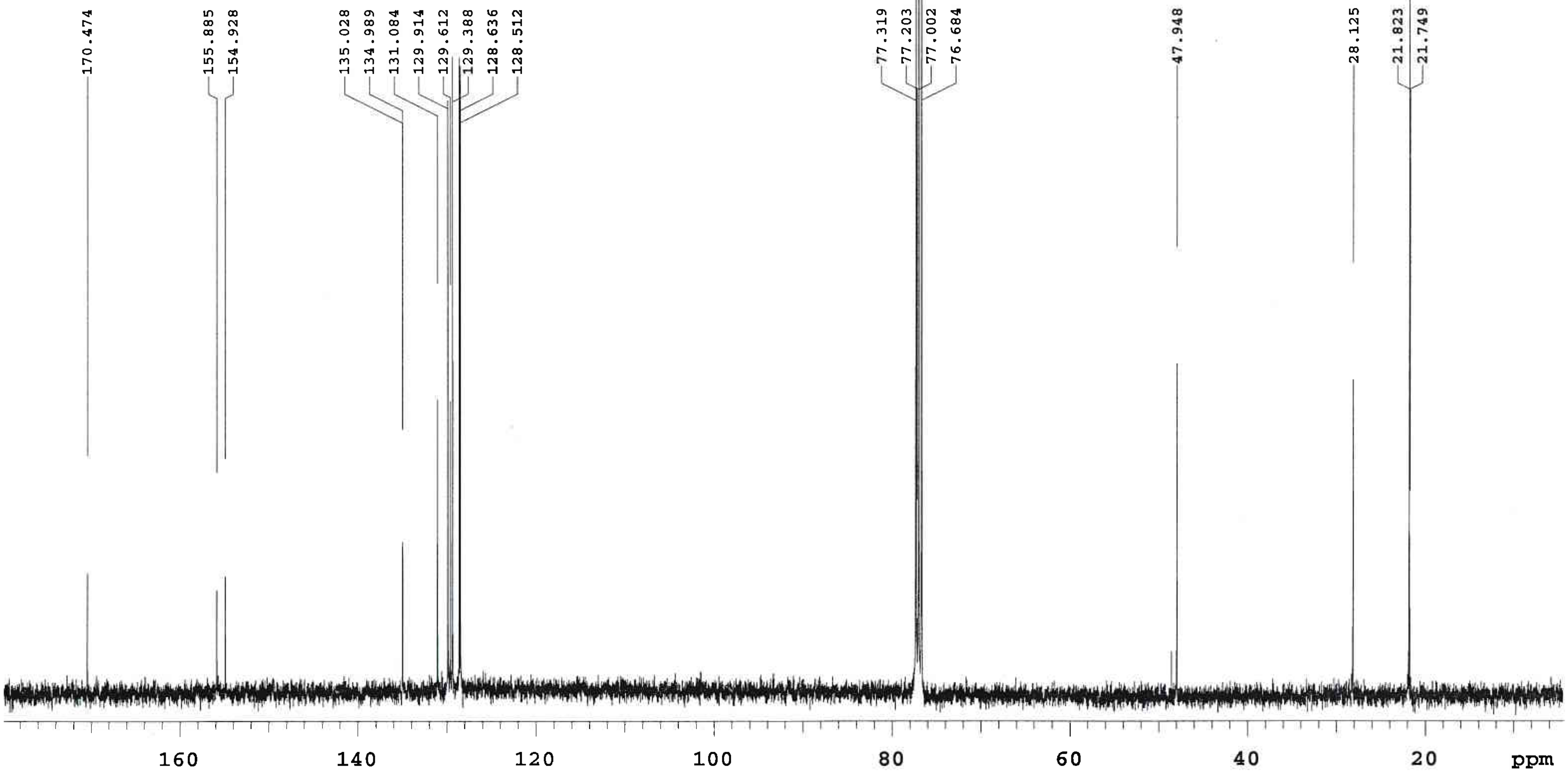

170.474
155.885
154.928
135.028
134.989
131.084
129.914
129.612
129.388
128.636
128.512
77.319
77.203
77.002
76.684
47.948
28.125
21.823
21.749
160
140
120
100
80
60
40
20
ppm

MS3

$CDCl_3$

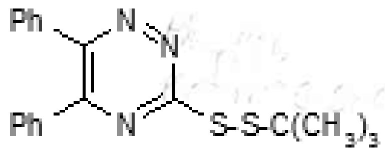

Ph
N
N
Ph
N
S-S-C(CH3)3

4d

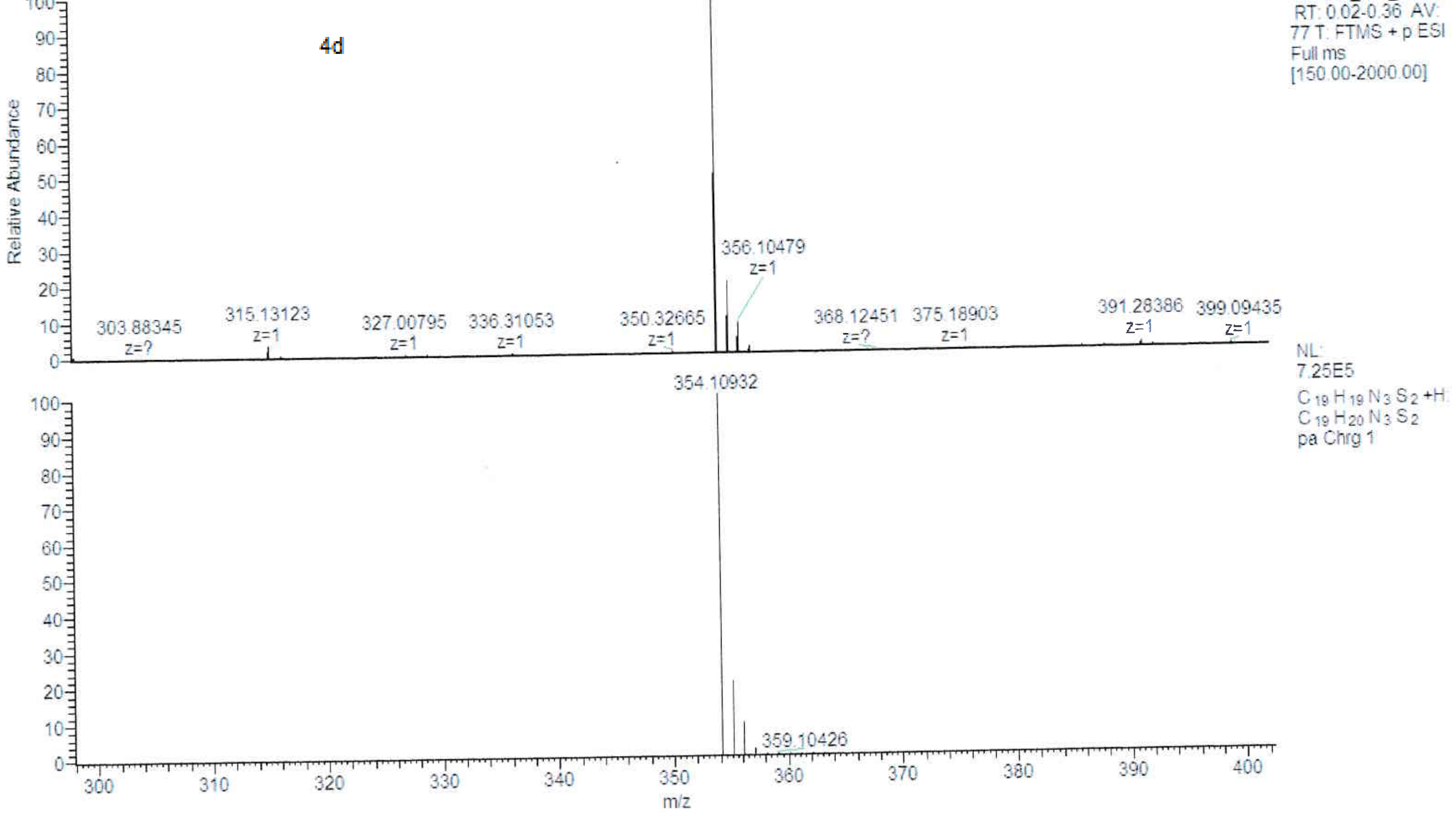

354.10934
z=1
NL:
2.35E8
140806_MS_3#5-81
RT: 0.02-0.36 AV:
77 T: FTMS + p ESI
Full ms
[150.00-2000.00]
Relative Abundance
356.10479
z=1
303.88345
z=?
315.13123
z=1
327.00795
z=1
336.31053
z=1
350.32665
z=1
368.12451
z=?
375.18903
z=1
391.28386
z=1
399.09435
z=1
NL:
7.25E5
C 19 H 19 N 3 S 2 +H:
C 19 H 20 N 3 S 2
pa Chrg 1
354.10932
359.10426
m/z

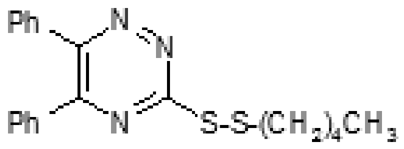

4e

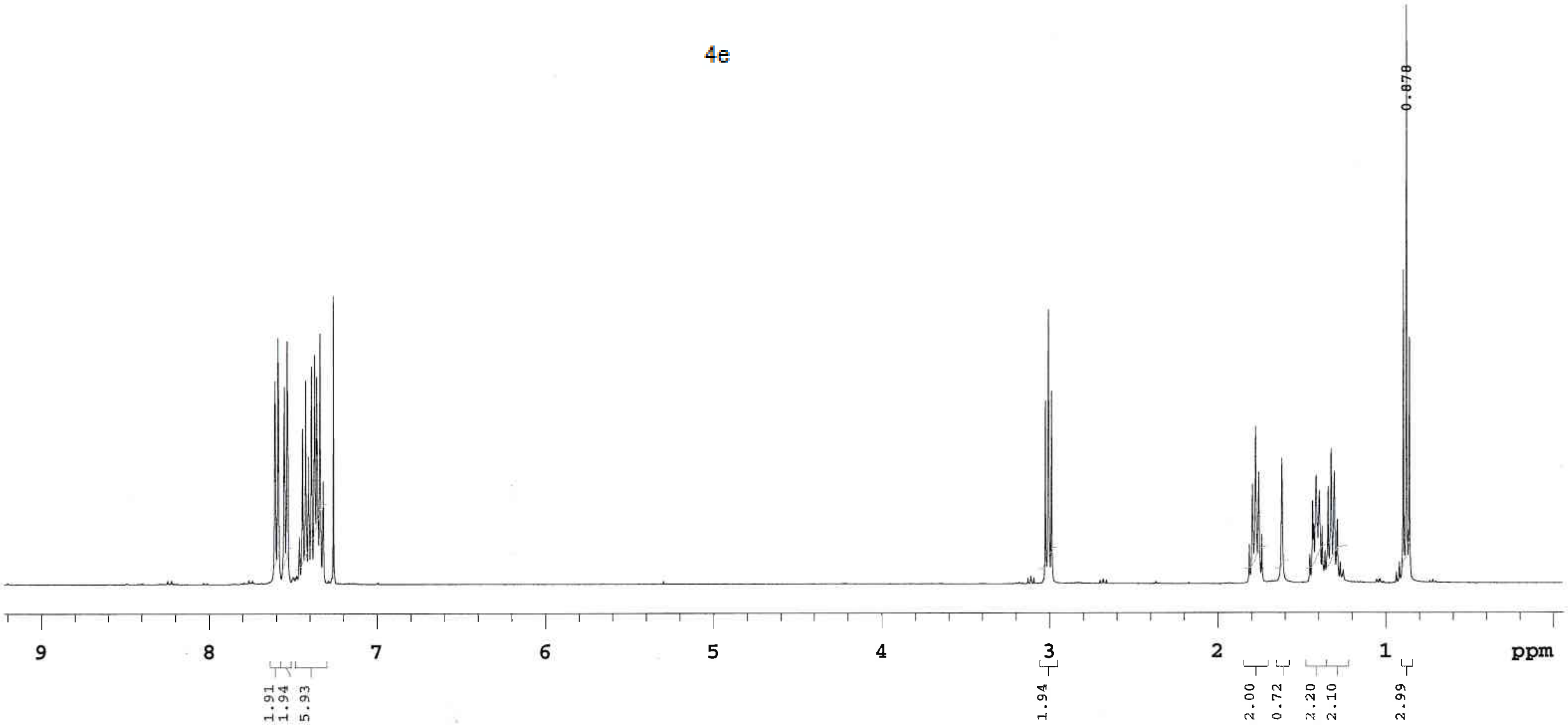

PULSE SEQUENCE
Relax. delay 0.500 sec
Pulse 48.6 degrees
Acq. time 4.797 sec
Width 6793.5 Hz
40 repetitions

OBSERVE H1, 399.6136844

DATA PROCESSING
FT size 131072
Total time 3 minutes

MS8
in CDCl3

Sample Name:
MS8
Data Collected on:
400MR-vnmrs400
Archive directory: [illegible]

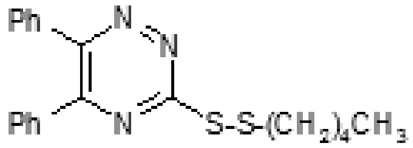

4e

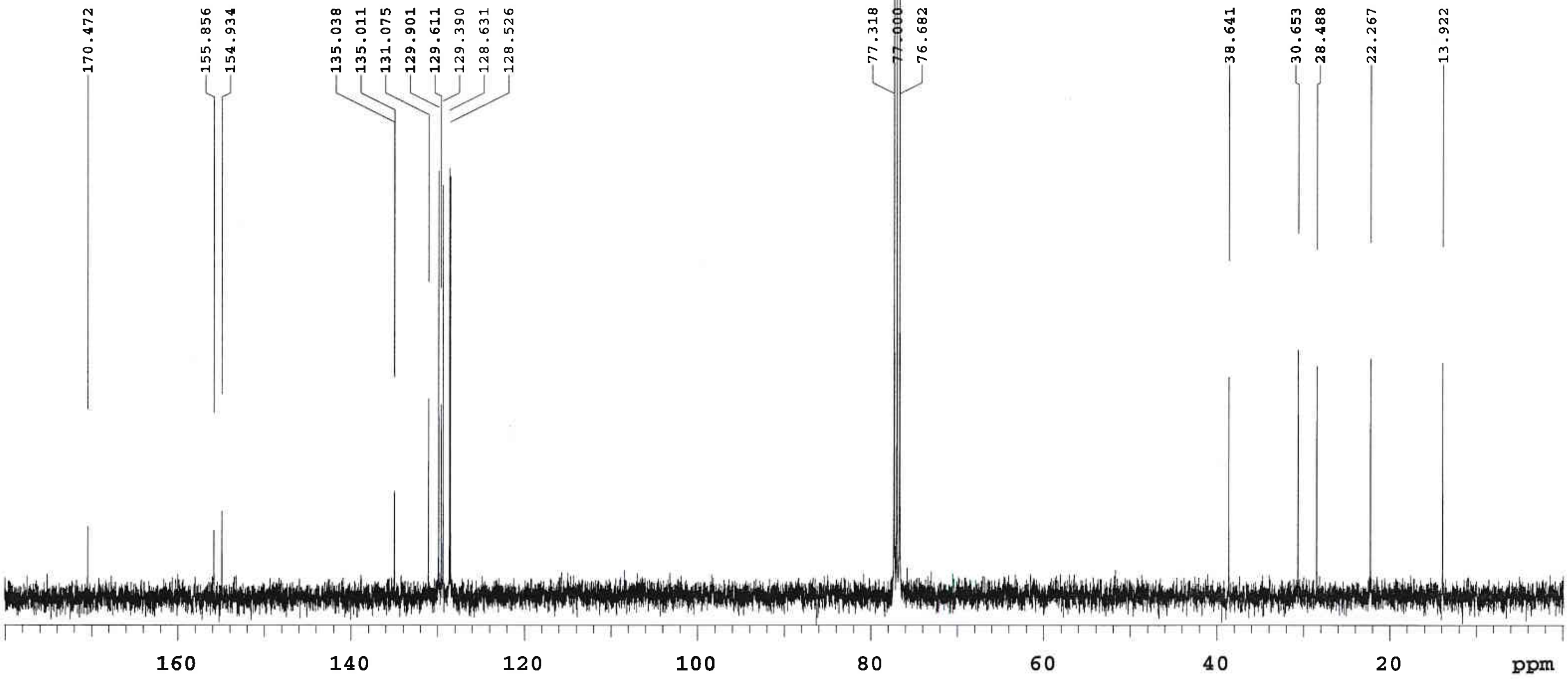

PULSE SEQUENCE
Relax. delay 1.500 sec
Pulse 38.5 degrees
Acq. time 2.000 sec
Width 25510.2 Hz
768 repetitions

OBSERVE C13, 100.4829422
DECOUPLE H1, 399.6156840
Power 36 dB
continuously on
WALTZ-16 modulated

DATA PROCESSING
Line broadening 1.0 Hz
FT size 131072
Total time 44 minutes

MS8
w CDCl3

Sample Name:
MS8
Data Collected on:
400MR-vnmrs400
Archive directory: [illegible]

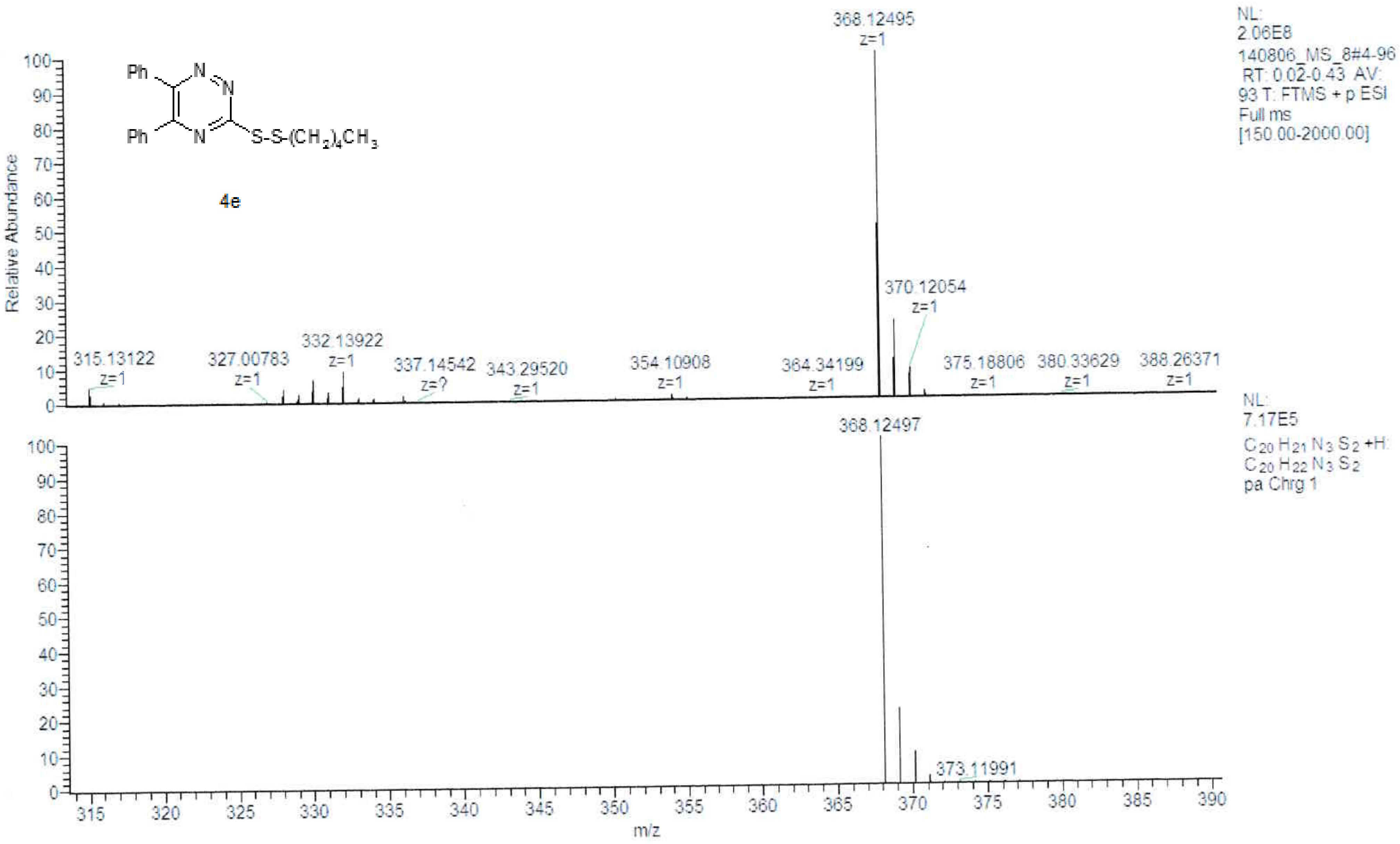

Ph
N
N
Ph
N
S-S-(CH2)4CH3
4e
Relative Abundance
368.12495
z=1
370.12054
z=1
315.13122
z=1
327.00783
z=1
332.13922
z=1
337.14542
z=?
343.29520
z=1
354.10908
z=1
364.34199
z=1
375.18806
z=1
380.33629
z=1
388.26371
z=1
NL:
2.06E8
140806_MS_8#4-96
RT: 0.02-0.43 AV:
93 T: FTMS + p ESI
Full ms
[150.00-2000.00]
368.12497
373.11991
NL:
7.17E5
C20 H21 N3 S2 +H:
C20 H22 N3 S2
pa Chrg 1
m/z

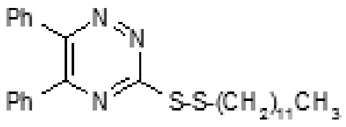

4f

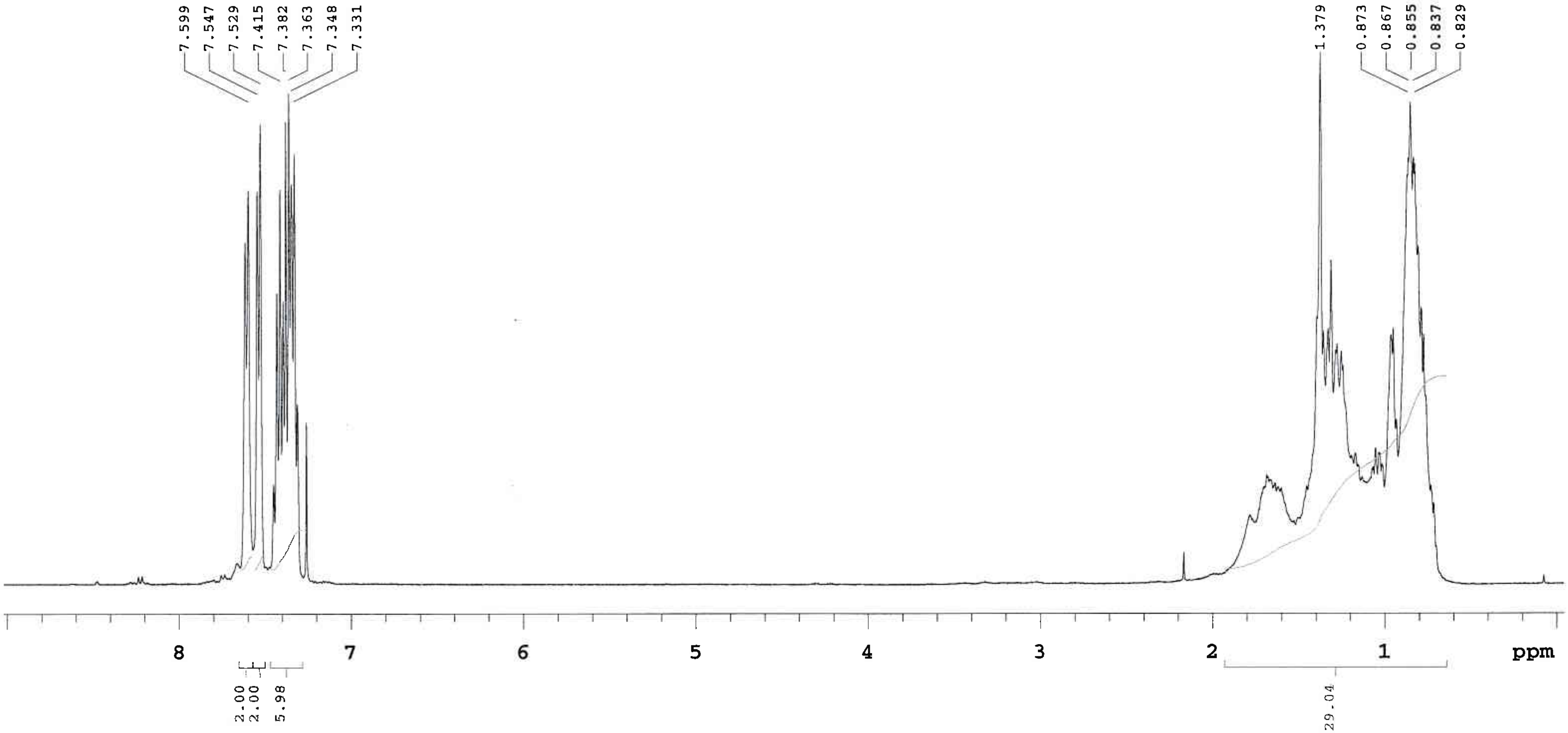

ZIBA 9

21BA9
in CDCl3

PULSE SEQUENCE
Relax. delay 0.500 sec
Pulse 48.6 degrees
Acq. time 4.797 sec
Width 6793.5 Hz
40 repetitions

OBSERVE H1, 399.6136843

DATA PROCESSING
FT size 65536
Total time 3 minutes

Sample Name:
21BA9
Data Collected on:
400MR-vnmrs400
Archive directory: [illegible] 2014

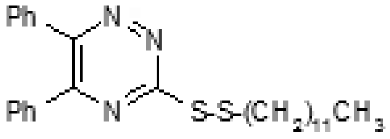

4f

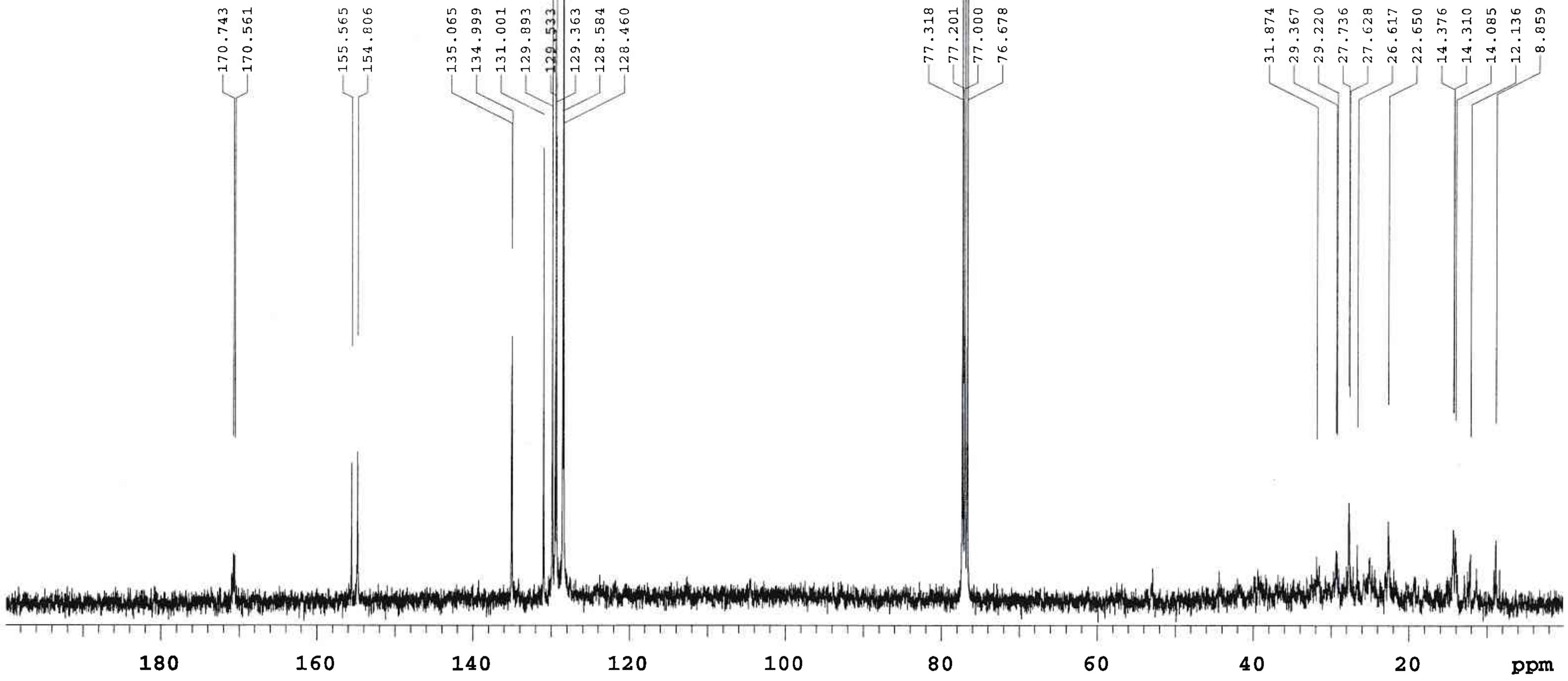

PULSE SEQUENCE
Relax. delay 1.500 sec
Pulse 38.5 degrees
Acq. time 2.000 sec
Width 25510.2 Hz
4192 repetitions

OBSERVE C13, 100.4829453
DECOUPLE H1, 399.6156840
Power 36 dB
continuously on
WALTZ-16 modulated

DATA PROCESSING
Line broadening 1.5 Hz
FT size 131072
Total time 4.1 hours

ZIBA9 w CDC13

Sample Name:
ZIBA9
Data Collected on:
400MR-vnmrs400
Archive directory:
[illegible]

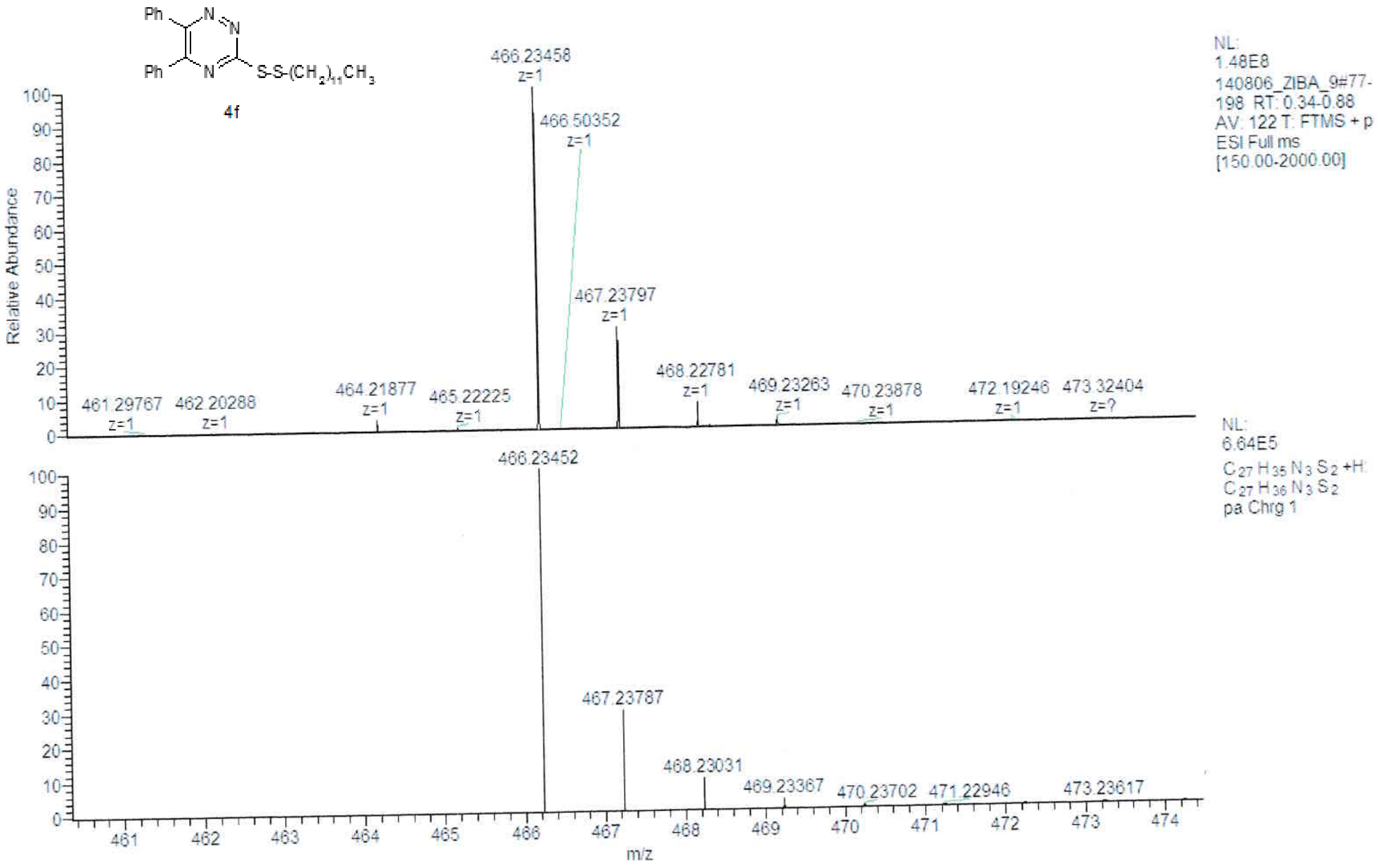
Ph
Ph
N
N
N
S-S-(CH2)11CH3
4f
466.23458
z=1
466.50352
z=1
467.23797
z=1
468.22781
z=1
469.23263
z=1
470.23878
z=1
472.19246
z=1
473.32404
z=?
461.29767
z=1
462.20288
z=1
464.21877
z=1
465.22225
z=1
Relative Abundance
NL:
1.48E8
140806_ZIBA_9#77-198 RT: 0.34-0.88
AV: 122 T: FTMS + p ESI Full ms [150.00-2000.00]
466.23452
467.23787
468.23031
469.23367
470.23702
471.22946
473.23617
NL:
6.64E5
C27 H35 N3 S2 +H:
C27 H36 N3 S2
pa Chrg 1
m/z

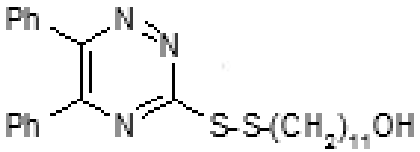

4g

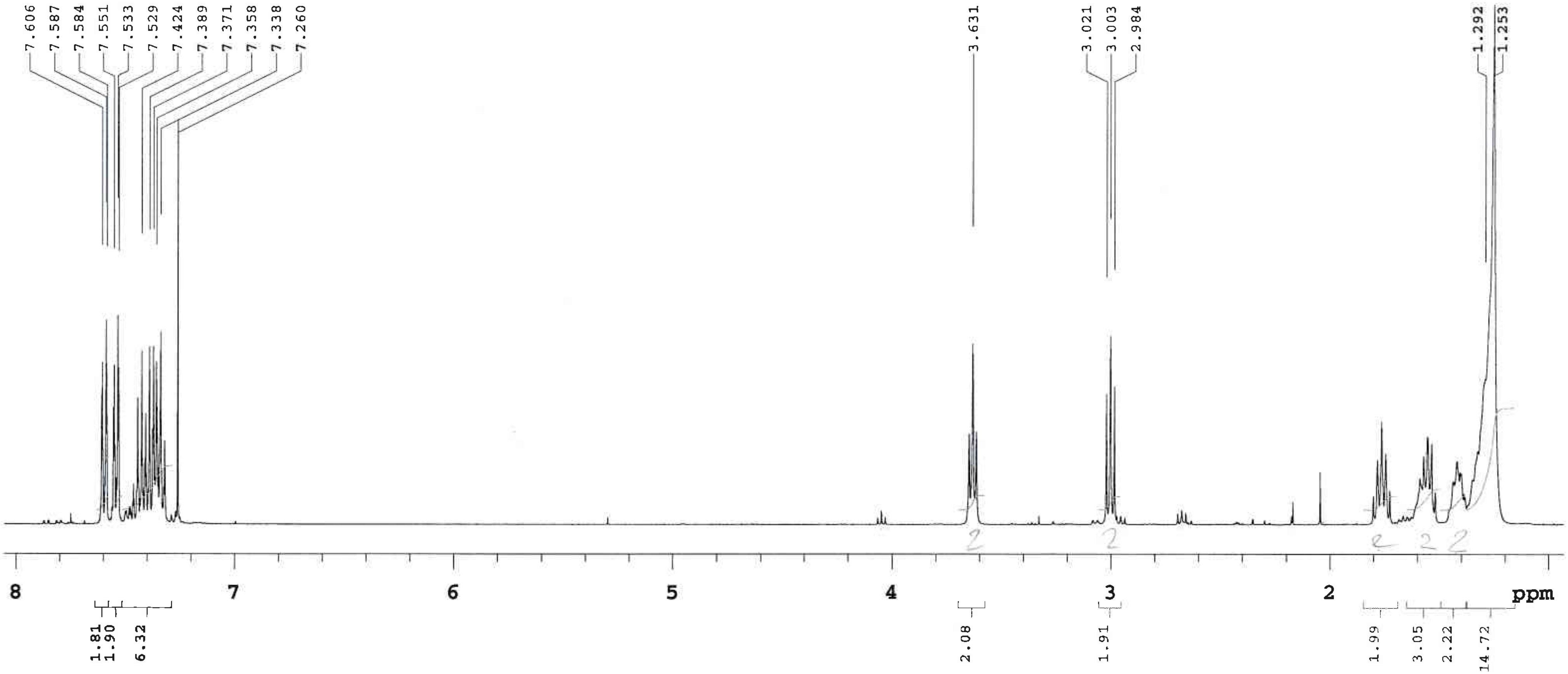

PULSE SEQUENCE
Relax. delay 0.500 sec
Pulse 48.6 degrees
Acq. time 4.797 sec
Width 6793.5 Hz
60 repetitions

OBSERVE H1, 399.6053329

DATA PROCESSING
FT size 65536
Total time 5 minutes

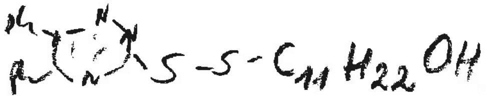

Mn30b
in CDCl3

Sample Name:
Mn30b
Data Collected on:
[illegible]

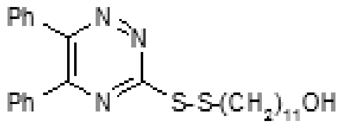

4g

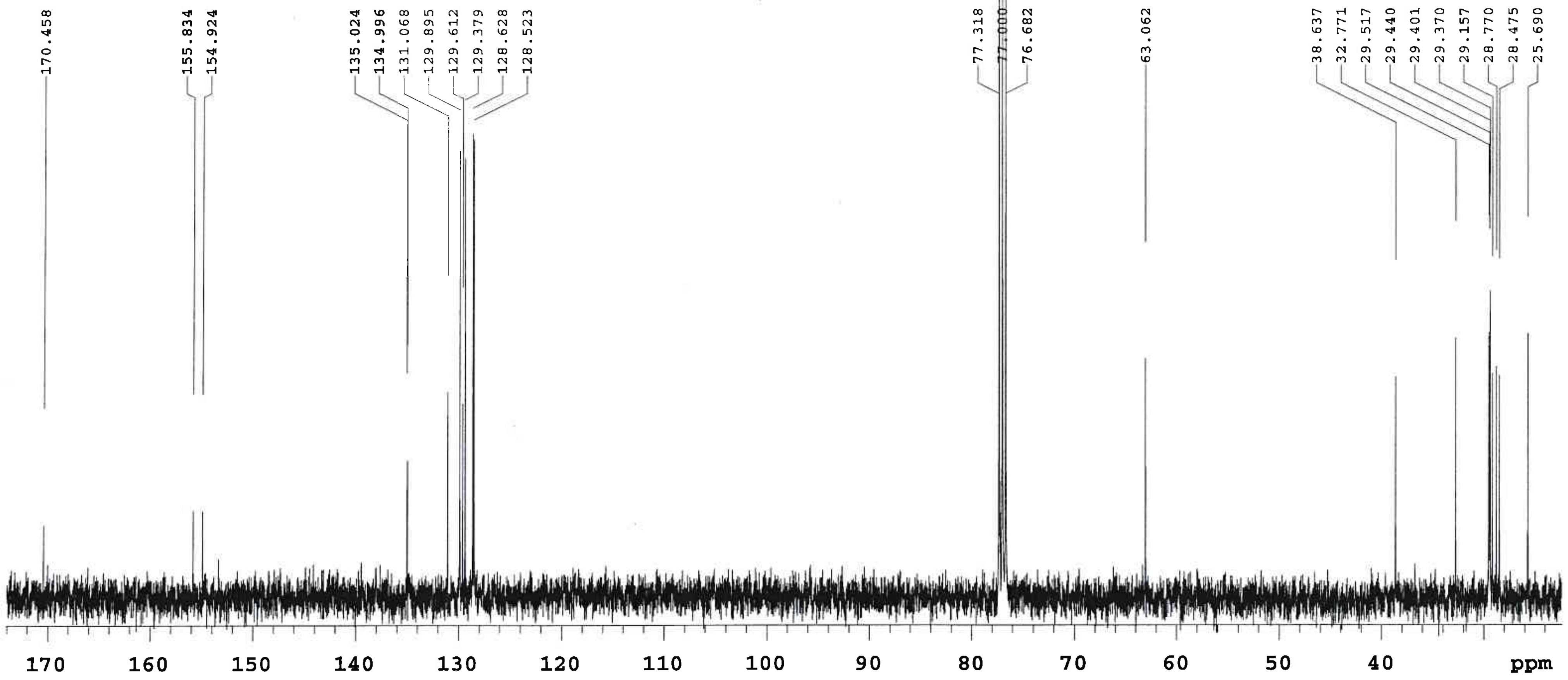

PULSE SEQUENCE
Relax. delay 1.500 sec
Pulse 38.5 degrees
Acq. time 2.000 sec
Width 25510.2 Hz
928 repetitions

OBSERVE C13, 100.4808429
DECOUPLE H1, 399.6073319
Power 36 dB
continuously on
WALTZ-16 modulated

DATA PROCESSING
Line broadening 1.0 Hz
FT size 131072
Total time 54 minutes

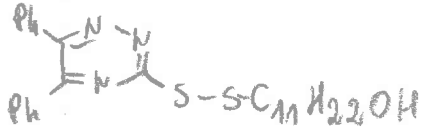

Mn30b
in CDCl3

Sample Name:
Mn30b
Data Collected on:
[illegible]

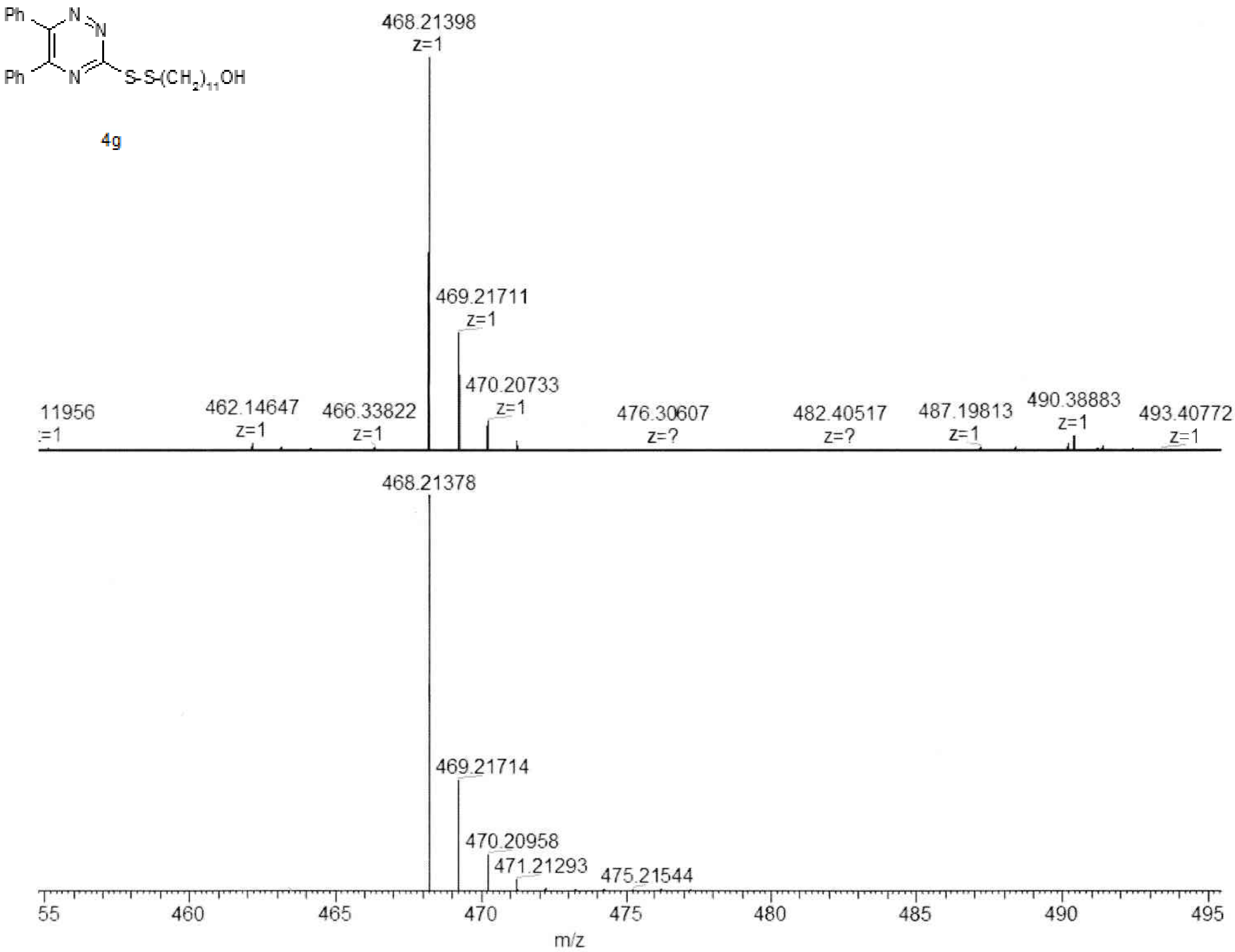
Ph
N
N
Ph
N
S-S-(CH2)11OH
4g
468.21398
z=1
469.21711
z=1
470.20733
z=1
11956
=1
462.14647
z=1
466.33822
z=1
476.30607
z=?
482.40517
z=?
487.19813
z=1
490.38883
z=1
493.40772
z=1
468.21378
469.21714
470.20958
471.21293
475.21544
55
460
465
470
475
480
485
490
495
m/z

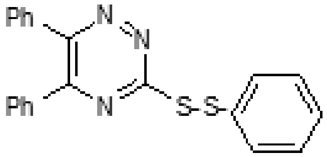

4h

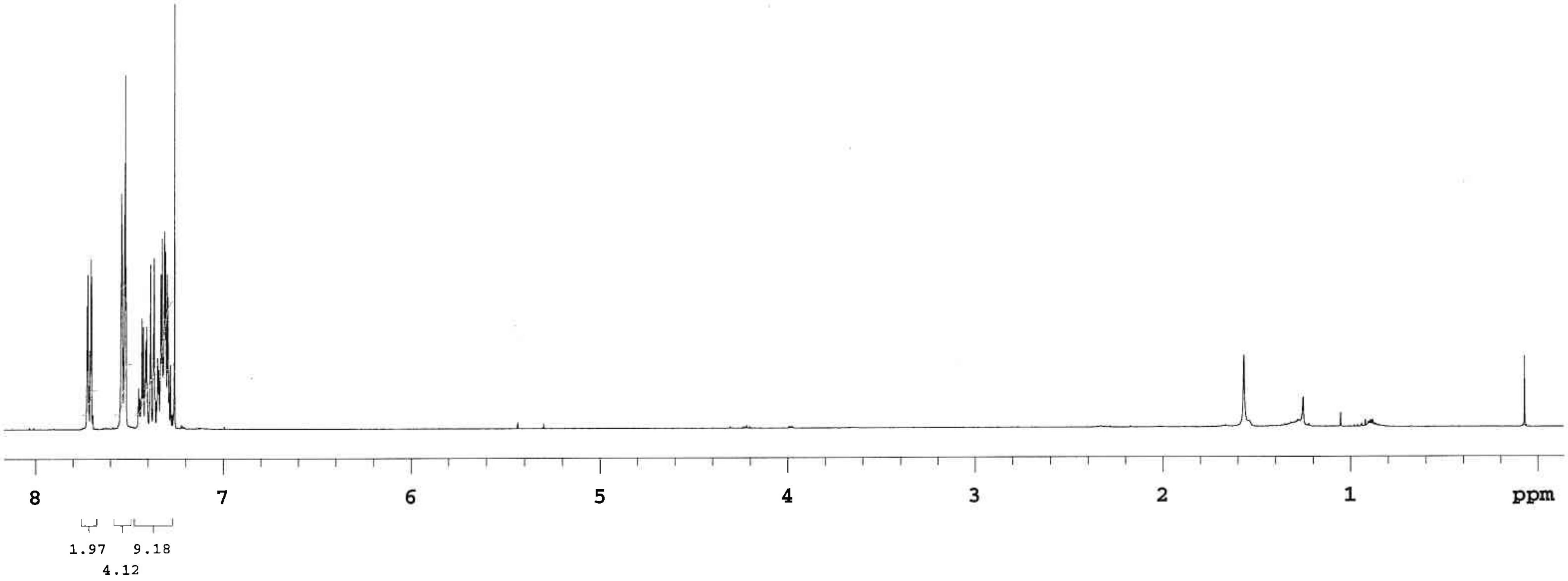

PULSE SEQUENCE
Relax. delay 0.500 sec
Pulse 48.6 degrees
Acq. time 4.797 sec
Width 6793.5 Hz
28 repetitions

OBSERVE H1, 399.6227914

DATA PROCESSING
FT size 131072
Total time 2 minutes

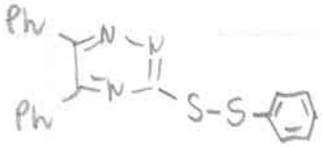

OSk20
in CDCl3

Sample Name:
OSk20
Data Collected on:
400MR-vnmrs400
Archive directory: [illegible]

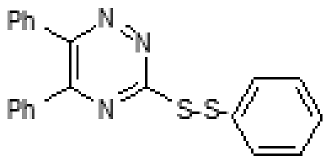

4h

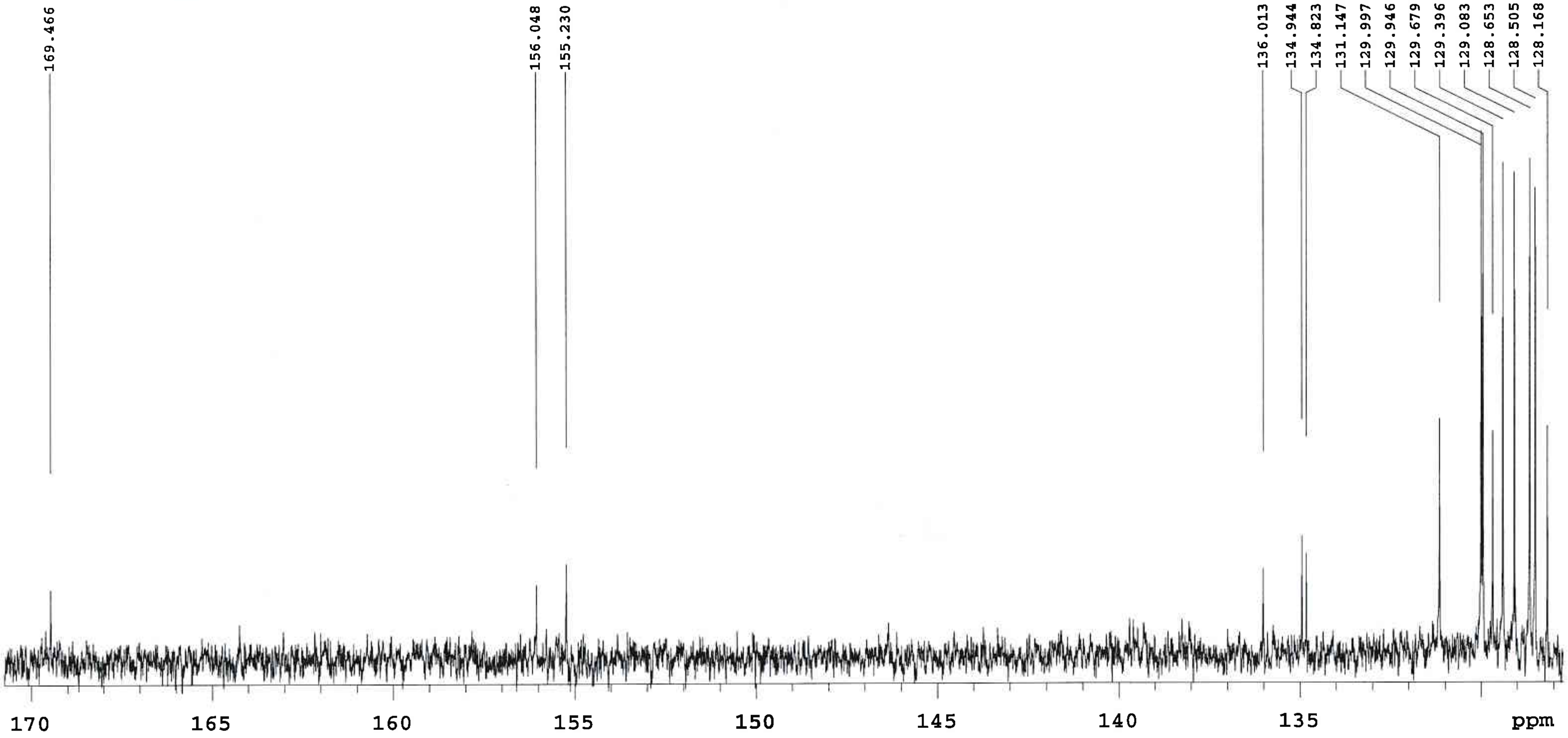

PULSE SEQUENCE
Relax. delay 1.500 sec
Pulse 38.5 degrees
Acq. time 2.000 sec
Width 25510.2 Hz
3120 repetitions

OBSERVE C13, 100.4852320
DECOUPLE H1, 399.6247963
Power 36 dB
continuously on
WALTZ-16 modulated

DATA PROCESSING
Line broadening 1.0 Hz
FT size 131072
Total time 3.0 hours

OSk13
in CDCl3

Sample Name:
OSk13
Data Collected on:
400MR-vnmrs400
Archive directory: [illegible]

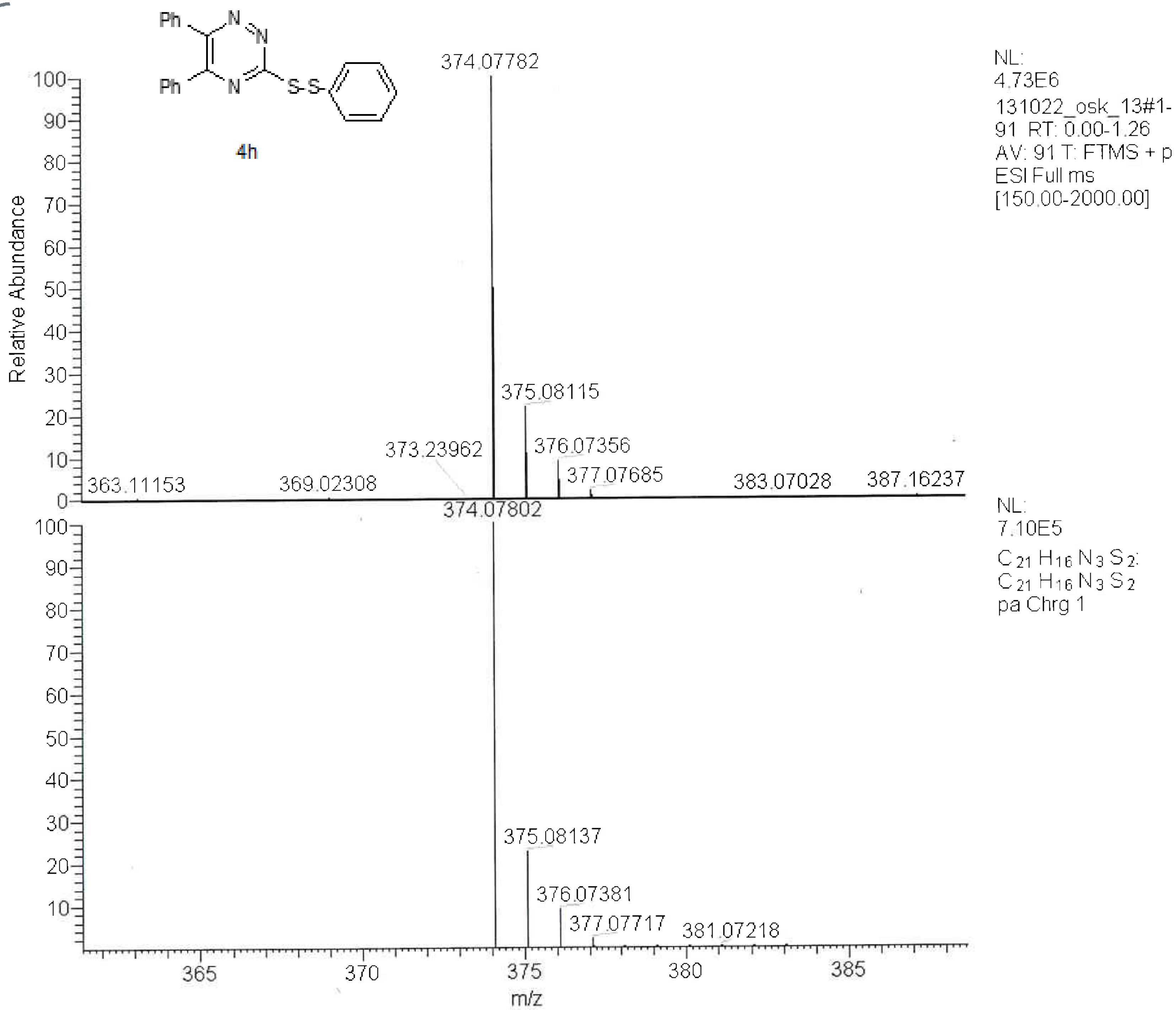

Ph
Ph
N
N
N
S-S
4h
374.07782
375.08115
376.07356
377.07685
373.23962
363.11153
369.02308
383.07028
387.16237
374.07802
375.08137
376.07381
377.07717
381.07218
Relative Abundance
m/z
NL:
4.73E6
131022_osk_13#1-91 RT: 0.00-1.26 AV: 91 T: FTMS + p ESI Full ms [150.00-2000.00]
NL:
7.10E5
$C_{21}H_{16}N_3S_2$:
$C_{21}H_{16}N_3S_2$
pa Chrg 1

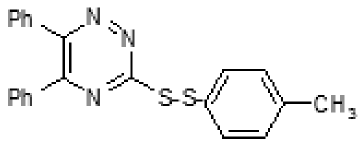

4i

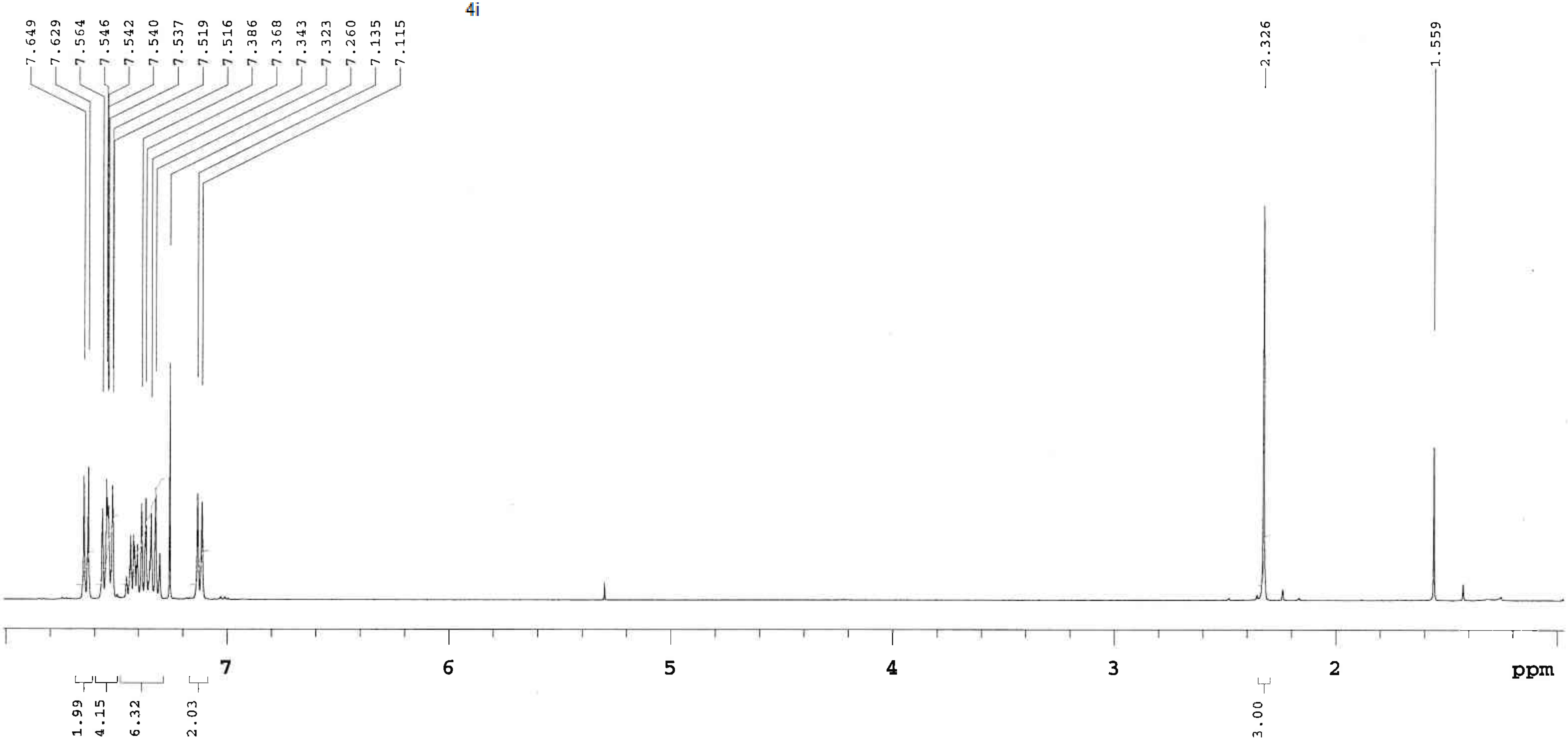

PULSE SEQUENCE
Relax. delay 0.500 sec
Pulse 48.6 degrees
Acq. time 4.797 sec
Width 6793.5 Hz
40 repetitions

OBSERVE H1, 399.6053329

DATA PROCESSING
FT size 65536
Total time 3 minutes

MN29 in CDC13

Sample Name:
MN29
Data Collected on:
400MR-vnmrs400
Archive directory:
[illegible]

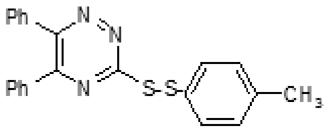

4i

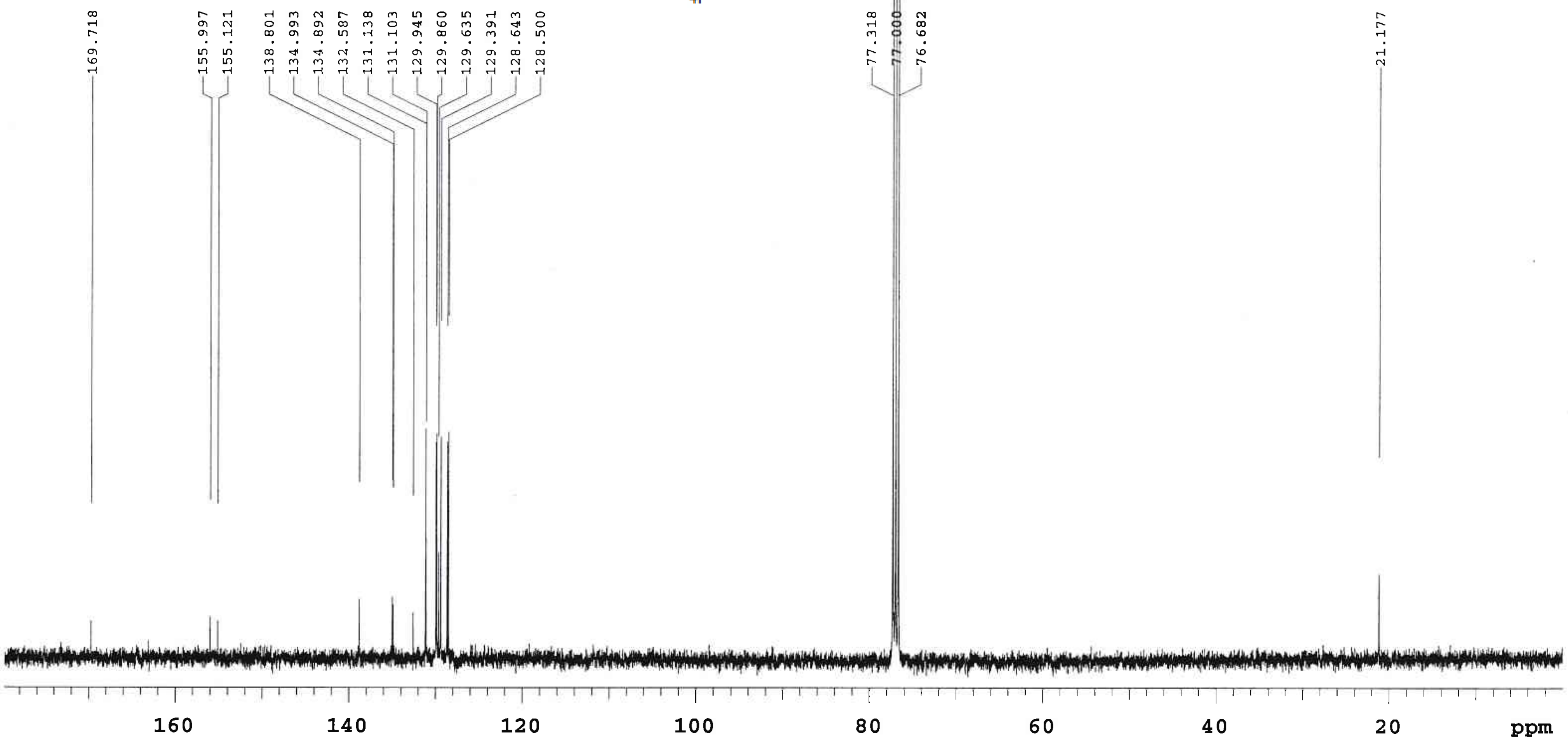

PULSE SEQUENCE
Relax. delay 1.500 sec
Pulse 38.5 degrees
Acq. time 2.000 sec
Width 25510.2 Hz
3040 repetitions

OBSERVE C13, 100.4808425
DECOUPLE H1, 399.6073319
Power 36 dB
continuously on
WALTZ-16 modulated

DATA PROCESSING
Line broadening 1.0 Hz
FT size 131072
Total time 3.0 hours

MN29 w CDCl3

Sample Name:
MN29
Data Collected on:
400MR-vnmrs400
Archive directory:
[illegible]

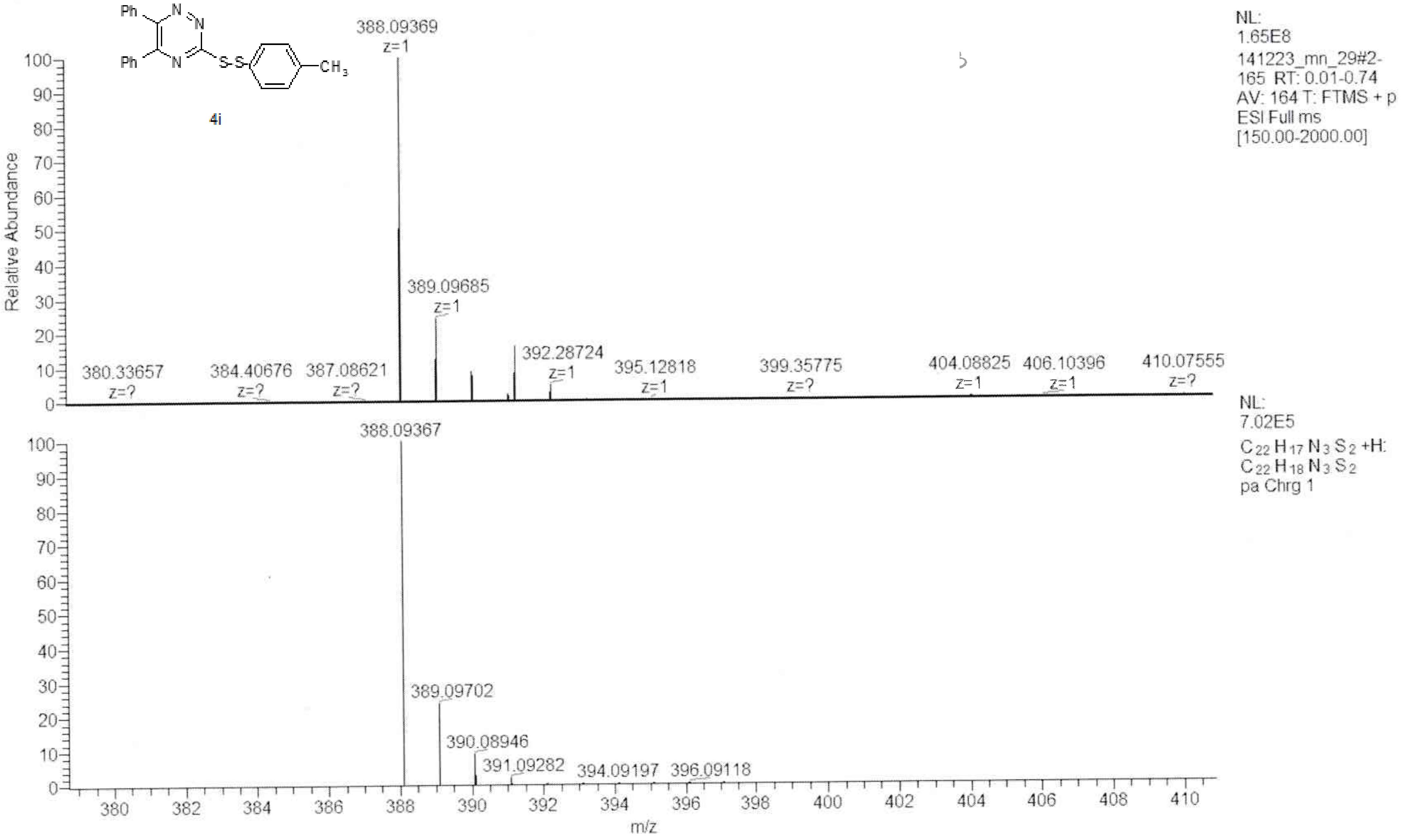

Ph
N
N
Ph
N
S-S
CH3
4i
388.09369
z=1
389.09685
z=1
392.28724
z=1
380.33657
z=?
384.40676
z=?
387.08621
z=?
395.12818
z=1
399.35775
z=?
404.08825
z=1
406.10396
z=1
410.07555
z=?
NL:
1.65E8
141223_mn_29#2-165 RT: 0.01-0.74 AV: 164 T: FTMS + p ESI Full ms [150.00-2000.00]
Relative Abundance
388.09367
389.09702
390.08946
391.09282
394.09197
396.09118
NL:
7.02E5
C22 H17 N3 S2 +H: C22 H18 N3 S2 pa Chrg 1
m/z

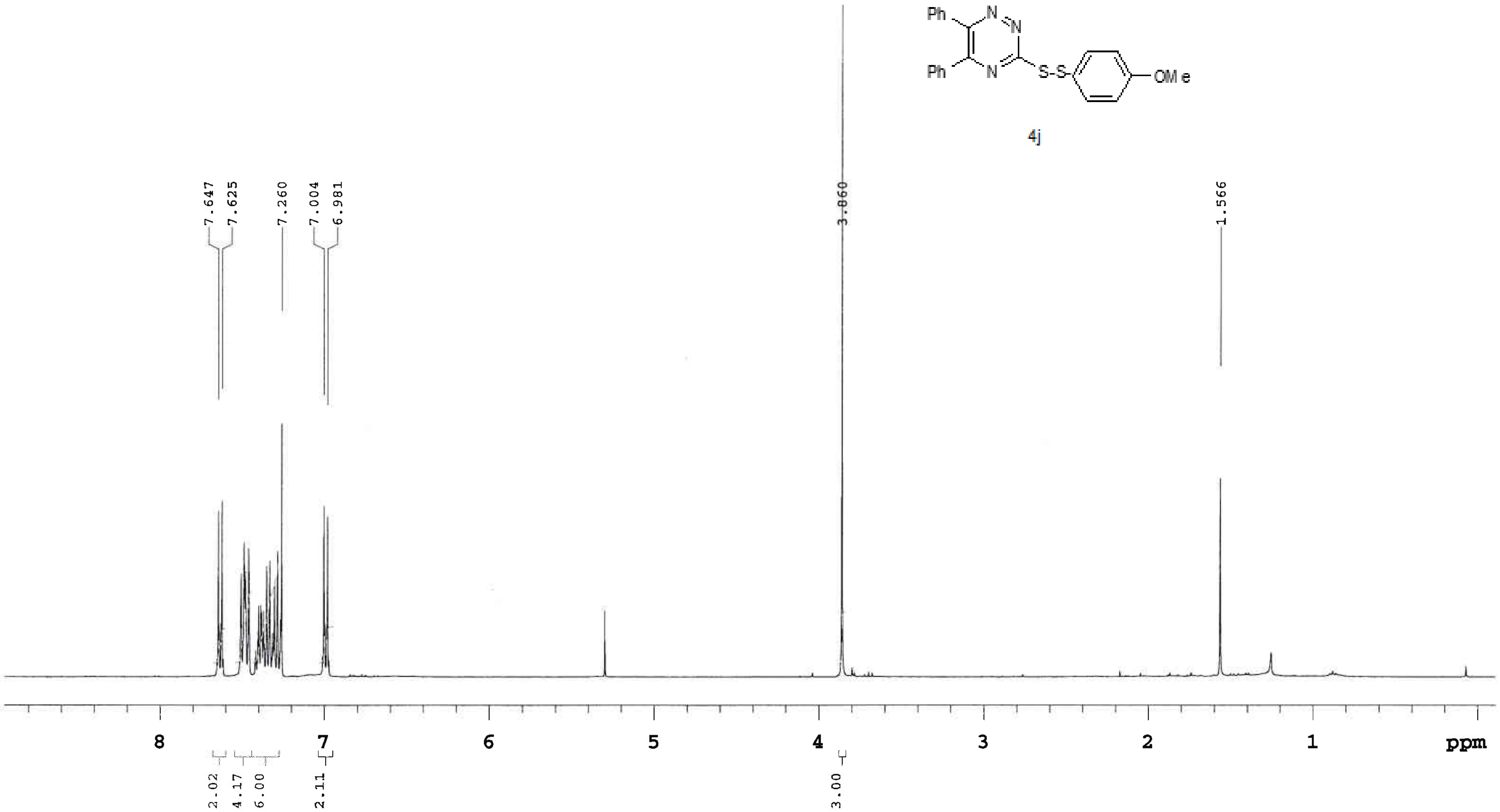

PULSE SEQUENCE
Relax. delay 0.500 sec
Pulse 48.6 degrees
Acq. time 4.797 sec
Width 6793.5 Hz
40 repetitions

OBSERVE H1, 399.6053325

DATA PROCESSING
FT size 65536
Total time 3 minutes

MN32
in CDCl3

Sample Name:
MN32
Data Collected on:
400MR-vnmrs400
Archive directory:
[illegible]

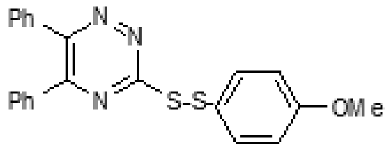

4j

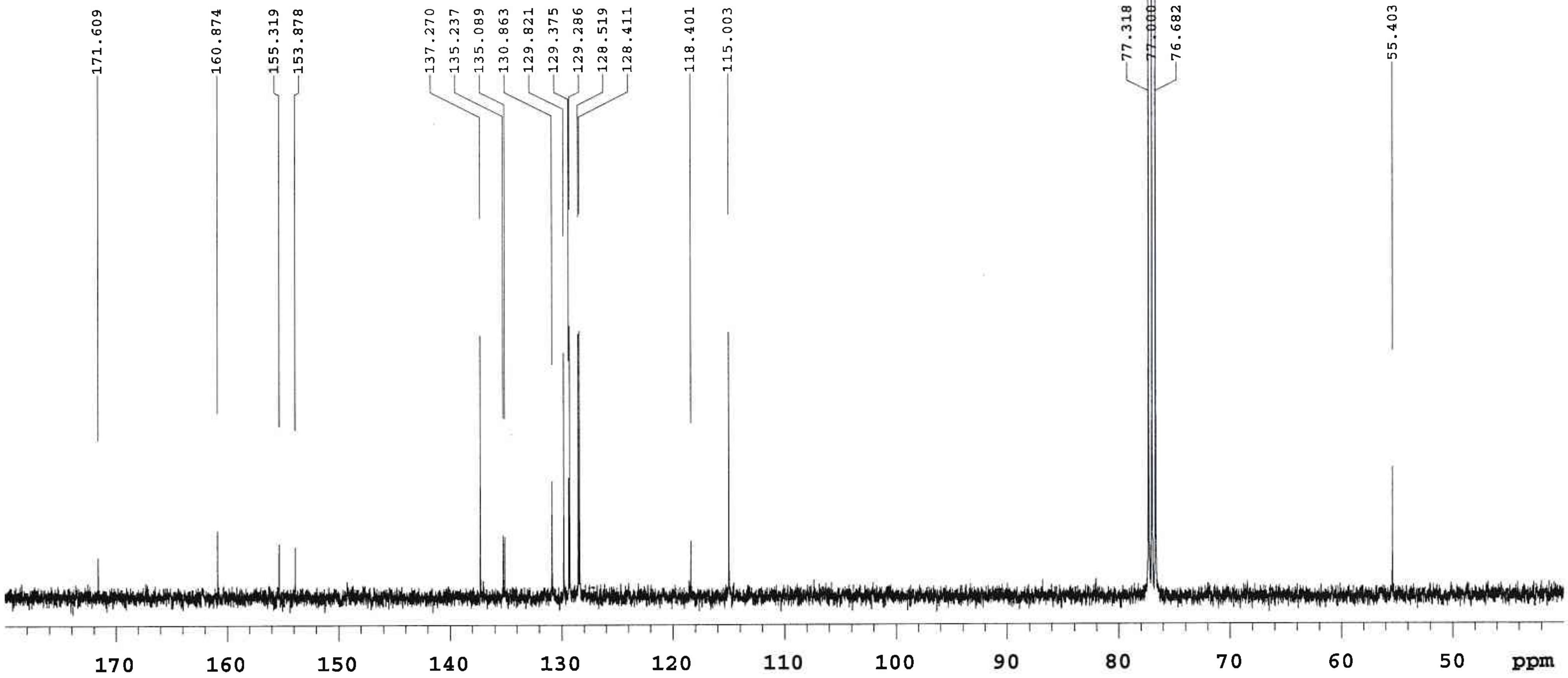

PULSE SEQUENCE
Relax. delay 1.500 sec
Pulse 38.5 degrees
Acq. time 2.000 sec
Width 25510.2 Hz
2304 repetitions

OBSERVE C13, 100.4808425
DECOUPLE H1, 399.6073319
Power 36 dB
continuously on
WALTZ-16 modulated

DATA PROCESSING
Line broadening 1.0 Hz
FT size 131072
Total time 2.2 hours

MN32
in CDCl3

Sample Name:
MN32
Data Collected on:
[illegible]

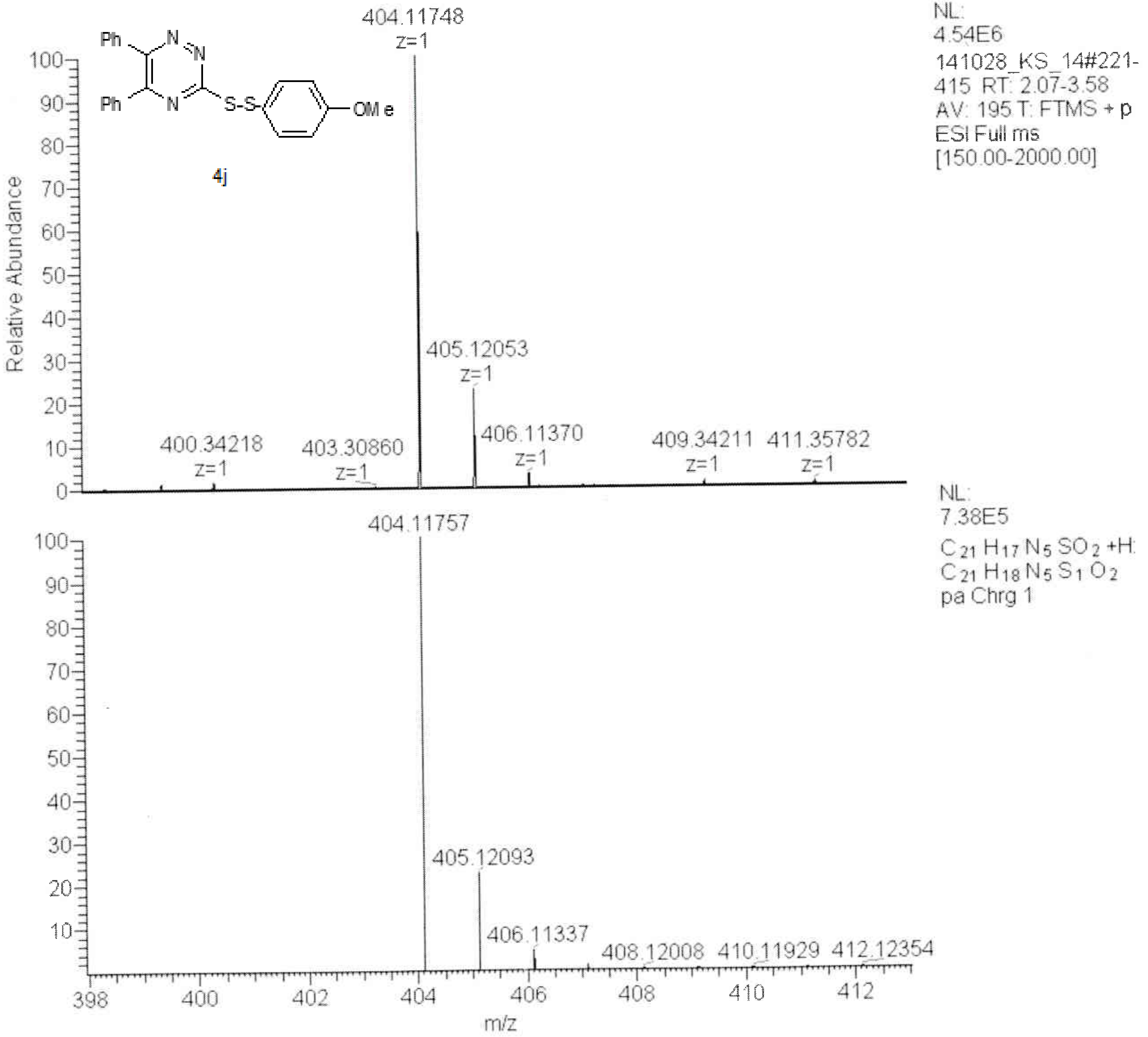

4j
NL: 4.54E6
141028_KS_14#221-415 RT: 2.07-3.58
AV: 195 T: FTMS + p ESI Full ms [150.00-2000.00]
NL: 7.38E5
C 21 H 17 N 5 SO 2 +H:
C 21 H 18 N 5 S 1 O 2
pa Chrg 1
404.11748 z=1
405.12053 z=1
406.11370 z=1
400.34218 z=1
403.30860 z=1
409.34211 z=1
411.35782 z=1
404.11757
405.12093
406.11337
408.12008
410.11929
412.12354
Relative Abundance
m/z

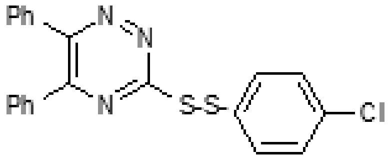

4k

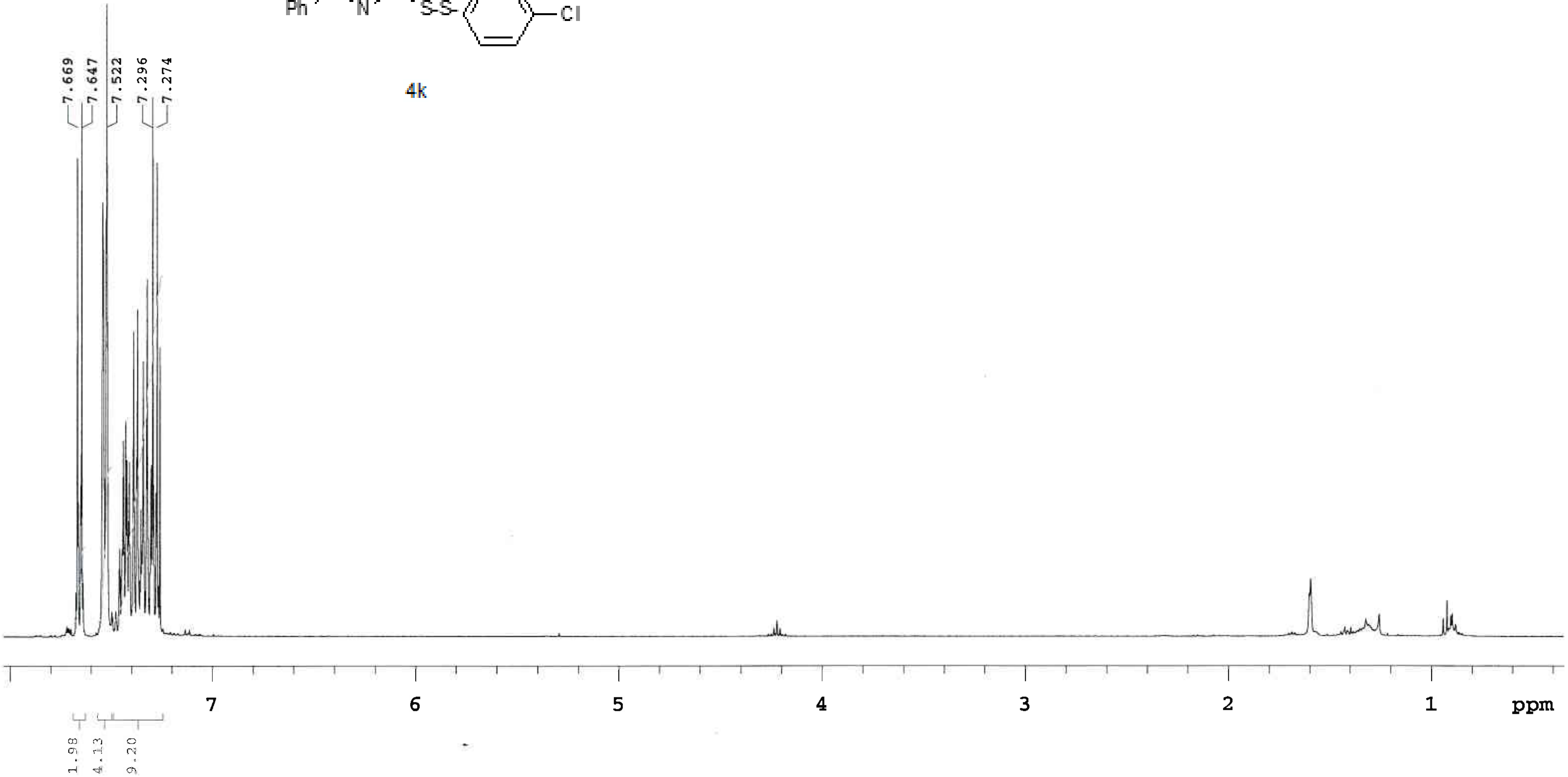

PULSE SEQUENCE
Relax. delay 0.500 sec
Pulse 48.6 degrees
Acq. time 4.797 sec
Width 6793.5 Hz
20 repetitions

OBSERVE H1, 399.6227914

DATA PROCESSING
FT size 131072
Total time 1 minutes

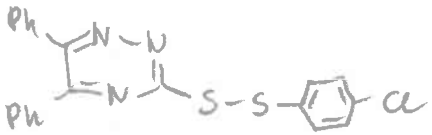

OSK28 in CDCl3

Sample Name:
OSK28
Data Collected on:
400MR-vnmrs400
Archive directory:
[illegible]

MP18a.
^{13}C NMR

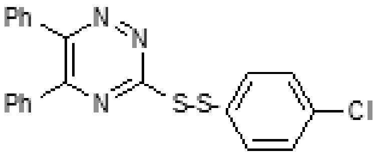

4k

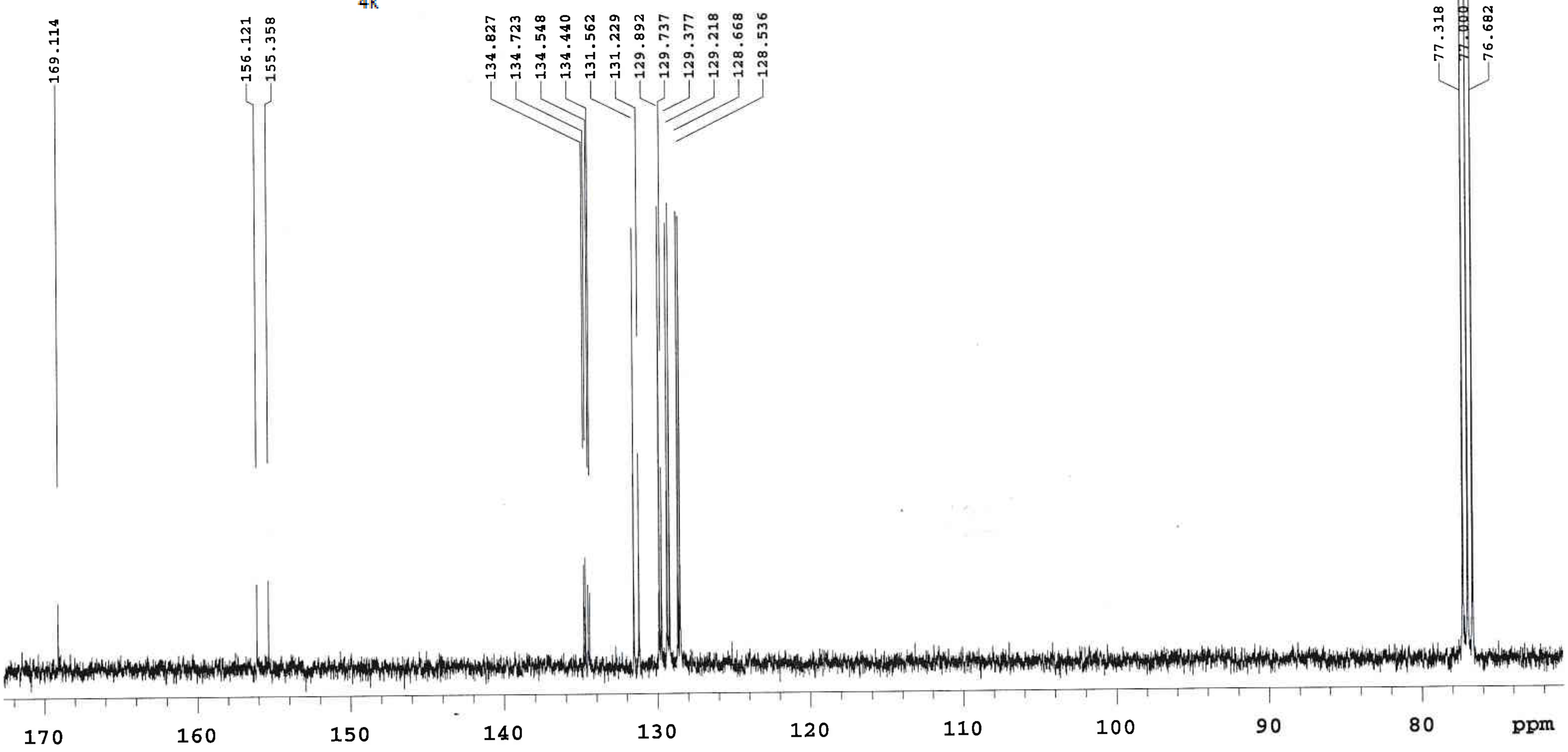

PULSE SEQUENCE
Relax. delay 1.500 sec
Pulse 38.5 degrees
Acq. time 2.000 sec
Width 25510.2 Hz
704 repetitions

OBSERVE C13, 100.4852336
DECOUPLE H1, 399.6247963
Power 36 dB
continuously on
WALTZ-16 modulated

DATA PROCESSING
Line broadening 1.0 Hz
FT size 131072
Total time 41 minutes

MP18a
in CDCl3

Sample Name:
MP18a
Data Collected on:
400MR-vnmrs400
Archive directory: [illegible]

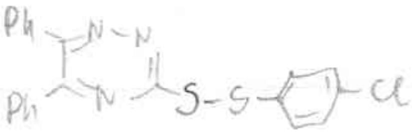

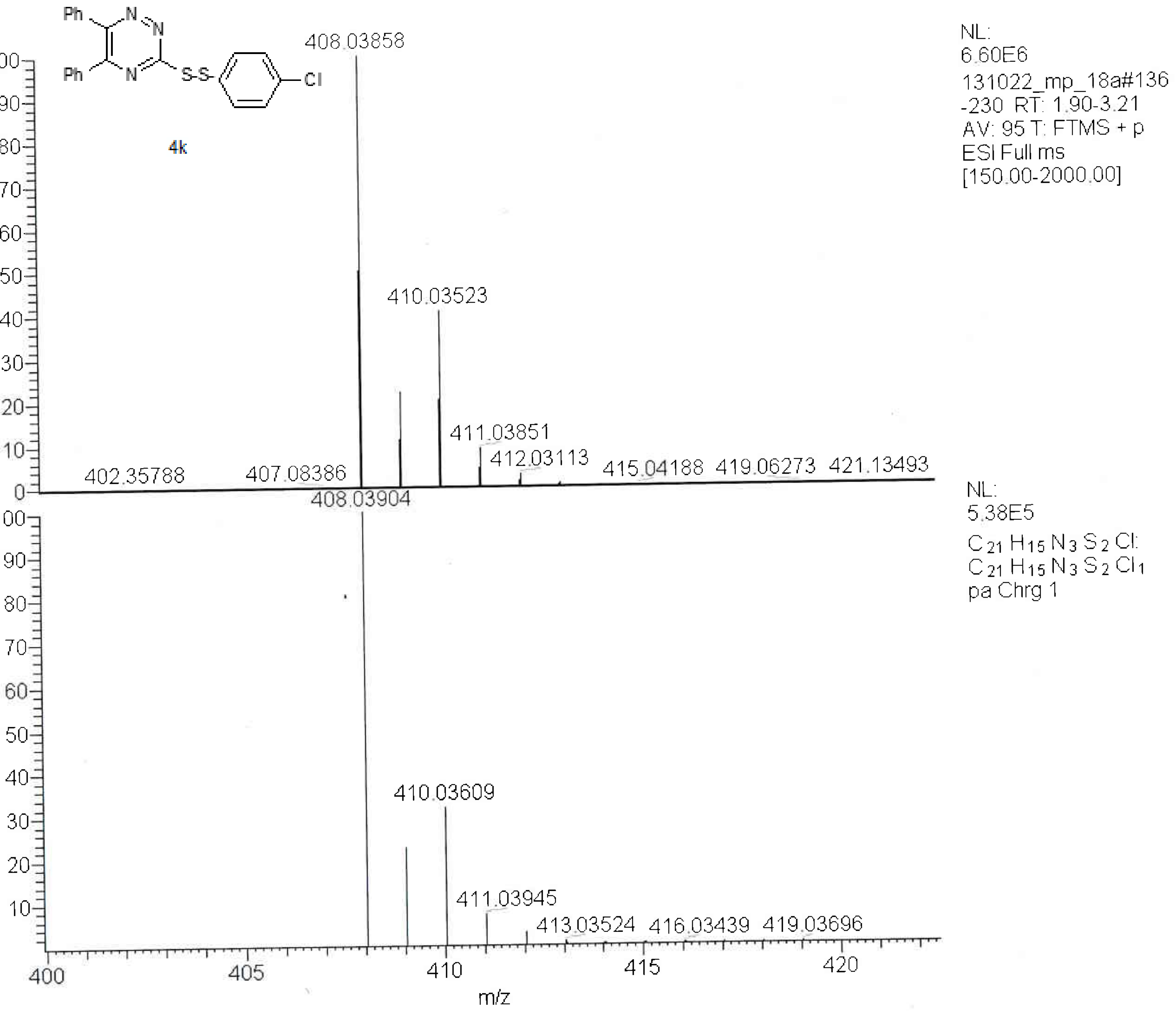

Ph
N
N
Ph
N
S-S
Cl
4k
408.03858
410.03523
411.03851
412.03113
402.35788
407.08386
415.04188
419.06273
421.13493
NL:
6.60E6
131022_mp_18a#136
-230 RT: 1.90-3.21
AV: 95 T: FTMS + p
ESI Full ms
[150.00-2000.00]
408.03904
410.03609
411.03945
413.03524
416.03439
419.03696
NL:
5.38E5
C 21 H 15 N 3 S 2 Cl:
C 21 H 15 N 3 S 2 Cl 1
pa Chrg 1
400
405
410
415
420
m/z

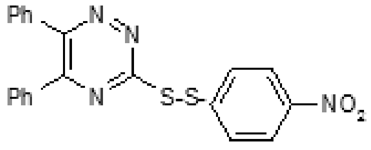

4l

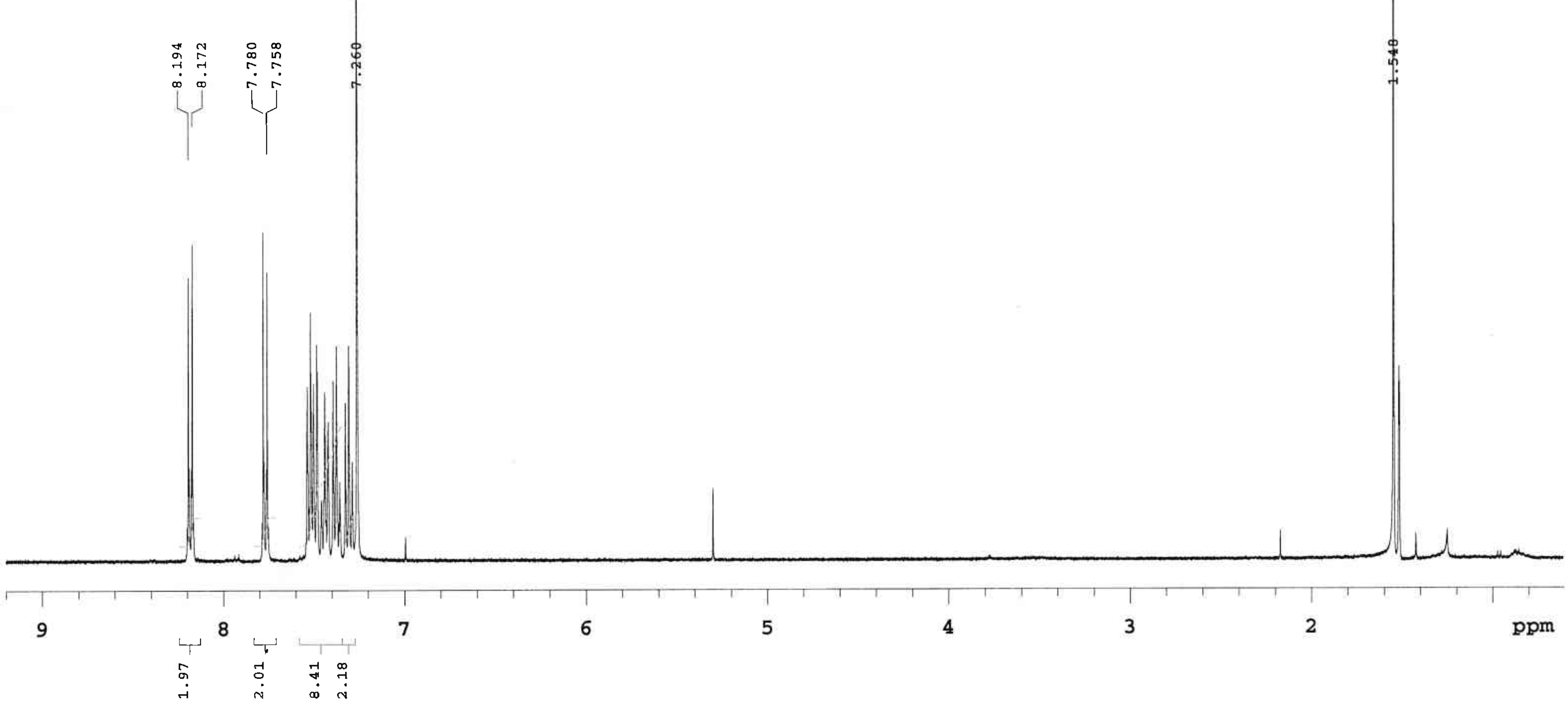

PULSE SEQUENCE
Relax. delay 0.500 sec
Pulse 48.6 degrees
Acq. time 4.797 sec
Width 6793.5 Hz
40 repetitions

OBSERVE H1, 399.6227914

DATA PROCESSING
FT size 131072
Total time 3 minutes

OSK41
in CDCl3

Sample Name:
OSK41
Data Collected on:
400MR-vnmrs400
Archive directory: [illegible]

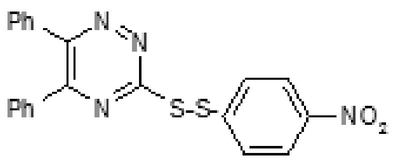
Ph
N
N
Ph
N
S-S
NO2

4l

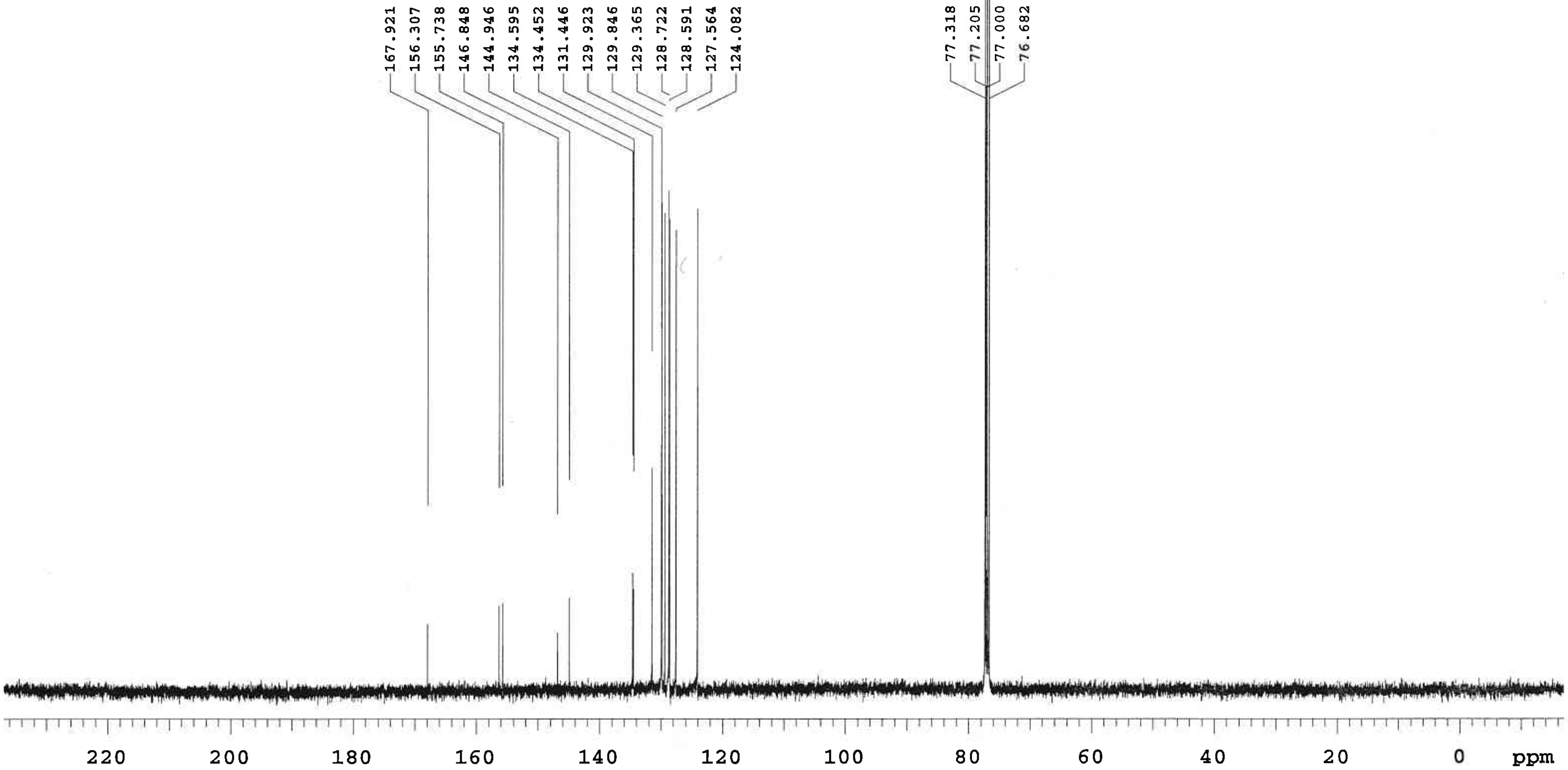
167.921
156.307
155.738
146.848
144.946
134.595
134.452
131.446
129.923
129.846
129.365
128.722
128.591
127.564
124.082
77.318
77.205
77.000
76.682
220
200
180
160
140
120
100
80
60
40
20
0
ppm

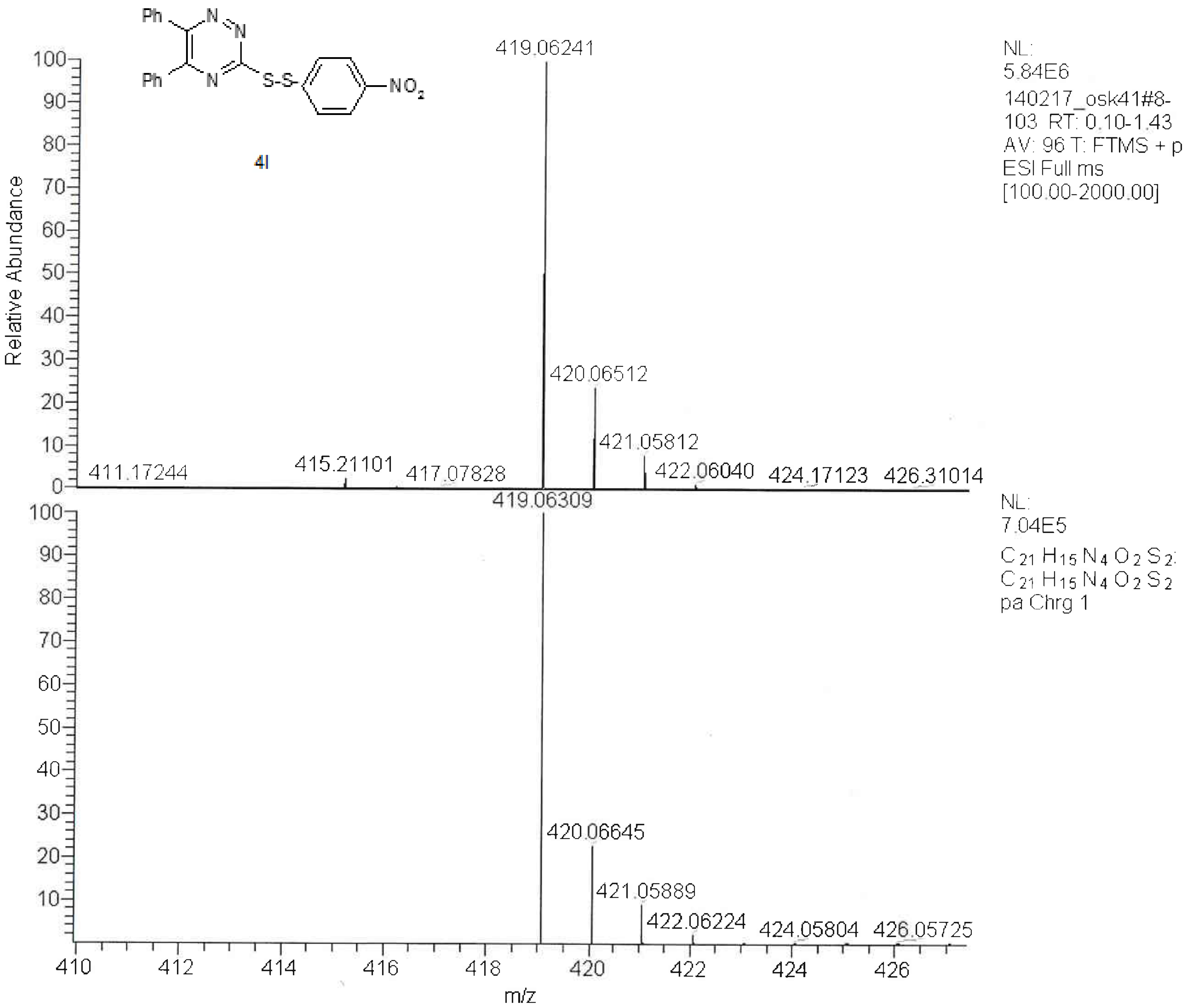
Ph
N
N
Ph
N
S-S
NO2
4l
419.06241
NL:
5.84E6
140217_osk41#8-103 RT: 0.10-1.43 AV: 96 T: FTMS + p ESI Full ms [100.00-2000.00]
Relative Abundance
420.06512
421.05812
411.17244
415.21101
417.07828
422.06040
424.17123
426.31014
419.06309
NL:
7.04E5
C 21 H 15 N 4 O 2 S 2:
C 21 H 15 N 4 O 2 S 2
pa Chrg 1
420.06645
421.05889
422.06224
424.05804
426.05725
m/z

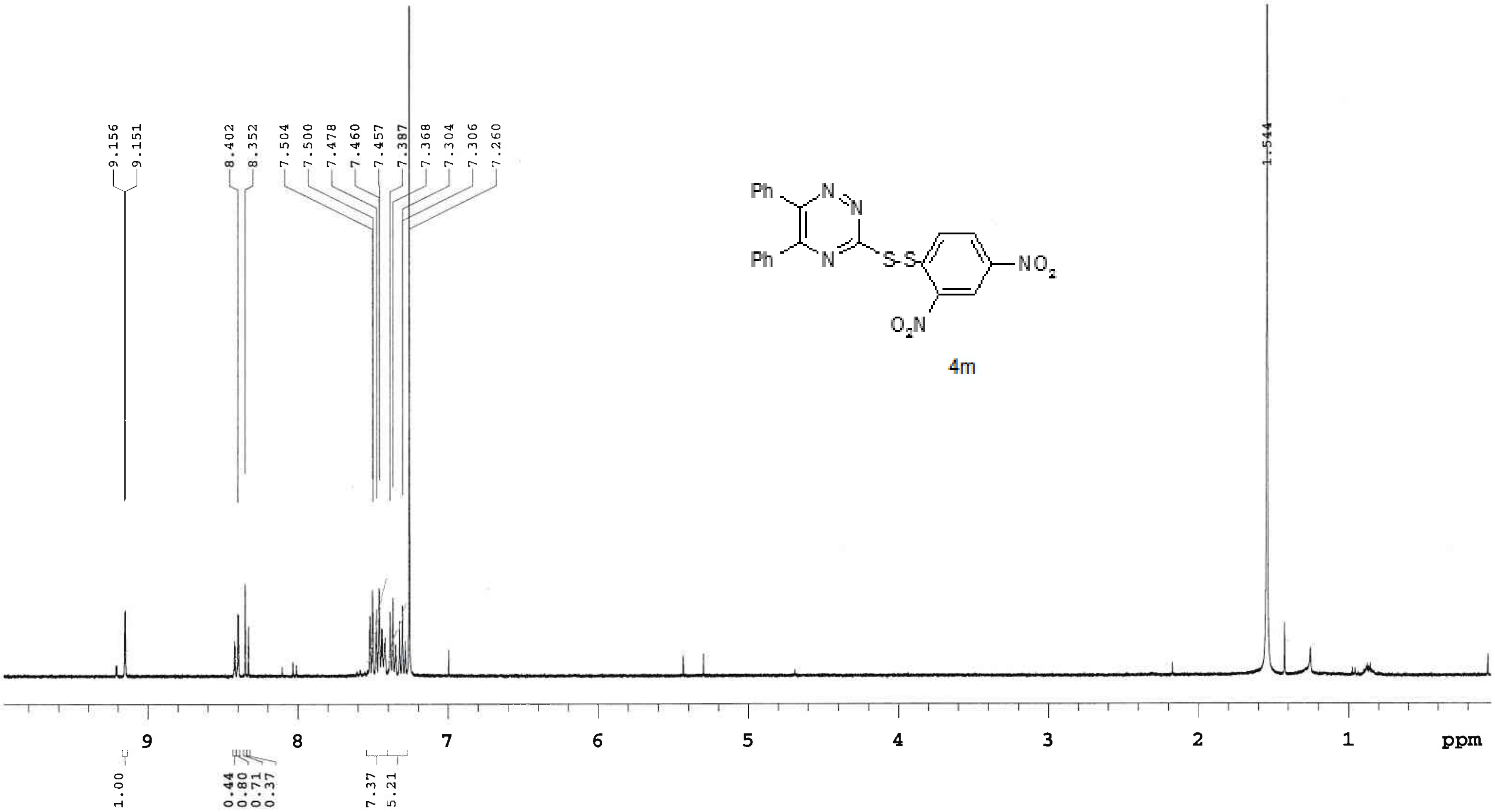

PULSE SEQUENCE
Relax. delay 0.500 sec
Pulse 48.6 degrees
Acq. time 4.797 sec
Width 6793.5 Hz
40 repetitions

OBSERVE H1, 399.6136843

DATA PROCESSING
FT size 131072
Total time 3 minutes

Osk48
in CDCl3

Sample Name:
Osk48
Data Collected on:
400MR-vnmrs400
Archive directory: [illegible]

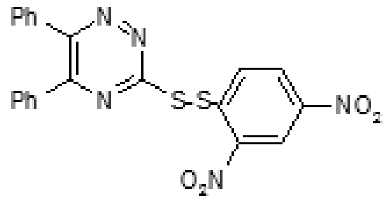

4m

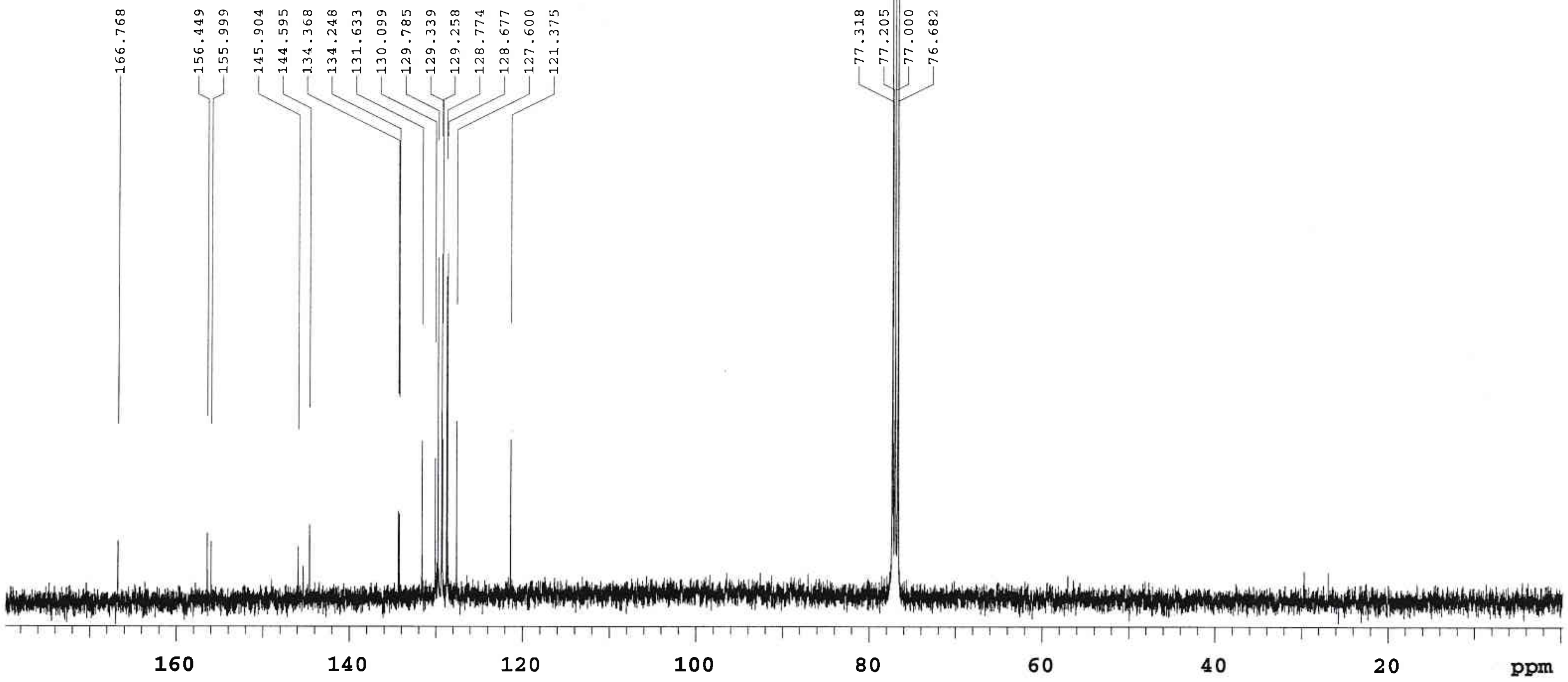

PULSE SEQUENCE
Relax. delay 1.500 sec
Pulse 38.5 degrees
Acq. time 2.000 sec
Width 25510.2 Hz
6000 repetitions

OBSERVE C13, 100.4829418
DECOUPLE H1, 399.6156840
Power 36 dB
continuously on
WALTZ-16 modulated

DATA PROCESSING
Line broadening 1.0 Hz
FT size 131072
Total time 5.8 hours

Osk48
in CDCl3

Sample Name:
Osk48
Data Collected on:
400MR-vnmrs400
Archive directory: [illegible]

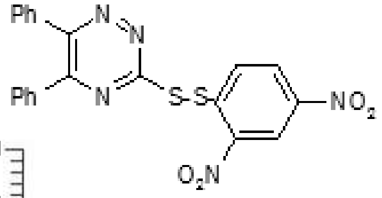
Ph
N
N
Ph
N
S-S
NO2
O2N

4m

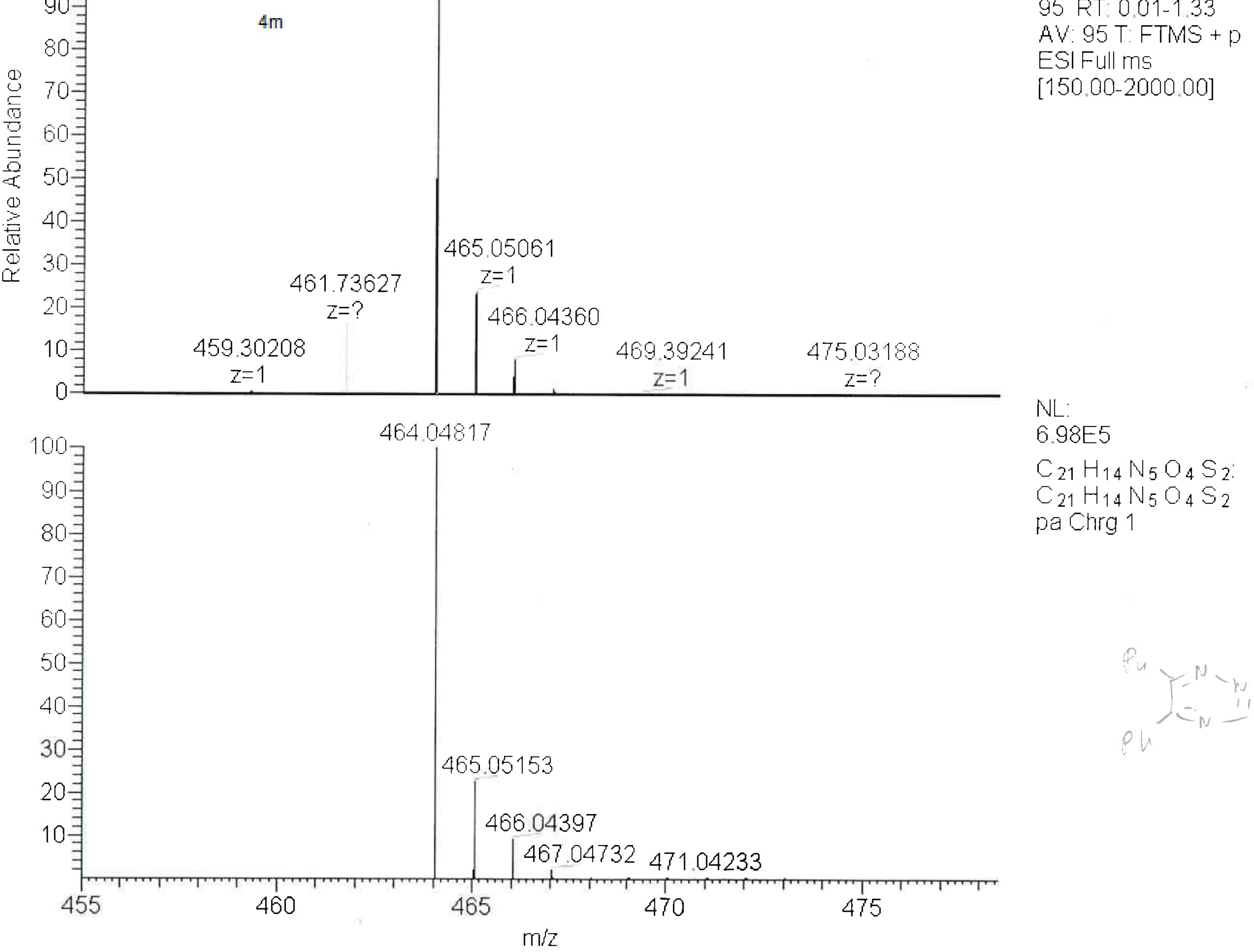
464.04776
z=1
Relative Abundance
461.73627
z=?
465.05061
z=1
466.04360
z=1
459.30208
z=1
469.39241
z=1
475.03188
z=?
NL:
1.82E6
140311_OSK48#1-
95 RT: 0.01-1.33
AV: 95 T: FTMS + p
ESI Full ms
[150.00-2000.00]
464.04817
465.05153
466.04397
467.04732
471.04233
NL:
6.98E5
C21 H14 N5 O4 S2:
C21 H14 N5 O4 S2
pa Chrg 1
m/z

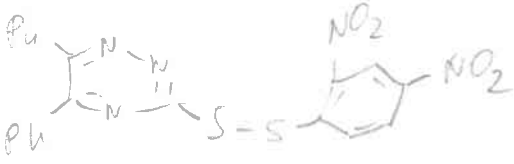

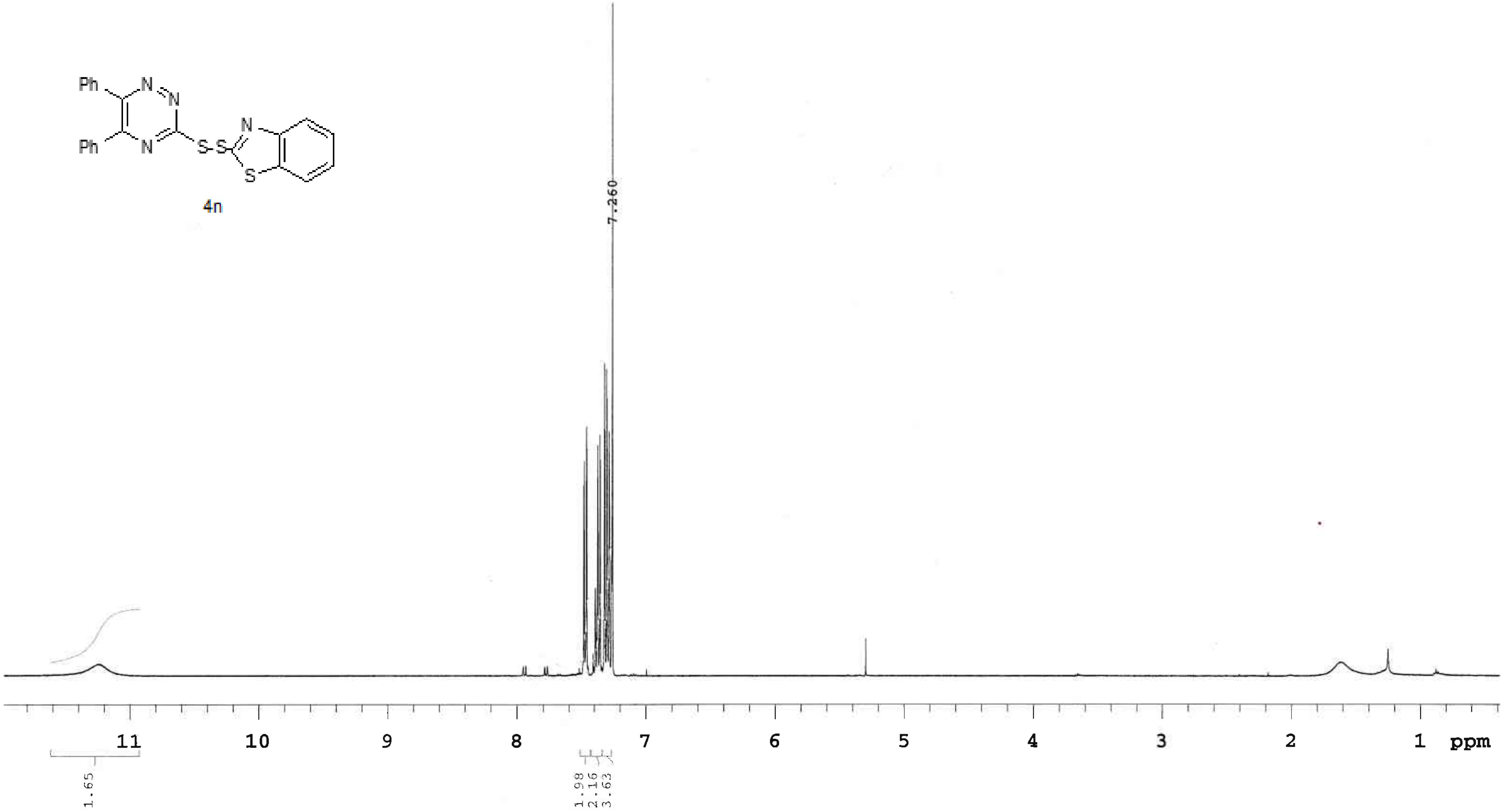

4n

PULSE SEQUENCE
Relax. delay 0.500 sec
Pulse 48.6 degrees
Acq. time 4.797 sec
Width 6793.5 Hz
32 repetitions

OBSERVE H1, 399.6227914

DATA PROCESSING
FT size 131072
Total time 2 minutes

OSK43a
in CDCl3

Sample Name:
OSK43a
Data Collected on:
400MR-vnmrs400
Archive directory:
[illegible]

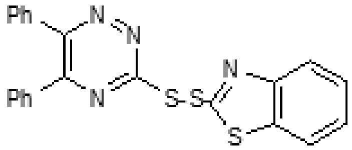

4n

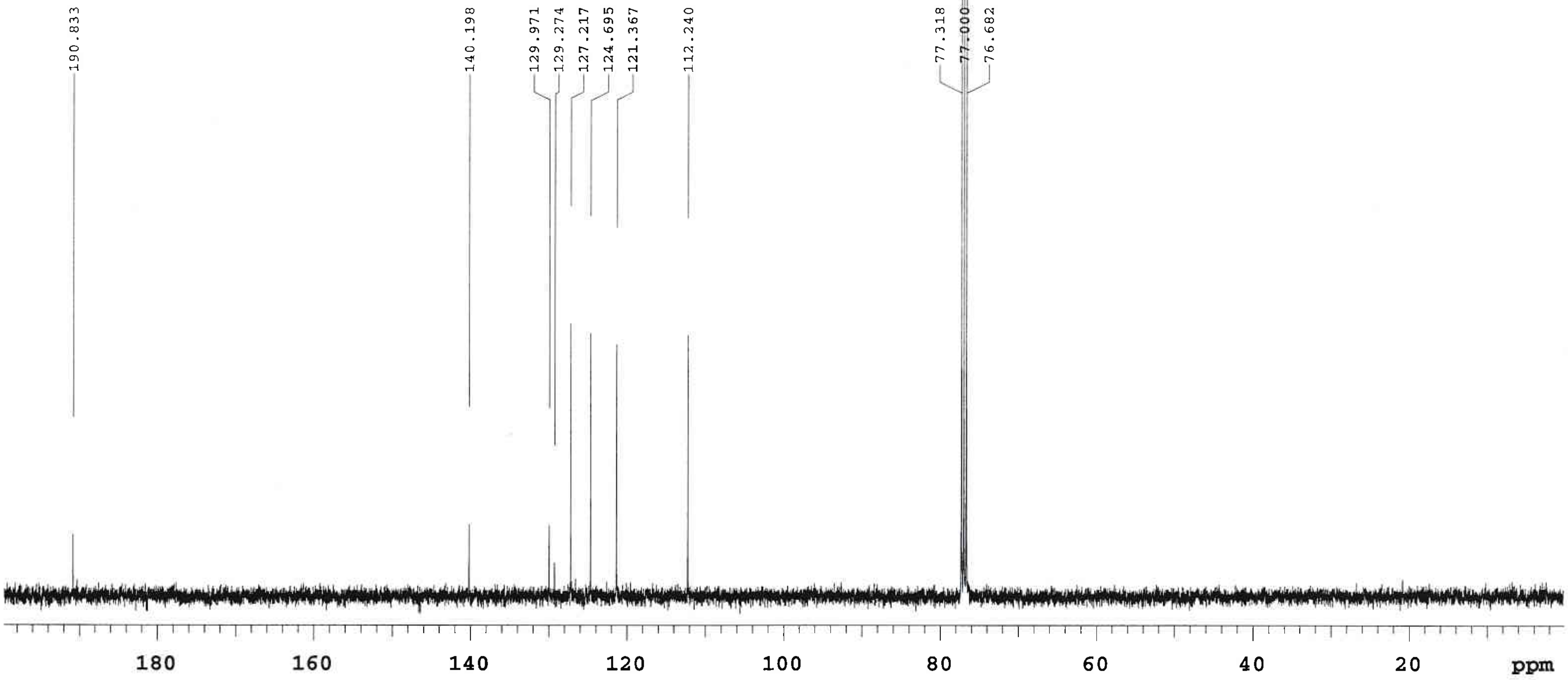

PULSE SEQUENCE
Relax. delay 1.500 sec
Pulse 38.5 degrees
Acq. time 2.000 sec
Width 25510.2 Hz
768 repetitions

OBSERVE C13, 100.4829434
DECOUPLE H1, 399.6156840
Power 36 dB
continuously on
WALTZ-16 modulated

DATA PROCESSING
Line broadening 1.0 Hz
FT size 131072
Total time 44 minutes

Osk43a
in CDCl3

Sample Name:
Osk43a
Data Collected on:
400MR-vnmrs400
Archive directory: [illegible]

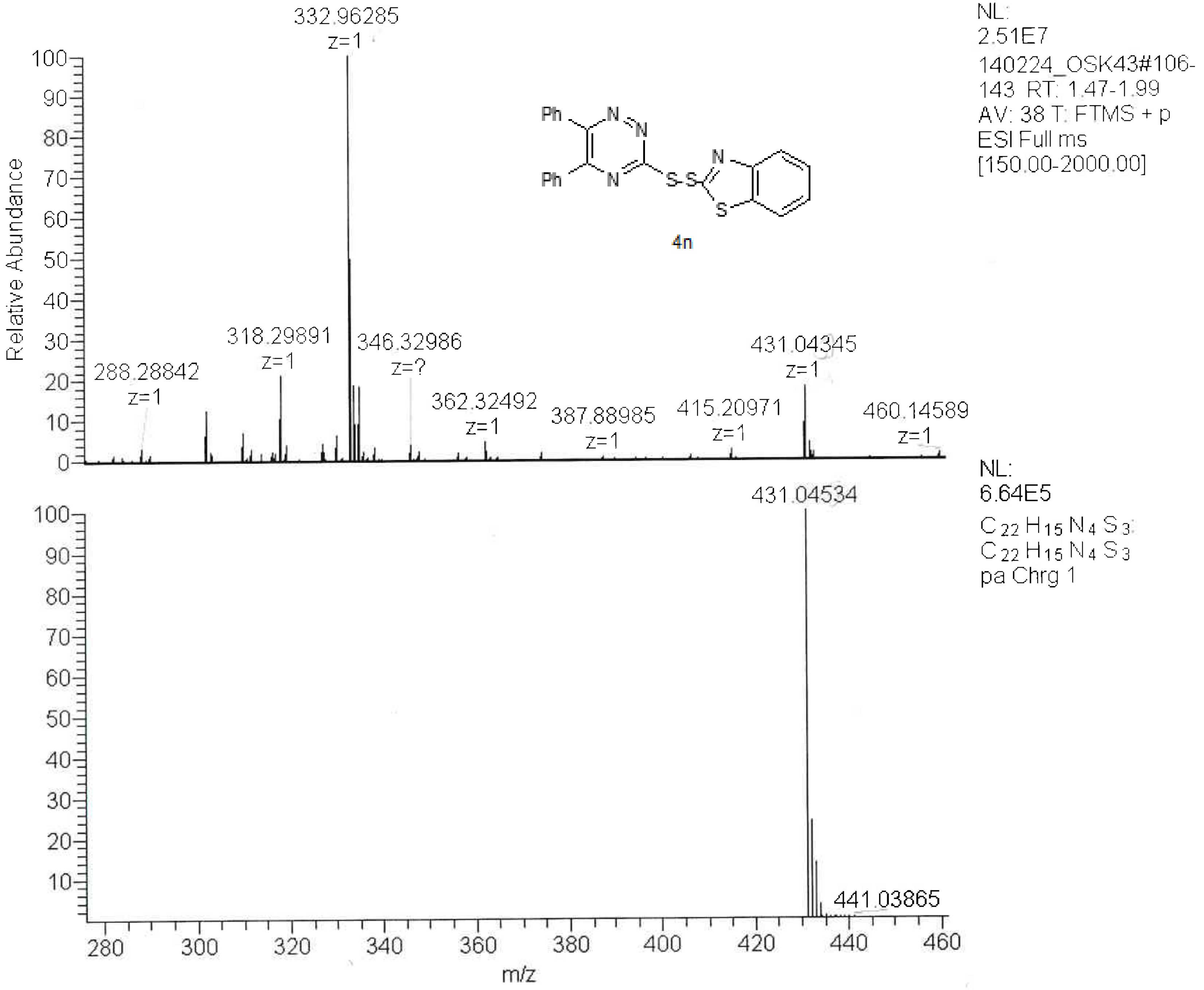

332.96285
z=1
318.29891
z=1
346.32986
z=?
288.28842
z=1
362.32492
z=1
387.88985
z=1
415.20971
z=1
431.04345
z=1
460.14589
z=1
Relative Abundance
Ph
N
N
Ph
N
S
S
N
S
4n
NL:
2.51E7
140224_OSK43#106-143 RT: 1.47-1.99
AV: 38 T: FTMS + p
ESI Full ms
[150.00-2000.00]
431.04534
441.03865
NL:
6.64E5
C 22 H 15 N 4 S 3:
C 22 H 15 N 4 S 3
pa Chrg 1
m/z

```
data_4d

_audit_creation_method            'SHELXL-2014/7'
_shelx_SHELXL_version_number      '2014/7'
_chemical_name_systematic         ?
_chemical_name_common             ?
_chemical_melting_point           ?
_chemical_formula_moiety          'C19 H19 N3 S2'
_chemical_formula_sum             'C19 H19 N3 S2'
_chemical_formula_weight          353.49

loop_
 _atom_type_symbol
 _atom_type_description
 _atom_type_scat_dispersion_real
 _atom_type_scat_dispersion_imag
 _atom_type_scat_source
 'C'  'C'   0.0181   0.0091
 'International Tables Vol C Tables 4.2.6.8 and 6.1.1.4'
 'H'  'H'   0.0000   0.0000
 'International Tables Vol C Tables 4.2.6.8 and 6.1.1.4'
 'N'  'N'   0.0311   0.0180
 'International Tables Vol C Tables 4.2.6.8 and 6.1.1.4'
 'S'  'S'   0.3331   0.5567
 'International Tables Vol C Tables 4.2.6.8 and 6.1.1.4'

_space_group_crystal_system       monoclinic
_space_group_IT_number            14
_space_group_name_H-M_alt         'P 21/n'
_space_group_name_Hall            '-P 2yn'

_shelx_space_group_comment
;
The symmetry employed for this shelxl refinement is uniquely defined
by the following loop, which should always be used as a source of
symmetry information in preference to the above space-group names.
They are only intended as comments.
;

loop_
 _space_group_symop_operation_xyz
 'x, y, z'
 '-x+1/2, y+1/2, -z+1/2'
 '-x, -y, -z'
 'x-1/2, -y-1/2, z-1/2'

_cell_length_a                    12.6017(6)
_cell_length_b                    10.2269(3)
_cell_length_c                    15.0464(7)
_cell_angle_alpha                 90
_cell_angle_beta                  112.653(5)
_cell_angle_gamma                 90
_cell_volume                      1789.53(14)
_cell_formula_units_Z             4
_cell_measurement_temperature     120.01(10)
_cell_measurement_reflns_used     6467
_cell_measurement_theta_min       3.8770
_cell_measurement_theta_max       76.0730
```

```
_exptl_crystal_description        prism
_exptl_crystal_colour             'clear yellow'
_exptl_crystal_density_meas       ?
_exptl_crystal_density_method     ?
_exptl_crystal_density_diffrn     1.312
_exptl_crystal_F_000              744
_exptl_transmission_factor_min    ?
_exptl_transmission_factor_max    ?
_exptl_crystal_size_max           0.30
_exptl_crystal_size_mid           0.30
_exptl_crystal_size_min           0.20
_exptl_absorpt_coefficient_mu     2.722
_shelx_estimated_absorpt_T_min    ?
_shelx_estimated_absorpt_T_max    ?
_exptl_absorpt_correction_type    'multi-scan'
_exptl_absorpt_correction_T_min   0.47330
_exptl_absorpt_correction_T_max   1.00000
_exptl_absorpt_process_details
;
CrysAlisPro, Agilent Technologies,
Version 1.171.37.34 (release 22-05-2014 CrysAlis171 .NET)
(compiled May 22 2014,16:03:01)
Empirical absorption correction using spherical harmonics,
 implemented in SCALE3 ABSPACK scaling algorithm.
Empirical absorption correction using spherical harmonics,
 implemented in SCALE3 ABSPACK scaling algorithm.
;
_exptl_absorpt_special_details    ?
_diffrn_ambient_temperature       120.01(10)
_diffrn_radiation_wavelength      1.54178
_diffrn_radiation_type            CuK\a
_diffrn_source                    'sealed X-ray tube'
_diffrn_measurement_device_type   'SuperNova, Single source at offset, AtlasS2'
_diffrn_measurement_method        '\f and \w scans'
_diffrn_detector_area_resol_mean  5.2763
_diffrn_reflns_number             11499
_diffrn_reflns_av_unetI/netI      0.0601
_diffrn_reflns_av_R_equivalents   0.0730
_diffrn_reflns_limit_h_min        -15
_diffrn_reflns_limit_h_max        10
_diffrn_reflns_limit_k_min        -12
_diffrn_reflns_limit_k_max        12
_diffrn_reflns_limit_l_min        -16
_diffrn_reflns_limit_l_max        18
_diffrn_reflns_theta_min          3.906
_diffrn_reflns_theta_max          76.313
_diffrn_reflns_theta_full         67.679
_diffrn_measured_fraction_theta_max   0.985
_diffrn_measured_fraction_theta_full  1.000
_diffrn_reflns_Laue_measured_fraction_max    0.985
_diffrn_reflns_Laue_measured_fraction_full   1.000
_diffrn_reflns_point_group_measured_fraction_max   0.985
_diffrn_reflns_point_group_measured_fraction_full  1.000
_reflns_number_total              3684
_reflns_number_gt                 3139
_reflns_threshold_expression      'I > 2\s(I)'
_reflns_Friedel_coverage          0.000
_reflns_Friedel_fraction_max      .
_reflns_Friedel_fraction_full     .

_reflns_special_details
```

```
;
 Reflections were merged by SHELXL according to the crystal
 class for the calculation of statistics and refinement.

 _reflns_Friedel_fraction is defined as the number of unique
 Friedel pairs measured divided by the number that would be
 possible theoretically, ignoring centric projections and
 systematic absences.
;

_computing_data_collection
;
CrysAlisPro, Agilent Technologies,
Version 1.171.37.34 (release 22-05-2014 CrysAlis171 .NET)
(compiled May 22 2014,16:03:01)
;
_computing_cell_refinement
;
CrysAlisPro, Agilent Technologies,
Version 1.171.37.34 (release 22-05-2014 CrysAlis171 .NET)
(compiled May 22 2014,16:03:01)
;
_computing_data_reduction
;
CrysAlisPro, Agilent Technologies,
Version 1.171.37.34 (release 22-05-2014 CrysAlis171 .NET)
(compiled May 22 2014,16:03:01)
;
_computing_structure_solution     'SHELXS-2013/1 (Sheldrick, 2014)'
_computing_structure_refinement   'SHELXL-2014/7 (Sheldrick, 2014)'
_computing_molecular_graphics     'ORTEP3 for Windows'
_computing_publication_material   'SHELXL-2014/7 and WINGX'
_refine_special_details           ?
_refine_ls_structure_factor_coef  Fsqd
_refine_ls_matrix_type            full
_refine_ls_weighting_scheme       calc
_refine_ls_weighting_details
'w=1/[\s^2^(Fo^2^)+(0.1071P)^2^+0.6002P] where P=(Fo^2^+2Fc^2^)/3'
_atom_sites_solution_primary      difmap
_atom_sites_solution_secondary    difmap
_atom_sites_solution_hydrogens    geom
_refine_ls_hydrogen_treatment     constr
_refine_ls_extinction_method      'SHELXL-2014/7 (Sheldrick 2014'
_refine_ls_extinction_coef        0.0012(4)
_refine_ls_extinction_expression
 'Fc^*^=kFc[1+0.001xFc^2^\l^3^/sin(2\q)]^-1/4^'
_refine_ls_number_reflns          3684
_refine_ls_number_parameters      220
_refine_ls_number_restraints      0
_refine_ls_R_factor_all           0.0665
_refine_ls_R_factor_gt            0.0556
_refine_ls_wR_factor_ref          0.1774
_refine_ls_wR_factor_gt           0.1521
_refine_ls_goodness_of_fit_ref    1.110
_refine_ls_restrained_S_all       1.110
_refine_ls_shift/su_max           0.001
_refine_ls_shift/su_mean          0.000

loop_
 _atom_site_label
 _atom_site_type_symbol
```

_atom_site_fract_x
_atom_site_fract_y
_atom_site_fract_z
_atom_site_U_iso_or_equiv
_atom_site_adp_type
_atom_site_occupancy
_atom_site_site_symmetry_order
_atom_site_calc_flag
_atom_site_refinement_flags_posn
_atom_site_refinement_flags_adp
_atom_site_refinement_flags_occupancy
_atom_site_disorder_assembly
_atom_site_disorder_group
N1 N 0.41375(19) 0.8153(2) 0.58048(15) 0.0237(5) Uani 1 1 d . . . . .
N2 N 0.4585(2) 0.7012(2) 0.62247(16) 0.0244(5) Uani 1 1 d . . . . .
N4 N 0.42304(17) 0.76114(19) 0.76172(14) 0.0183(4) Uani 1 1 d . . . . .
S7 S 0.49214(6) 0.51944(6) 0.75951(4) 0.0236(2) Uani 1 1 d . . . . .
S8 S 0.45427(6) 0.39810(6) 0.64399(5) 0.0257(2) Uani 1 1 d . . . . .
C3 C 0.4509(2) 0.6757(2) 0.70678(17) 0.0188(5) Uani 1 1 d . . . . .
C5 C 0.3872(2) 0.8784(2) 0.72344(17) 0.0169(5) Uani 1 1 d . . . . .
C6 C 0.3718(2) 0.9005(2) 0.62574(17) 0.0190(5) Uani 1 1 d . . . . .
C9 C 0.5804(2) 0.4073(2) 0.61373(18) 0.0226(5) Uani 1 1 d . . . . .
H9A H 0.5634 0.3625 0.5530 0.034 Uiso 1 1 calc R U . . .
H9B H 0.5945 0.4984 0.6041 0.034 Uiso 1 1 calc R U . . .
C10 C 0.6904(2) 0.3494(2) 0.68799(18) 0.0231(5) Uani 1 1 d . . . . .
H10 H 0.7042 0.3893 0.7507 0.035 Uiso 1 1 calc R U . . .
C11 C 0.7904(3) 0.3830(3) 0.6596(3) 0.0397(7) Uani 1 1 d . . . . .
H11A H 0.7921 0.4757 0.6502 0.060 Uiso 1 1 calc R U . . .
H11B H 0.7813 0.3385 0.6009 0.060 Uiso 1 1 calc R U . . .
H11C H 0.8611 0.3560 0.7098 0.060 Uiso 1 1 calc R U . . .
C12 C 0.6815(3) 0.2020(3) 0.6971(2) 0.0325(6) Uani 1 1 d . . . . .
H12A H 0.6198 0.1823 0.7174 0.049 Uiso 1 1 calc R U . . .
H12B H 0.7524 0.1691 0.7438 0.049 Uiso 1 1 calc R U . . .
H12C H 0.6667 0.1616 0.6359 0.049 Uiso 1 1 calc R U . . .
C51 C 0.3700(2) 0.9788(2) 0.78729(17) 0.0169(5) Uani 1 1 d . . . . .
C52 C 0.3386(2) 0.9397(2) 0.86253(18) 0.0233(5) Uani 1 1 d . . . . .
H52 H 0.3244 0.8518 0.8695 0.035 Uiso 1 1 calc R U . . .
C53 C 0.3285(2) 1.0305(2) 0.92669(19) 0.0244(5) Uani 1 1 d . . . . .
H53 H 0.3069 1.0036 0.9764 0.037 Uiso 1 1 calc R U . . .
C54 C 0.3504(2) 1.1618(2) 0.91745(18) 0.0229(5) Uani 1 1 d . . . . .
H54 H 0.3443 1.2226 0.9612 0.034 Uiso 1 1 calc R U . . .
C55 C 0.3815(2) 1.2020(2) 0.84252(18) 0.0226(5) Uani 1 1 d . . . . .
H55 H 0.3954 1.2900 0.8357 0.034 Uiso 1 1 calc R U . . .
C56 C 0.3916(2) 1.1108(2) 0.77767(17) 0.0205(5) Uani 1 1 d . . . . .
H56 H 0.4128 1.1379 0.7278 0.031 Uiso 1 1 calc R U . . .
C61 C 0.3098(2) 1.0132(2) 0.56673(17) 0.0196(5) Uani 1 1 d . . . . .
C62 C 0.2002(2) 1.0456(2) 0.56318(18) 0.0234(5) Uani 1 1 d . . . . .
H62 H 0.1688 0.9999 0.6007 0.035 Uiso 1 1 calc R U . . .
C63 C 0.1384(2) 1.1462(3) 0.50356(19) 0.0290(6) Uani 1 1 d . . . . .
H63 H 0.0651 1.1666 0.5004 0.043 Uiso 1 1 calc R U . . .
C64 C 0.1855(2) 1.2162(2) 0.44863(18) 0.0279(6) Uani 1 1 d . . . . .
H64 H 0.1439 1.2835 0.4089 0.042 Uiso 1 1 calc R U . . .
C65 C 0.2948(3) 1.1854(2) 0.45320(19) 0.0280(6) Uani 1 1 d . . . . .
H65 H 0.3268 1.2331 0.4171 0.042 Uiso 1 1 calc R U . . .
C66 C 0.3565(3) 1.0840(2) 0.51136(18) 0.0249(5) Uani 1 1 d . . . . .
H66 H 0.4292 1.0632 0.5134 0.037 Uiso 1 1 calc R U . . .

loop_
_atom_site_aniso_label
_atom_site_aniso_U_11
_atom_site_aniso_U_22

_atom_site_aniso_U_33
 _atom_site_aniso_U_23
 _atom_site_aniso_U_13
 _atom_site_aniso_U_12
N1 0.0335(12) 0.0152(10) 0.0292(10) 0.0015(8) 0.0197(9) 0.0060(8)
N2 0.0358(12) 0.0139(10) 0.0319(11) 0.0037(8) 0.0221(9) 0.0060(8)
N4 0.0180(10) 0.0138(9) 0.0265(9) 0.0001(7) 0.0122(8) -0.0004(7)
S7 0.0341(4) 0.0119(3) 0.0333(4) 0.0035(2) 0.0222(3) 0.0049(2)
S8 0.0270(4) 0.0139(3) 0.0411(4) -0.0040(2) 0.0185(3) 0.0012(2)
C3 0.0214(12) 0.0126(10) 0.0271(11) -0.0012(8) 0.0145(9) 0.0008(9)
C5 0.0179(11) 0.0111(10) 0.0264(11) 0.0013(8) 0.0136(9) -0.0005(8)
C6 0.0232(12) 0.0130(11) 0.0269(11) 0.0001(8) 0.0163(10) 0.0016(9)
C9 0.0311(14) 0.0188(12) 0.0245(11) 0.0030(9) 0.0180(10) 0.0078(10)
C10 0.0296(14) 0.0195(12) 0.0253(11) 0.0030(9) 0.0161(10) 0.0071(10)
C11 0.0326(16) 0.0337(16) 0.064(2) 0.0042(14) 0.0308(15) 0.0079(13)
C12 0.0423(17) 0.0226(14) 0.0347(14) 0.0055(11) 0.0171(12) 0.0108(12)
C51 0.0164(11) 0.0133(11) 0.0257(11) -0.0012(8) 0.0131(9) 0.0017(8)
C52 0.0316(14) 0.0146(11) 0.0304(12) 0.0004(9) 0.0195(11) -0.0008(10)
C53 0.0350(15) 0.0190(12) 0.0289(12) -0.0009(9) 0.0230(11) -0.0018(10)
C54 0.0260(13) 0.0194(12) 0.0297(12) -0.0065(9) 0.0179(10) -0.0005(9)
C55 0.0260(13) 0.0116(11) 0.0324(12) -0.0032(9) 0.0137(10) -0.0010(9)
C56 0.0248(12) 0.0144(11) 0.0261(11) 0.0004(9) 0.0138(10) -0.0020(9)
C61 0.0252(13) 0.0122(11) 0.0247(11) -0.0017(8) 0.0133(10) 0.0024(9)
C62 0.0260(13) 0.0167(12) 0.0284(12) -0.0008(9) 0.0115(10) -0.0001(9)
C63 0.0313(14) 0.0222(13) 0.0310(13) -0.0017(10) 0.0094(11) 0.0065(11)
C64 0.0369(15) 0.0158(12) 0.0277(12) 0.0020(9) 0.0087(11) 0.0036(10)
C65 0.0433(16) 0.0175(12) 0.0261(11) 0.0004(9) 0.0164(11) -0.0027(11)
C66 0.0372(15) 0.0173(11) 0.0271(12) -0.0004(9) 0.0198(11) 0.0003(10)

_geom_special_details
;
 All esds (except the esd in the dihedral angle between two l.s. planes)
 are estimated using the full covariance matrix. The cell esds are taken
 into account individually in the estimation of esds in distances, angles
 and torsion angles; correlations between esds in cell parameters are only
 used when they are defined by crystal symmetry. An approximate (isotropic)
 treatment of cell esds is used for estimating esds involving l.s. planes.
;

loop_
 _geom_bond_atom_site_label_1
 _geom_bond_atom_site_label_2
 _geom_bond_distance
 _geom_bond_site_symmetry_2
 _geom_bond_publ_flag
N1 C6 1.334(3) . ?
N1 N2 1.343(3) . ?
N2 C3 1.335(3) . ?
N4 C5 1.332(3) . ?
N4 C3 1.339(3) . ?
S7 C3 1.771(2) . ?
S7 S8 2.0372(9) . ?
S8 C9 1.816(3) . ?
C5 C6 1.425(3) . ?
C5 C51 1.478(3) . ?
C6 C61 1.481(3) . ?
C9 C10 1.526(3) . ?
C9 H9A 0.9700 . ?
C9 H9B 0.9700 . ?
C10 C11 1.518(4) . ?
C10 C12 1.522(4) . ?

C10 H10 0.9800 . ?
C11 H11A 0.9600 . ?
C11 H11B 0.9600 . ?
C11 H11C 0.9600 . ?
C12 H12A 0.9600 . ?
C12 H12B 0.9600 . ?
C12 H12C 0.9600 . ?
C51 C52 1.394(3) . ?
C51 C56 1.396(3) . ?
C52 C53 1.381(3) . ?
C52 H52 0.9300 . ?
C53 C54 1.389(4) . ?
C53 H53 0.9300 . ?
C54 C55 1.390(3) . ?
C54 H54 0.9300 . ?
C55 C56 1.391(3) . ?
C55 H55 0.9300 . ?
C56 H56 0.9300 . ?
C61 C66 1.394(3) . ?
C61 C62 1.402(4) . ?
C62 C63 1.389(4) . ?
C62 H62 0.9300 . ?
C63 C64 1.388(4) . ?
C63 H63 0.9300 . ?
C64 C65 1.389(4) . ?
C64 H64 0.9300 . ?
C65 C66 1.386(4) . ?
C65 H65 0.9300 . ?
C66 H66 0.9300 . ?

loop_
 _geom_angle_atom_site_label_1
 _geom_angle_atom_site_label_2
 _geom_angle_atom_site_label_3
 _geom_angle
 _geom_angle_site_symmetry_1
 _geom_angle_site_symmetry_3
 _geom_angle_publ_flag
C6 N1 N2 120.3(2) . . ?
C3 N2 N1 116.5(2) . . ?
C5 N4 C3 116.5(2) . . ?
C3 S7 S8 103.62(8) . . ?
C9 S8 S7 104.02(9) . . ?
N2 C3 N4 126.2(2) . . ?
N2 C3 S7 119.08(17) . . ?
N4 C3 S7 114.45(17) . . ?
N4 C5 C6 118.6(2) . . ?
N4 C5 C51 117.0(2) . . ?
C6 C5 C51 124.4(2) . . ?
N1 C6 C5 120.0(2) . . ?
N1 C6 C61 115.1(2) . . ?
C5 C6 C61 125.0(2) . . ?
C10 C9 S8 115.61(17) . . ?
C10 C9 H9A 108.4 . . ?
S8 C9 H9A 108.4 . . ?
C10 C9 H9B 108.4 . . ?
S8 C9 H9B 108.4 . . ?
H9A C9 H9B 107.4 . . ?
C11 C10 C12 110.3(2) . . ?
C11 C10 C9 109.0(2) . . ?
C12 C10 C9 111.9(2) . . ?

C11 C10 H10 108.5 . . ?
C12 C10 H10 108.5 . . ?
C9 C10 H10 108.5 . . ?
C10 C11 H11A 109.5 . . ?
C10 C11 H11B 109.5 . . ?
H11A C11 H11B 109.5 . . ?
C10 C11 H11C 109.5 . . ?
H11A C11 H11C 109.5 . . ?
H11B C11 H11C 109.5 . . ?
C10 C12 H12A 109.5 . . ?
C10 C12 H12B 109.5 . . ?
H12A C12 H12B 109.5 . . ?
C10 C12 H12C 109.5 . . ?
H12A C12 H12C 109.5 . . ?
H12B C12 H12C 109.5 . . ?
C52 C51 C56 119.3(2) . . ?
C52 C51 C5 119.1(2) . . ?
C56 C51 C5 121.4(2) . . ?
C53 C52 C51 120.4(2) . . ?
C53 C52 H52 119.8 . . ?
C51 C52 H52 119.8 . . ?
C52 C53 C54 120.3(2) . . ?
C52 C53 H53 119.8 . . ?
C54 C53 H53 119.8 . . ?
C53 C54 C55 119.7(2) . . ?
C53 C54 H54 120.1 . . ?
C55 C54 H54 120.1 . . ?
C54 C55 C56 120.1(2) . . ?
C54 C55 H55 119.9 . . ?
C56 C55 H55 119.9 . . ?
C55 C56 C51 120.1(2) . . ?
C55 C56 H56 120.0 . . ?
C51 C56 H56 120.0 . . ?
C66 C61 C62 119.4(2) . . ?
C66 C61 C6 121.1(2) . . ?
C62 C61 C6 119.5(2) . . ?
C63 C62 C61 119.9(2) . . ?
C63 C62 H62 120.0 . . ?
C61 C62 H62 120.0 . . ?
C64 C63 C62 120.3(3) . . ?
C64 C63 H63 119.9 . . ?
C62 C63 H63 119.9 . . ?
C63 C64 C65 119.8(2) . . ?
C63 C64 H64 120.1 . . ?
C65 C64 H64 120.1 . . ?
C66 C65 C64 120.3(3) . . ?
C66 C65 H65 119.8 . . ?
C64 C65 H65 119.8 . . ?
C65 C66 C61 120.2(3) . . ?
C65 C66 H66 119.9 . . ?
C61 C66 H66 119.9 . . ?

loop_
 _geom_torsion_atom_site_label_1
 _geom_torsion_atom_site_label_2
 _geom_torsion_atom_site_label_3
 _geom_torsion_atom_site_label_4
 _geom_torsion
 _geom_torsion_site_symmetry_1
 _geom_torsion_site_symmetry_2
 _geom_torsion_site_symmetry_3

_geom_torsion_site_symmetry_4
_geom_torsion_publ_flag
C6 N1 N2 C3 4.6(4) . . . . ?
N1 N2 C3 N4 -13.3(4) . . . . ?
N1 N2 C3 S7 172.51(18) . . . . ?
C5 N4 C3 N2 8.1(4) . . . . ?
C5 N4 C3 S7 -177.53(17) . . . . ?
S8 S7 C3 N2 -31.1(2) . . . . ?
S8 S7 C3 N4 154.09(16) . . . . ?
C3 N4 C5 C6 5.3(3) . . . . ?
C3 N4 C5 C51 -172.6(2) . . . . ?
N2 N1 C6 C5 7.8(4) . . . . ?
N2 N1 C6 C61 -171.5(2) . . . . ?
N4 C5 C6 N1 -13.1(3) . . . . ?
C51 C5 C6 N1 164.7(2) . . . . ?
N4 C5 C6 C61 166.2(2) . . . . ?
C51 C5 C6 C61 -16.1(4) . . . . ?
S7 S8 C9 C10 67.04(19) . . . . ?
S8 C9 C10 C11 -171.47(19) . . . . ?
S8 C9 C10 C12 66.2(2) . . . . ?
N4 C5 C51 C52 -29.6(3) . . . . ?
C6 C5 C51 C52 152.6(2) . . . . ?
N4 C5 C51 C56 146.2(2) . . . . ?
C6 C5 C51 C56 -31.6(4) . . . . ?
C56 C51 C52 C53 0.3(4) . . . . ?
C5 C51 C52 C53 176.2(2) . . . . ?
C51 C52 C53 C54 -0.5(4) . . . . ?
C52 C53 C54 C55 0.7(4) . . . . ?
C53 C54 C55 C56 -0.6(4) . . . . ?
C54 C55 C56 C51 0.4(4) . . . . ?
C52 C51 C56 C55 -0.2(4) . . . . ?
C5 C51 C56 C55 -176.0(2) . . . . ?
N1 C6 C61 C66 -47.3(3) . . . . ?
C5 C6 C61 C66 133.4(3) . . . . ?
N1 C6 C61 C62 129.8(2) . . . . ?
C5 C6 C61 C62 -49.4(3) . . . . ?
C66 C61 C62 C63 1.1(4) . . . . ?
C6 C61 C62 C63 -176.1(2) . . . . ?
C61 C62 C63 C64 -1.1(4) . . . . ?
C62 C63 C64 C65 0.1(4) . . . . ?
C63 C64 C65 C66 0.9(4) . . . . ?
C64 C65 C66 C61 -0.9(4) . . . . ?
C62 C61 C66 C65 -0.1(4) . . . . ?
C6 C61 C66 C65 177.1(2) . . . . ?

_refine_diff_density_max   0.490
_refine_diff_density_min   -0.548
_refine_diff_density_rms   0.100

_shelx_res_file
;

    shelx.res created by SHELXL-2014/7

TITL mn15 in P2(1)/n
CELL 1.54178 12.6017 10.2269 15.0464   90.000 112.653   90.000
ZERR   4.00   0.0006   0.0003   0.0007   0.000   0.005   0.000
LATT   1
SYMM 1/2 - X, 1/2 + Y, 1/2 - Z
SFAC C   H   N   S

```
UNIT  76 76 12 8
MERG   2
FMAP   2
ACTA
BOND   $H
L.S. 50
PLAN -2
CONF
WGHT    0.107100    0.600200
EXTI    0.001238
FVAR       0.13889
N1    3    0.413755    0.815266    0.580480    11.00000    0.03353    0.01516 =
         0.02921    0.00151    0.01974    0.00600
N2    3    0.458524    0.701217    0.622474    11.00000    0.03579    0.01391 =
         0.03188    0.00367    0.02214    0.00602
N4    3    0.423037    0.761136    0.761719    11.00000    0.01797    0.01383 =
         0.02652    0.00009    0.01222   -0.00039
S7    4    0.492141    0.519439    0.759515    11.00000    0.03406    0.01186 =
         0.03335    0.00355    0.02221    0.00493
S8    4    0.454273    0.398104    0.643987    11.00000    0.02697    0.01389 =
         0.04113   -0.00395    0.01850    0.00119
C3    1    0.450853    0.675686    0.706780    11.00000    0.02138    0.01256 =
         0.02713   -0.00117    0.01451    0.00083
C5    1    0.387224    0.878417    0.723442    11.00000    0.01786    0.01112 =
         0.02637    0.00127    0.01361   -0.00049
C6    1    0.371801    0.900521    0.625739    11.00000    0.02323    0.01302 =
         0.02687    0.00009    0.01633    0.00156
C9    1    0.580373    0.407310    0.613731    11.00000    0.03111    0.01885 =
         0.02450    0.00295    0.01800    0.00778
AFIX  23
H9A   2    0.563401    0.362530    0.552962    11.00000   -1.50000
H9B   2    0.594469    0.498446    0.604128    11.00000   -1.50000
AFIX   0
C10   1    0.690396    0.349384    0.687994    11.00000    0.02962    0.01952 =
         0.02525    0.00302    0.01608    0.00711
AFIX  13
H10   2    0.704206    0.389292    0.750694    11.00000   -1.50000
AFIX   0
C11   1    0.790432    0.382967    0.659637    11.00000    0.03260    0.03365 =
         0.06401    0.00417    0.03084    0.00793
AFIX 137
H11A  2    0.792148    0.475663    0.650218    11.00000   -1.50000
H11B  2    0.781311    0.338517    0.600943    11.00000   -1.50000
H11C  2    0.861113    0.355965    0.709813    11.00000   -1.50000
AFIX   0
C12   1    0.681536    0.201961    0.697131    11.00000    0.04231    0.02263 =
         0.03474    0.00546    0.01711    0.01077
AFIX 137
H12A  2    0.619834    0.182252    0.717439    11.00000   -1.50000
H12B  2    0.752447    0.169057    0.743811    11.00000   -1.50000
H12C  2    0.666739    0.161583    0.635906    11.00000   -1.50000
AFIX   0
C51   1    0.370015    0.978827    0.787285    11.00000    0.01636    0.01329 =
         0.02572   -0.00117    0.01314    0.00170
C52   1    0.338609    0.939656    0.862527    11.00000    0.03164    0.01463 =
         0.03038    0.00041    0.01949   -0.00078
AFIX  43
H52   2    0.324389    0.851810    0.869538    11.00000   -1.50000
AFIX   0
C53   1    0.328495    1.030497    0.926686    11.00000    0.03502    0.01897 =
         0.02894   -0.00087    0.02295   -0.00175
```

```
AFIX  43
H53   2    0.306892    1.003612    0.976354    11.00000   -1.50000
AFIX   0
C54   1    0.350408    1.161808    0.917448    11.00000    0.02600    0.01940 =
        0.02966   -0.00651    0.01789   -0.00054
AFIX  43
H54   2    0.344340    1.222620    0.961206    11.00000   -1.50000
AFIX   0
C55   1    0.381454    1.201981    0.842518    11.00000    0.02598    0.01155 =
        0.03243   -0.00316    0.01374   -0.00103
AFIX  43
H55   2    0.395446    1.289963    0.835749    11.00000   -1.50000
AFIX   0
C56   1    0.391619    1.110822    0.777672    11.00000    0.02475    0.01443 =
        0.02606    0.00040    0.01381   -0.00200
AFIX  43
H56   2    0.412845    1.137856    0.727831    11.00000   -1.50000
AFIX   0
C61   1    0.309795    1.013222    0.566730    11.00000    0.02518    0.01220 =
        0.02472   -0.00169    0.01328    0.00239
C62   1    0.200150    1.045644    0.563179    11.00000    0.02595    0.01669 =
        0.02843   -0.00084    0.01150   -0.00007
AFIX  43
H62   2    0.168816    0.999912    0.600690    11.00000   -1.50000
AFIX   0
C63   1    0.138353    1.146166    0.503562    11.00000    0.03134    0.02221 =
        0.03095   -0.00165    0.00936    0.00650
AFIX  43
H63   2    0.065093    1.166632    0.500440    11.00000   -1.50000
AFIX   0
C64   1    0.185479    1.216187    0.448635    11.00000    0.03690    0.01584 =
        0.02769    0.00200    0.00872    0.00355
AFIX  43
H64   2    0.143947    1.283525    0.408872    11.00000   -1.50000
AFIX   0
C65   1    0.294809    1.185440    0.453204    11.00000    0.04330    0.01751 =
        0.02606    0.00043    0.01637   -0.00267
AFIX  43
H65   2    0.326800    1.233133    0.417068    11.00000   -1.50000
AFIX   0
C66   1    0.356499    1.084015    0.511357    11.00000    0.03715    0.01727 =
        0.02712   -0.00036    0.01982    0.00028
AFIX  43
H66   2    0.429246    1.063158    0.513425    11.00000   -1.50000
AFIX   0
HKLF    4

REM  mn15 in P2(1)/n
REM R1 =  0.0556 for    3139 Fo > 4sig(Fo)  and  0.0665 for all    3684 data
REM    220 parameters refined using      0 restraints

END
```

```
data_4k

_audit_creation_method            'SHELXL-2014/7'
_shelx_SHELXL_version_number      '2014/7'
_chemical_name_systematic         ?
_chemical_name_common             ?
_chemical_melting_point           ?
_chemical_formula_moiety          'C21 H14 Cl N3 S2'
_chemical_formula_sum             'C21 H14 Cl N3 S2'
_chemical_formula_weight          407.92

loop_
 _atom_type_symbol
 _atom_type_description
 _atom_type_scat_dispersion_real
 _atom_type_scat_dispersion_imag
 _atom_type_scat_source
 'C'  'C'   0.0181   0.0091
 'International Tables Vol C Tables 4.2.6.8 and 6.1.1.4'
 'H'  'H'   0.0000   0.0000
 'International Tables Vol C Tables 4.2.6.8 and 6.1.1.4'
 'N'  'N'   0.0311   0.0180
 'International Tables Vol C Tables 4.2.6.8 and 6.1.1.4'
 'S'  'S'   0.3331   0.5567
 'International Tables Vol C Tables 4.2.6.8 and 6.1.1.4'
 'Cl'  'Cl'   0.3639   0.7018
 'International Tables Vol C Tables 4.2.6.8 and 6.1.1.4'

_space_group_crystal_system       monoclinic
_space_group_IT_number            15
_space_group_name_H-M_alt         'C 2/c'
_space_group_name_Hall            '-C 2yc'

_shelx_space_group_comment
;
The symmetry employed for this shelxl refinement is uniquely defined
by the following loop, which should always be used as a source of
symmetry information in preference to the above space-group names.
They are only intended as comments.
;

loop_
 _space_group_symop_operation_xyz
 'x, y, z'
 '-x, y, -z+1/2'
 'x+1/2, y+1/2, z'
 '-x+1/2, y+1/2, -z+1/2'
 '-x, -y, -z'
 'x, -y, z-1/2'
 '-x+1/2, -y+1/2, -z'
 'x+1/2, -y+1/2, z-1/2'

_cell_length_a                    17.3965(19)
_cell_length_b                    9.2049(6)
_cell_length_c                    24.2516(3)
_cell_angle_alpha                 90
_cell_angle_beta                  105.435(13)
_cell_angle_gamma                 90
_cell_volume                      3743.4(5)
```

```
_cell_formula_units_Z            8
_cell_measurement_temperature     120.01(10)
_cell_measurement_reflns_used     3543
_cell_measurement_theta_min       3.7760
_cell_measurement_theta_max       75.7790

_exptl_crystal_description        plate
_exptl_crystal_colour             'clear yellow'
_exptl_crystal_density_meas       ?
_exptl_crystal_density_method     ?
_exptl_crystal_density_diffrn     1.448
_exptl_crystal_F_000              1680
_exptl_transmission_factor_min    ?
_exptl_transmission_factor_max    ?
_exptl_crystal_size_max           0.32
_exptl_crystal_size_mid           0.23
_exptl_crystal_size_min           0.19
_exptl_absorpt_coefficient_mu     3.974
_shelx_estimated_absorpt_T_min    ?
_shelx_estimated_absorpt_T_max    ?
_exptl_absorpt_correction_type    multi-scan
_exptl_absorpt_correction_T_min   0.931
_exptl_absorpt_correction_T_max   0.954
_exptl_absorpt_process_details
;
CrysAlisPro, Agilent Technologies,
Version 1.171.37.34 (release 22-05-2014 CrysAlis171 .NET)
(compiled May 22 2014,16:03:01)
Analytical numeric absorption correction using a multifaceted crystal
                        model based on expressions derived by R.C. Clark & J.S. Reid.
(Clark, R. C. & Reid, J. S. (1995). Acta Cryst. A51, 887-897)
Empirical absorption correction using spherical harmonics,
 implemented in SCALE3 ABSPACK scaling algorithm.
;
_exptl_absorpt_special_details    ?
_diffrn_ambient_temperature       120.01(10)
_diffrn_radiation_wavelength      1.54178
_diffrn_radiation_type            CuK\a
_diffrn_source                    'sealed X-ray tube'
_diffrn_measurement_device_type   'SuperNova, Single source at offset, AtlasS2'
_diffrn_measurement_method        '\f and \w scans'
_diffrn_detector_area_resol_mean  5.2763
_diffrn_reflns_number             7216
_diffrn_reflns_av_unetI/netI      0.0645
_diffrn_reflns_av_R_equivalents   0.0555
_diffrn_reflns_limit_h_min        -20
_diffrn_reflns_limit_h_max        21
_diffrn_reflns_limit_k_min        -11
_diffrn_reflns_limit_k_max        11
_diffrn_reflns_limit_l_min        -21
_diffrn_reflns_limit_l_max        30
_diffrn_reflns_theta_min          3.782
_diffrn_reflns_theta_max          76.864
_diffrn_reflns_theta_full         67.679
_diffrn_measured_fraction_theta_max   0.930
_diffrn_measured_fraction_theta_full  0.968
_diffrn_reflns_Laue_measured_fraction_max    0.930
_diffrn_reflns_Laue_measured_fraction_full   0.968
_diffrn_reflns_point_group_measured_fraction_max   0.930
_diffrn_reflns_point_group_measured_fraction_full  0.968
_reflns_number_total              3688
```

```
_reflns_number_gt                2961
_reflns_threshold_expression     'I > 2\s(I)'
_reflns_Friedel_coverage         0.000
_reflns_Friedel_fraction_max     .
_reflns_Friedel_fraction_full    .

_reflns_special_details
;
 Reflections were merged by SHELXL according to the crystal
 class for the calculation of statistics and refinement.

 _reflns_Friedel_fraction is defined as the number of unique
 Friedel pairs measured divided by the number that would be
 possible theoretically, ignoring centric projections and
 systematic absences.
;

_computing_data_collection
;
CrysAlisPro, Agilent Technologies,
Version 1.171.37.34 (release 22-05-2014 CrysAlis171 .NET)
(compiled May 22 2014,16:03:01)
;
_computing_cell_refinement
;
CrysAlisPro, Agilent Technologies,
Version 1.171.37.34 (release 22-05-2014 CrysAlis171 .NET)
(compiled May 22 2014,16:03:01)
;
_computing_data_reduction
;
CrysAlisPro, Agilent Technologies,
Version 1.171.37.34 (release 22-05-2014 CrysAlis171 .NET)
(compiled May 22 2014,16:03:01)
;
_computing_structure_solution     'SHELXS-2013/1 (Sheldrick, 2014)'
_computing_structure_refinement   'SHELXL-2014/7 (Sheldrick, 2014)'
_computing_molecular_graphics     'ORTEP3 for Windows'
_computing_publication_material   'SHELXL-2014/7 and WINGX'
_refine_special_details           ?
_refine_ls_structure_factor_coef  Fsqd
_refine_ls_matrix_type            full
_refine_ls_weighting_scheme       calc
_refine_ls_weighting_details
'w=1/[\s^2^(Fo^2^)+(0.0816P)^2^+8.5104P] where P=(Fo^2^+2Fc^2^)/3'
_atom_sites_solution_primary      difmap
_atom_sites_solution_secondary    difmap
_atom_sites_solution_hydrogens    geom
_refine_ls_hydrogen_treatment     constr
_refine_ls_extinction_method      none
_refine_ls_extinction_coef        .
_refine_ls_number_reflns          3688
_refine_ls_number_parameters      244
_refine_ls_number_restraints      0
_refine_ls_R_factor_all           0.0820
_refine_ls_R_factor_gt            0.0603
_refine_ls_wR_factor_ref          0.1810
_refine_ls_wR_factor_gt           0.1595
_refine_ls_goodness_of_fit_ref    1.092
_refine_ls_restrained_S_all       1.092
_refine_ls_shift/su_max           0.000
```

```
_refine_ls_shift/su_mean          0.000

loop_
 _atom_site_label
 _atom_site_type_symbol
 _atom_site_fract_x
 _atom_site_fract_y
 _atom_site_fract_z
 _atom_site_U_iso_or_equiv
 _atom_site_adp_type
 _atom_site_occupancy
 _atom_site_site_symmetry_order
 _atom_site_calc_flag
 _atom_site_refinement_flags_posn
 _atom_site_refinement_flags_adp
 _atom_site_refinement_flags_occupancy
 _atom_site_disorder_assembly
 _atom_site_disorder_group
N1 N 0.41104(17) 0.3488(3) 0.50088(12) 0.0267(6) Uani 1 1 d . . . . .
N2 N 0.37104(18) 0.3236(3) 0.44623(13) 0.0291(6) Uani 1 1 d . . . . .
N4 N 0.47767(17) 0.1760(3) 0.43393(12) 0.0240(6) Uani 1 1 d . . . . .
S7 S 0.34932(5) 0.16850(9) 0.34905(4) 0.0331(2) Uani 1 1 d . . . . .
S8 S 0.23394(5) 0.18628(9) 0.35102(4) 0.0354(3) Uani 1 1 d . . . . .
Cl Cl 0.11629(7) 0.82356(11) 0.29089(5) 0.0476(3) Uani 1 1 d . . . . .
C3 C 0.40411(19) 0.2296(3) 0.41801(14) 0.0242(6) Uani 1 1 d . . . . .
C5 C 0.52105(18) 0.2081(3) 0.48750(13) 0.0204(6) Uani 1 1 d . . . . .
C6 C 0.48247(18) 0.2875(3) 0.52263(14) 0.0219(6) Uani 1 1 d . . . . .
C9 C 0.20539(19) 0.3684(4) 0.33122(13) 0.0249(6) Uani 1 1 d . . . . .
C10 C 0.2538(2) 0.4709(4) 0.31577(16) 0.0317(8) Uani 1 1 d . . . . .
H10 H 0.3050 0.4461 0.3141 0.047 Uiso 1 1 calc R U . . .
C11 C 0.2256(2) 0.6110(4) 0.30274(16) 0.0330(8) Uani 1 1 d . . . . .
H11 H 0.2581 0.6812 0.2928 0.050 Uiso 1 1 calc R U . . .
C12 C 0.1492(2) 0.6456(4) 0.30453(15) 0.0311(7) Uani 1 1 d . . . . .
C13 C 0.0998(2) 0.5435(4) 0.31917(16) 0.0342(8) Uani 1 1 d . . . . .
H13 H 0.0482 0.5679 0.3199 0.051 Uiso 1 1 calc R U . . .
C14 C 0.1287(2) 0.4037(4) 0.33276(15) 0.0315(7) Uani 1 1 d . . . . .
H14 H 0.0964 0.3336 0.3429 0.047 Uiso 1 1 calc R U . . .
C51 C 0.60423(19) 0.1576(3) 0.50432(13) 0.0218(6) Uani 1 1 d . . . . .
C52 C 0.62436(19) 0.0323(3) 0.47809(14) 0.0247(7) Uani 1 1 d . . . . .
H52 H 0.5854 -0.0149 0.4501 0.037 Uiso 1 1 calc R U . . .
C53 C 0.7015(2) -0.0216(4) 0.49352(16) 0.0302(7) Uani 1 1 d . . . . .
H53 H 0.7142 -0.1049 0.4761 0.045 Uiso 1 1 calc R U . . .
C54 C 0.7600(2) 0.0493(4) 0.53518(16) 0.0320(8) Uani 1 1 d . . . . .
H54 H 0.8116 0.0124 0.5461 0.048 Uiso 1 1 calc R U . . .
C55 C 0.7412(2) 0.1759(4) 0.56057(15) 0.0313(8) Uani 1 1 d . . . . .
H55 H 0.7805 0.2239 0.5881 0.047 Uiso 1 1 calc R U . . .
C56 C 0.66426(19) 0.2300(4) 0.54500(14) 0.0262(7) Uani 1 1 d . . . . .
H56 H 0.6523 0.3151 0.5617 0.039 Uiso 1 1 calc R U . . .
C61 C 0.51329(18) 0.3064(3) 0.58532(14) 0.0220(6) Uani 1 1 d . . . . .
C62 C 0.54969(19) 0.1918(3) 0.62042(14) 0.0224(6) Uani 1 1 d . . . . .
H62 H 0.5566 0.1030 0.6041 0.034 Uiso 1 1 calc R U . . .
C63 C 0.57583(19) 0.2089(4) 0.67954(14) 0.0265(7) Uani 1 1 d . . . . .
H63 H 0.5999 0.1320 0.7026 0.040 Uiso 1 1 calc R U . . .
C64 C 0.5656(2) 0.3424(4) 0.70365(15) 0.0314(7) Uani 1 1 d . . . . .
H64 H 0.5835 0.3549 0.7431 0.047 Uiso 1 1 calc R U . . .
C65 C 0.5289(2) 0.4567(4) 0.66945(16) 0.0317(8) Uani 1 1 d . . . . .
H65 H 0.5222 0.5454 0.6859 0.048 Uiso 1 1 calc R U . . .
C66 C 0.50232(19) 0.4388(4) 0.61066(15) 0.0277(7) Uani 1 1 d . . . . .
H66 H 0.4770 0.5152 0.5879 0.042 Uiso 1 1 calc R U . . .

loop_
```

_atom_site_aniso_label
_atom_site_aniso_U_11
_atom_site_aniso_U_22
_atom_site_aniso_U_33
_atom_site_aniso_U_23
_atom_site_aniso_U_13
_atom_site_aniso_U_12
N1 0.0267(13) 0.0268(13) 0.0257(14) 0.0000(11) 0.0055(11) 0.0062(11)
N2 0.0293(14) 0.0297(14) 0.0268(14) -0.0020(11) 0.0048(12) 0.0079(11)
N4 0.0308(14) 0.0211(12) 0.0215(13) 0.0022(10) 0.0094(11) 0.0036(10)
S7 0.0376(5) 0.0305(4) 0.0260(4) -0.0036(3) -0.0005(3) 0.0113(3)
S8 0.0341(4) 0.0270(4) 0.0389(5) 0.0068(3) -0.0009(4) -0.0022(3)
Cl 0.0566(6) 0.0392(5) 0.0453(6) 0.0071(4) 0.0105(5) 0.0230(4)
C3 0.0263(15) 0.0223(14) 0.0232(15) 0.0019(12) 0.0051(12) 0.0035(12)
C5 0.0249(14) 0.0170(13) 0.0199(14) 0.0018(11) 0.0071(12) -0.0004(11)
C6 0.0235(14) 0.0188(13) 0.0261(16) -0.0029(11) 0.0111(12) 0.0026(11)
C9 0.0267(15) 0.0294(15) 0.0174(14) 0.0012(12) 0.0037(12) 0.0007(13)
C10 0.0228(15) 0.0306(17) 0.042(2) 0.0073(14) 0.0084(14) 0.0040(13)
C11 0.0284(16) 0.0333(17) 0.039(2) 0.0073(15) 0.0110(15) 0.0009(14)
C12 0.0354(17) 0.0315(16) 0.0243(16) -0.0027(13) 0.0044(14) 0.0094(14)
C13 0.0237(15) 0.049(2) 0.0325(19) -0.0011(15) 0.0122(14) 0.0076(15)
C14 0.0265(16) 0.0408(19) 0.0299(17) 0.0018(15) 0.0124(14) -0.0076(14)
C51 0.0254(15) 0.0227(14) 0.0210(15) 0.0053(11) 0.0127(12) 0.0029(12)
C52 0.0280(15) 0.0242(14) 0.0254(16) 0.0004(12) 0.0129(13) 0.0027(12)
C53 0.0294(16) 0.0303(16) 0.0353(18) 0.0038(14) 0.0162(14) 0.0066(13)
C54 0.0268(16) 0.0412(19) 0.0326(18) 0.0096(15) 0.0157(14) 0.0091(14)
C55 0.0245(15) 0.047(2) 0.0237(16) 0.0046(14) 0.0095(13) -0.0016(14)
C56 0.0263(15) 0.0330(16) 0.0220(15) -0.0006(13) 0.0113(13) 0.0007(13)
C61 0.0205(13) 0.0209(13) 0.0253(16) -0.0041(12) 0.0072(12) 0.0007(11)
C62 0.0269(14) 0.0221(14) 0.0231(16) -0.0002(11) 0.0148(13) 0.0000(12)
C63 0.0263(15) 0.0327(16) 0.0231(16) 0.0018(13) 0.0110(13) -0.0003(13)
C64 0.0281(16) 0.0426(19) 0.0246(16) -0.0079(14) 0.0088(13) -0.0017(14)
C65 0.0295(16) 0.0319(17) 0.0353(19) -0.0149(14) 0.0115(14) 0.0003(14)
C66 0.0260(15) 0.0244(15) 0.0335(18) -0.0065(13) 0.0093(14) 0.0049(12)

_geom_special_details
;
All esds (except the esd in the dihedral angle between two l.s. planes)
are estimated using the full covariance matrix. The cell esds are taken
into account individually in the estimation of esds in distances, angles
and torsion angles; correlations between esds in cell parameters are only
used when they are defined by crystal symmetry. An approximate (isotropic)
treatment of cell esds is used for estimating esds involving l.s. planes.
;

loop_
_geom_bond_atom_site_label_1
_geom_bond_atom_site_label_2
_geom_bond_distance
_geom_bond_site_symmetry_2
_geom_bond_publ_flag
N1 C6 1.339(4) . ?
N1 N2 1.343(4) . ?
N2 C3 1.326(4) . ?
N4 C3 1.329(4) . ?
N4 C5 1.350(4) . ?
S7 C3 1.781(3) . ?
S7 S8 2.0271(14) . ?
S8 C9 1.778(3) . ?
Cl C12 1.737(4) . ?
C5 C6 1.419(4) . ?

C5 C51 1.470(4) . ?
C6 C61 1.482(4) . ?
C9 C14 1.383(5) . ?
C9 C10 1.381(5) . ?
C10 C11 1.385(5) . ?
C10 H10 0.9300 . ?
C11 C12 1.380(5) . ?
C11 H11 0.9300 . ?
C12 C13 1.381(5) . ?
C13 C14 1.389(5) . ?
C13 H13 0.9300 . ?
C14 H14 0.9300 . ?
C51 C56 1.400(5) . ?
C51 C52 1.406(4) . ?
C52 C53 1.386(5) . ?
C52 H52 0.9300 . ?
C53 C54 1.391(5) . ?
C53 H53 0.9300 . ?
C54 C55 1.397(5) . ?
C54 H54 0.9300 . ?
C55 C56 1.382(5) . ?
C55 H55 0.9300 . ?
C56 H56 0.9300 . ?
C61 C62 1.397(4) . ?
C61 C66 1.401(4) . ?
C62 C63 1.393(5) . ?
C62 H62 0.9300 . ?
C63 C64 1.393(5) . ?
C63 H63 0.9300 . ?
C64 C65 1.386(5) . ?
C64 H64 0.9300 . ?
C65 C66 1.387(5) . ?
C65 H65 0.9300 . ?
C66 H66 0.9300 . ?

loop_
 _geom_angle_atom_site_label_1
 _geom_angle_atom_site_label_2
 _geom_angle_atom_site_label_3
 _geom_angle
 _geom_angle_site_symmetry_1
 _geom_angle_site_symmetry_3
 _geom_angle_publ_flag
C6 N1 N2 119.9(3) . . ?
C3 N2 N1 116.1(3) . . ?
C3 N4 C5 116.6(3) . . ?
C3 S7 S8 103.78(12) . . ?
C9 S8 S7 106.07(12) . . ?
N4 C3 N2 127.5(3) . . ?
N4 C3 S7 112.9(2) . . ?
N2 C3 S7 119.6(2) . . ?
N4 C5 C6 117.2(3) . . ?
N4 C5 C51 117.1(3) . . ?
C6 C5 C51 125.7(3) . . ?
N1 C6 C5 121.2(3) . . ?
N1 C6 C61 113.5(3) . . ?
C5 C6 C61 125.2(3) . . ?
C14 C9 C10 120.6(3) . . ?
C14 C9 S8 114.6(3) . . ?
C10 C9 S8 124.8(3) . . ?
C9 C10 C11 119.6(3) . . ?

C9 C10 H10 120.2 . . ?
C11 C10 H10 120.2 . . ?
C12 C11 C10 119.6(3) . . ?
C12 C11 H11 120.2 . . ?
C10 C11 H11 120.2 . . ?
C11 C12 C13 121.4(3) . . ?
C11 C12 Cl 118.9(3) . . ?
C13 C12 Cl 119.7(3) . . ?
C12 C13 C14 118.8(3) . . ?
C12 C13 H13 120.6 . . ?
C14 C13 H13 120.6 . . ?
C9 C14 C13 120.1(3) . . ?
C9 C14 H14 120.0 . . ?
C13 C14 H14 120.0 . . ?
C56 C51 C52 118.7(3) . . ?
C56 C51 C5 122.7(3) . . ?
C52 C51 C5 118.6(3) . . ?
C53 C52 C51 120.8(3) . . ?
C53 C52 H52 119.6 . . ?
C51 C52 H52 119.6 . . ?
C52 C53 C54 119.8(3) . . ?
C52 C53 H53 120.1 . . ?
C54 C53 H53 120.1 . . ?
C53 C54 C55 120.0(3) . . ?
C53 C54 H54 120.0 . . ?
C55 C54 H54 120.0 . . ?
C56 C55 C54 120.2(3) . . ?
C56 C55 H55 119.9 . . ?
C54 C55 H55 119.9 . . ?
C55 C56 C51 120.5(3) . . ?
C55 C56 H56 119.7 . . ?
C51 C56 H56 119.7 . . ?
C62 C61 C66 118.8(3) . . ?
C62 C61 C6 121.2(3) . . ?
C66 C61 C6 119.9(3) . . ?
C63 C62 C61 120.9(3) . . ?
C63 C62 H62 119.6 . . ?
C61 C62 H62 119.6 . . ?
C64 C63 C62 119.2(3) . . ?
C64 C63 H63 120.4 . . ?
C62 C63 H63 120.4 . . ?
C65 C64 C63 120.6(3) . . ?
C65 C64 H64 119.7 . . ?
C63 C64 H64 119.7 . . ?
C64 C65 C66 120.0(3) . . ?
C64 C65 H65 120.0 . . ?
C66 C65 H65 120.0 . . ?
C65 C66 C61 120.5(3) . . ?
C65 C66 H66 119.8 . . ?
C61 C66 H66 119.8 . . ?

loop_
 _geom_torsion_atom_site_label_1
 _geom_torsion_atom_site_label_2
 _geom_torsion_atom_site_label_3
 _geom_torsion_atom_site_label_4
 _geom_torsion
 _geom_torsion_site_symmetry_1
 _geom_torsion_site_symmetry_2
 _geom_torsion_site_symmetry_3
 _geom_torsion_site_symmetry_4

_geom_torsion_publ_flag
C6 N1 N2 C3 4.2(5) . . . . ?
C5 N4 C3 N2 7.9(5) . . . . ?
C5 N4 C3 S7 -175.0(2) . . . . ?
N1 N2 C3 N4 -12.2(5) . . . . ?
N1 N2 C3 S7 170.9(2) . . . . ?
S8 S7 C3 N4 155.4(2) . . . . ?
S8 S7 C3 N2 -27.3(3) . . . . ?
C3 N4 C5 C6 3.9(4) . . . . ?
C3 N4 C5 C51 -176.1(3) . . . . ?
N2 N1 C6 C5 6.7(5) . . . . ?
N2 N1 C6 C61 -170.9(3) . . . . ?
N4 C5 C6 N1 -10.9(4) . . . . ?
C51 C5 C6 N1 169.1(3) . . . . ?
N4 C5 C6 C61 166.4(3) . . . . ?
C51 C5 C6 C61 -13.6(5) . . . . ?
S7 S8 C9 C14 -177.1(2) . . . . ?
S7 S8 C9 C10 2.3(3) . . . . ?
C14 C9 C10 C11 1.2(5) . . . . ?
S8 C9 C10 C11 -178.2(3) . . . . ?
C9 C10 C11 C12 -1.0(6) . . . . ?
C10 C11 C12 C13 0.1(6) . . . . ?
C10 C11 C12 Cl 177.4(3) . . . . ?
C11 C12 C13 C14 0.6(6) . . . . ?
Cl C12 C13 C14 -176.7(3) . . . . ?
C10 C9 C14 C13 -0.5(5) . . . . ?
S8 C9 C14 C13 178.9(3) . . . . ?
C12 C13 C14 C9 -0.3(5) . . . . ?
N4 C5 C51 C56 151.3(3) . . . . ?
C6 C5 C51 C56 -28.6(5) . . . . ?
N4 C5 C51 C52 -28.1(4) . . . . ?
C6 C5 C51 C52 152.0(3) . . . . ?
C56 C51 C52 C53 2.2(5) . . . . ?
C5 C51 C52 C53 -178.4(3) . . . . ?
C51 C52 C53 C54 -0.4(5) . . . . ?
C52 C53 C54 C55 -1.1(5) . . . . ?
C53 C54 C55 C56 0.9(5) . . . . ?
C54 C55 C56 C51 0.9(5) . . . . ?
C52 C51 C56 C55 -2.4(5) . . . . ?
C5 C51 C56 C55 178.1(3) . . . . ?
N1 C6 C61 C62 137.1(3) . . . . ?
C5 C6 C61 C62 -40.4(5) . . . . ?
N1 C6 C61 C66 -39.5(4) . . . . ?
C5 C6 C61 C66 143.0(3) . . . . ?
C66 C61 C62 C63 -0.9(5) . . . . ?
C6 C61 C62 C63 -177.6(3) . . . . ?
C61 C62 C63 C64 -0.2(5) . . . . ?
C62 C63 C64 C65 0.8(5) . . . . ?
C63 C64 C65 C66 -0.2(5) . . . . ?
C64 C65 C66 C61 -1.0(5) . . . . ?
C62 C61 C66 C65 1.6(5) . . . . ?
C6 C61 C66 C65 178.2(3) . . . . ?

_refine_diff_density_max 0.833
_refine_diff_density_min -0.531
_refine_diff_density_rms 0.092

_shelx_res_file
;

shelx.res created by SHELXL-2014/7

```
TITL mn_13 in C2/c
CELL  1.54178  17.3965   9.2049  24.2516   90.000  105.435   90.000
ZERR     8.00   0.0019   0.0006   0.0003    0.000    0.013    0.000
LATT   7
SYMM  - X,   Y, 1/2 - Z
SFAC  C    H    N    S    CL
UNIT  168 112 24 16 8
MERG   2
FMAP   2
OMIT   -12   2  24
OMIT   -11   3  26
OMIT   -11   3  25
OMIT   -12   2  23
OMIT   -12   0  20
OMIT   -10   2  27
OMIT   -11   1  24
OMIT    -9   5  25
OMIT   -10   4  26
OMIT    -8   4  28
OMIT    -9   3  28
OMIT   -11   1  23
OMIT    -9   3  27
OMIT    -7   3  29
OMIT   -11   3  24
OMIT    -7   5  27
OMIT    -8   4  26
OMIT    -6   0  30
OMIT   -10   4  25
OMIT    -7   5  26
OMIT    -6   6  25
OMIT    -9   7   1
OMIT   -11   1  21
OMIT   -11   1  22
PLAN   -2
ACTA
BOND   $H
CONF
L.S.  50
WGHT    0.081600    8.510400
FVAR       2.42081
N1    3    0.411038    0.348790    0.500883    11.00000    0.02666    0.02680 =
         0.02574    0.00000    0.00552    0.00616
N2    3    0.371039    0.323609    0.446230    11.00000    0.02932    0.02972 =
         0.02681   -0.00205    0.00484    0.00788
N4    3    0.477674    0.176031    0.433934    11.00000    0.03081    0.02110 =
         0.02146    0.00215    0.00937    0.00363
S7    4    0.349322    0.168500    0.349051    11.00000    0.03759    0.03049 =
         0.02604   -0.00361   -0.00052    0.01131
S8    4    0.233936    0.186276    0.351016    11.00000    0.03405    0.02701 =
         0.03894    0.00684   -0.00090   -0.00224
CL    5    0.116290    0.823557    0.290894    11.00000    0.05664    0.03919 =
         0.04527    0.00707    0.01052    0.02300
C3    1    0.404108    0.229557    0.418014    11.00000    0.02628    0.02232 =
         0.02319    0.00191    0.00508    0.00355
C5    1    0.521047    0.208051    0.487503    11.00000    0.02489    0.01702 =
         0.01991    0.00176    0.00710   -0.00044
C6    1    0.482469    0.287487    0.522626    11.00000    0.02350    0.01876 =
         0.02610   -0.00288    0.01113    0.00262
C9    1    0.205394    0.368435    0.331216    11.00000    0.02672    0.02940 =
```

```
         0.01742    0.00123    0.00369    0.00067
C10   1    0.253805    0.470926    0.315769    11.00000    0.02279    0.03061 =
         0.04150    0.00727    0.00839    0.00404
AFIX  43
H10   2    0.304968    0.446093    0.314128    11.00000   -1.50000
AFIX   0
C11   1    0.225641    0.610969    0.302737    11.00000    0.02843    0.03325 =
         0.03860    0.00729    0.01099    0.00085
AFIX  43
H11   2    0.258052    0.681157    0.292844    11.00000   -1.50000
AFIX   0
C12   1    0.149162    0.645618    0.304534    11.00000    0.03536    0.03148 =
         0.02431   -0.00274    0.00437    0.00939
C13   1    0.099839    0.543480    0.319173    11.00000    0.02375    0.04898 =
         0.03245   -0.00107    0.01216    0.00762
AFIX  43
H13   2    0.048248    0.567885    0.319916    11.00000   -1.50000
AFIX   0
C14   1    0.128720    0.403652    0.332763    11.00000    0.02647    0.04079 =
         0.02991    0.00178    0.01240   -0.00760
AFIX  43
H14   2    0.096448    0.333625    0.342916    11.00000   -1.50000
AFIX   0
C51   1    0.604225    0.157625    0.504325    11.00000    0.02536    0.02275 =
         0.02096    0.00525    0.01267    0.00292
C52   1    0.624358    0.032314    0.478091    11.00000    0.02796    0.02416 =
         0.02541    0.00044    0.01295    0.00271
AFIX  43
H52   2    0.585441   -0.014946    0.450054    11.00000   -1.50000
AFIX   0
C53   1    0.701527   -0.021629    0.493521    11.00000    0.02942    0.03025 =
         0.03532    0.00377    0.01625    0.00664
AFIX  43
H53   2    0.714156   -0.104945    0.476076    11.00000   -1.50000
AFIX   0
C54   1    0.760007    0.049265    0.535181    11.00000    0.02685    0.04117 =
         0.03258    0.00957    0.01573    0.00914
AFIX  43
H54   2    0.811624    0.012426    0.546129    11.00000   -1.50000
AFIX   0
C55   1    0.741155    0.175871    0.560573    11.00000    0.02453    0.04748 =
         0.02371    0.00460    0.00953   -0.00164
AFIX  43
H55   2    0.780476    0.223915    0.588050    11.00000   -1.50000
AFIX   0
C56   1    0.664264    0.229964    0.544996    11.00000    0.02631    0.03298 =
         0.02204   -0.00064    0.01129    0.00068
AFIX  43
H56   2    0.652339    0.315071    0.561668    11.00000   -1.50000
AFIX   0
C61   1    0.513292    0.306370    0.585323    11.00000    0.02051    0.02089 =
         0.02533   -0.00408    0.00715    0.00071
C62   1    0.549689    0.191825    0.620425    11.00000    0.02685    0.02205 =
         0.02306   -0.00023    0.01483    0.00003
AFIX  43
H62   2    0.556565    0.102980    0.604108    11.00000   -1.50000
AFIX   0
C63   1    0.575825    0.208874    0.679538    11.00000    0.02629    0.03268 =
         0.02310    0.00176    0.01098   -0.00029
AFIX  43
H63   2    0.599858    0.131991    0.702642    11.00000   -1.50000
```

```
AFIX   0
C64   1    0.565564    0.342357    0.703655    11.00000    0.02813    0.04260 =
         0.02458   -0.00787    0.00878   -0.00171
AFIX  43
H64   2    0.583453    0.354883    0.743056    11.00000   -1.50000
AFIX   0
C65   1    0.528926    0.456683    0.669453    11.00000    0.02950    0.03192 =
         0.03535   -0.01494    0.01148    0.00033
AFIX  43
H65   2    0.522187    0.545389    0.685921    11.00000   -1.50000
AFIX   0
C66   1    0.502316    0.438769    0.610660    11.00000    0.02600    0.02441 =
         0.03350   -0.00645    0.00933    0.00488
AFIX  43
H66   2    0.476996    0.515231    0.587888    11.00000   -1.50000
AFIX   0
HKLF    4

REM  mn_13 in C2/c
REM R1 =  0.0603 for    2961 Fo > 4sig(Fo)  and  0.0820 for all    3688 data
REM    244 parameters refined using      0 restraints

END
```
